# Fosfomycin as Partner Drug for Systemic Infection Management. A Systematic Review of Its Synergistic Properties from In Vitro and In Vivo Studies

**DOI:** 10.3390/antibiotics9080500

**Published:** 2020-08-10

**Authors:** Roberta Maria Antonello, Luigi Principe, Alberto Enrico Maraolo, Valentina Viaggi, Riccardo Pol, Massimiliano Fabbiani, Francesca Montagnani, Antonio Lovecchio, Roberto Luzzati, Stefano Di Bella

**Affiliations:** 1 Clinical Department of Medical, Surgical and Health Sciences, Trieste University, 34127 Trieste, Italy; rma.roby@gmail.com (R.M.A.); antolvc93@gmail.com (A.L.); roberto.luzzati@asugi.sanita.fvg.it (R.L.); 2 “San Giovanni di Dio” Hospital, 88900 Crotone, Italy; luigi.principe@gmail.com; 3 First Division of Infectious Diseases, Cotugno Hospital, AORN dei Colli, 80131 Naples, Italy; albertomaraolo@mail.com; 4 “A. Manzoni” Hospital, 23900 Lecco, Italy; v.viaggi@asst-lecco.it; 5 Department of Infectious Diseases, Udine University, 33100 Udine, Italy; riccardopol91@gmail.com; 6 Department of Medical Sciences, Tropical and Infectious Diseases Unit, University Hospital of Siena, 53100 Siena, Italy; massimiliano.fabbiani@gmail.com (M.F.); francesca.montagnani@unisi.it (F.M.); 7 Department of Medical Biotechnologies, University of Siena, 53100 Siena, Italy

**Keywords:** fosfomycin, pharmacodynamic, synergic, synergism, synergistic, infection, multidrug resistant

## Abstract

Fosfomycin is being increasingly prescribed for multidrug-resistant bacterial infections. In patients with systemic involvement, intravenous fosfomycin is usually administered as a partner drug, as part of an antibiotic regimen. Hence, the knowledge of fosfomycin pharmacodynamic interactions (synergistic, additive, indifferent and antagonistic effect) is fundamental for a proper clinical management of severe bacterial infections. We performed a systematic review to point out fosfomycin’s synergistic properties, when administered with other antibiotics, in order to help clinicians to maximize drug efficacy optimizing its use in clinical practice. Interactions were more frequently additive or indifferent (65.4%). Synergism accounted for 33.7% of total interactions, while antagonism occurred sporadically (0.9%). Clinically significant synergistic interactions were mostly distributed in combination with penicillins (51%), carbapenems (43%), chloramphenicol (39%) and cephalosporins (33%) in Enterobactaerales; with linezolid (74%), tetracyclines (72%) and daptomycin (56%) in *Staphylococcus aureus*; with chloramphenicol (53%), aminoglycosides (43%) and cephalosporins (36%) against *Pseudomonas aeruginosa*; with daptomycin (97%) in *Enterococcus* spp. and with sulbactam (75%) and penicillins (60%) and in *Acinetobacter* spp. fosfomycin-based antibiotic associations benefit from increase in the bactericidal effect and prevention of antimicrobial resistances. Taken together, the presence of synergistic interactions and the nearly total absence of antagonisms, make fosfomycin a good partner drug in clinical practice.

## 1. Introduction

Antimicrobial resistance (AMR) is a health issue of global concern, burdened with elevated costs and high morbidity and mortality rates. Limited therapeutic options and the increasing occurrence of resistance to last-resort antibiotics, i.e., colistin or carbapenems, make it necessary to reassess the role of “old” drugs while waiting for new antibiotics available on the market.

Fosfomycin (FOS) is an inhibitor of the synthesis of the bacterial wall acting with a unique mechanism of action. To carry out its action, FOS enters in the bacterial cell through the L-alpha-glycerophosphate and the hexose-6-phosphate transporter systems, interfering with the formation of the peptidoglycan precursor uridine diphosphate N-acetylmuramic acid (UDP-MurNAc) [1].

FOS, after being discovered in 1969 [2], has long been prescribed orally for low urinary tract infections (UTIs) and only recently has been repurposed, also intravenously and in combination, as a meropenem- and colistin-sparing agent to treat other infections (complicated UTIs, severe soft tissue infections, osteomyelitis, prostatitis, etc.) [1,3,4,5]. The excellent distribution in body sites, the safety and tolerability profile, as well as its affordability, make FOS a therapeutic option worth considering to treat multidrug-resistant (MDR) bacterial infections [6,7].

FOS is generally prescribed in association with at least another active agent. The association benefits from increase in the bactericidal effect of FOS, prevention of AMR, limitation of side effects thanks to lower dosages. Examples of commonly used empirical combination regimens including FOS are: Carbapenems + FOS, colistin + FOS, ceftolozane/tazobactam + FOS and tigecycline (TIG) + FOS.

We performed a systematic literature review concerning in vitro and in vivo studies to evaluate the synergistic effect of FOS in combination with other antibiotics and offer an overall view with clinically practical tables divided by antibiotic class.

## 2. Materials and Methods

This systematic review was carried out following the Preferred Reporting Items for Systematic reviews and Meta-Analyses (PRISMA).

On 14 April 2020 we performed a MEDLINE/PubMed search using the search string “Fosfomycin"[Tw] AND (synerg*[Tw] OR association*[Tw] OR combin*[Tw] OR “together”[Tw] OR “additive”[Tw] OR “addition”[Tw] OR “checkerboard”[Tw] OR “chequerboard”[Tw] OR “time kill”[Tw] OR “time–kill”[Tw] OR “time–killing”[Tw] OR “time killing”[Tw])”.

1232 papers, from inception to 14 April 2020, were identified. Of these, 870 were excluded by title screening, 84 by abstract screening, 28 after full-text reading. Fifty-eight papers were excluded because written in a language different from English. 7 papers were excluded because full text was not available either online or in paper version. 185 papers were reviewed and discussed independently by seven authors (RMA, RP, AL, SDB, VV, LP, MF). 

Common criteria for the evaluation of susceptibility and synergism were adopted by all authors. 

*Susceptibility*. Susceptibility to FOS for Enterobacterales and *Staphylococcus* spp. was determined, according to the European Committee on Antimicrobial Susceptibility Testing (EUCAST) breakpoints, when the minimum inhibitory concentration (MIC) was ≤ 32 µg/mL. *Enterococcus* spp. were considered susceptible when exhibiting a MIC ≤ 64 µg/mL, according to the Clinical & Laboratory Standards Institute (CLSI) breakpoints. FOS breakpoints are not defined either by EUCAST or CLSI for *Pseudomonas* spp., *Acinetobacter* spp. and *Streptococcus* spp. Based on literature data, susceptibility was defined as a MIC ≤ 128 µg/mL for *Pseudomonas* spp. (ECOFF value), MIC ≤ 32 for *Acinetobacter* spp. and ≤64 µg/mL for *Streptococcus* spp. [8,9].

For all the antibiotics tested in combination, EUCAST breakpoints was considered at first and CLSI breakpoints were considered when EUCAST breakpoints were not available. Breakpoints adopted are specified in each paragraph.

*Synergistic effect.* Checkerboard assay: fractional inhibitory concentration index (FICI) ≤ 0.5. FICI is defined as follows:

$$FICI=\frac{MIC\;FOS\;in\;combination}{MIC\;FOS\;alone}+\frac{MIC\;other\;antibiotic\;in\;combination}{MIC\;other\;antibiotic\;alone}$$.

Time–kill assay: ratio of effective concentrations concordant with FICI or ≥2 log kill.

*Additive effect.* Checkerboard assay: 0.5 < FICI ≤ 1. Time–kill assay: ratio of effective concentrations concordant with FICI or 1 < log kill < 2.

*Indifferent effect.* Checkerboard assay: 1 < FICI < 4. Time–kill assay: ratio of effective concentrations concordant with FICI or ± 1 log kill.

*Antagonistic effect.* Checkerboard assay: FICI ≥ 4. Time–kill assay: ratio of effective concentrations concordant with FICI or < 1 log kill.

For in vitro studies using a method different from checkerboard or time–kill assay, or in case data on effective concentrations were not available, synergism was evaluated according to the authors’ judgment.

For studies performed in vivo, synergism was established with the same ratio of effective concentrations considered for checkerboard assays or with the same log kill considered for time–kill assays. When these data were not reported in the paper, synergism was evaluated according to the authors’ judgment.

## 3. Results

For a better comprehension, a table with reviewed papers and a summary of most relevant results is proposed for each antibiotic class.

### 3.1. Penicillins

Twenty-eight papers evaluating FOS in combination with penicillins, penicillins + β-lactamase inhibitors, penicillinase-resistant penicillins were reviewed (Table 1). Breakpoints for penicillins were inferred from EUCAST breakpoints [10]. Penicillins are β-lactam antibiotics that acts through the inhibition of enzymes needed for peptidoglycans cross linking. Effect of FOS in combination with penicillins varied greatly according with the bacterial species considered. The highest rates of synergistic effect were observed against Enterobacterales and *Acinetobacter* spp. Despite this, Avery et al. [11] reported high rates of indifferent effect of FOS + piperacillin/tazobactam (PIP/TAZ) against PIP/TAZ-resistant Enterobacterales. Antagonistic effect was observed against one isolate of *S. aureus* with the combination FOS + methicillin [12] and against 6 biofilm-producer *Enterococcus faecalis* isolates with the combination FOS + ampicillin [13]. Four studies [14,15,16,17] performed in vivo experiments, with no substantial differences in results when compared with results obtained in vitro.

The combination of penicillin + FOS retains additive/synergistic effects against ~50% of Enterobacterales, *Acinetobacter* spp., *Staphylococcus* spp., and *Streptococcus* spp. strains.

### 3.2. Cephalosporins

Forty-one papers evaluating FOS in combination with cephalosporins and cephalosporins + β-lactamase inhibitors were reviewed (Table 2). Breakpoints for cephalosporins were inferred from EUCAST breakpoints [10]. Cephalosporins are β-lactam antibiotics that acts disrupting the peptidoglycan synthesis like penicillins, but are less susceptible to β-lactamases. Some studies reported discordant results on the effect of FOS in combination with a cephalosporin against clinical isolates, particularly against *Staphylococcus* spp. [18,19,20] and Enterobacterales isolates [11,14,21]. Antagonistic effect was observed against 4 *Pseudomonas aeruginosa* isolates with the combination FOS + ceftazidime [22], 1 *S. aureus* and 1 *Staphylococcus epidermidis* isolates with the combination FOS + ceftriaxone [19]. 9 in vivo studies [17,23,24,25,26,27,28,29,30] performed with different strains (*Escherichia coli*, *P. aeruginosa*, *S. aureus*, *Streptococcus pneumoniae*, *Streptococcus sanguis*) confirmed results obtained in vitro or resulted in higher synergistic effect (additive effect only against 3 *S. aureus* isolates [25,26]).

Cephalosporins + β-lactamase inhibitors, often chosen by clinicians to treat MDR infections, resulted in moderate rates of synergistic effect in combination with FOS. Against Enterobacterales, the combination ceftolozane/tazobactam + FOS resulted synergistic in 16.3% of cases (49 isolates tested [11]), while the combination ceftazidime/avibactam + FOS was synergistic in 28.8% of cases (66 isolates tested [11,21,31]). Against *P. aeruginosa*, the combination ceftolozane/tazobactam + FOS resulted synergistic in 71.1% of cases (45 isolates tested [32,33,34]), while the combination ceftazidime/avibactam + FOS was synergistic in 31.6% of cases (38 isolates tested [21,29,33]).

The combination of cephalosporins or cephalosporins + β-lactamase inhibitors + FOS appears to be clinically appealing especially against infections sustained by Enterobacterales and *Pseudomonas* spp.

### 3.3. Carbapenems

Forty-four papers evaluating FOS in combination with carbapenems were reviewed (Table 3). Carbapenems are β-lactam antibiotics that inhibit bacterial cell wall synthesis by binding to penicillin-binding proteins. Carbapenems are β-lactams “last-resort” used intravenously to treat severe infections. Imipenem (IMI) breakpoints are ≤ 2 µg/mL for Enterobacterales, *Acinetobacter* spp., *S. pneumoniae* and ≤ 0.001 µg/mL for *Pseudomonas* spp. and *Staphylococcus* spp. Meropenem breakpoints are ≤2 µg/mL for Enterobacterales, *Acinetobacter* spp., *Pseudomonas* spp., *S. pneumoniae* and ≤4 µg/mL for *Staphylococcus* spp. Ertapenem (ERT) breakpoints are ≤0.5 µg/mL for Enterobacterales, *S. pneumoniae* and ≤4 µg/mL for *Staphylococcus* spp. [10].

Synergism rates were not unanimous on all studies, but antagonistic effect was observed only in 2 isolates of *P. aeruginosa* in the study by Pruekprasert et al. [22] and in 1 isolate of *S. aureus* in the study by Quentin et al. [35]. No evident differences in the synergistic effect was observed depending on the carbapenem tested. The association FOS + carbapenem often resulted, when reported, in FOS- and/or carbapenem-susceptibility restoration. Three authors performed in vivo experiments using methicillin-resistant Staphylococcus aureus (MRSA) isolates: in two studies [28,36] the results in vivo were concordant with those found in vitro, while in the third study the combination in vivo resulted less effective [37].

From the clinical point of view the combination of carbapenems + FOS against Enterobacterales, *P. aeruginosa* end *Acinetobacter* spp. appears appealing.

### 3.4. Monobactams

Five papers evaluating FOS in combination with aztreonam (ATM) were reviewed (Table 4). ATM is a synthetic antibiotic whose susceptibility is often preserved also in those strains which are resistant to other β-lactam antibiotics. The mechanism of action is similar to penicillins. ATM breakpoints are ≤1 µg/mL for Enterobacterales and ≤0.001 µg/mL for *Pseudomonas* spp. [10].

The largest study evaluating FOS in combination with ATM on Enterobacterales isolates [33] reported an indifferent effect on most (64.6%) isolates. The combination was reported to have an additive effect on most isolates of *P. aeruginosa* [33,38], sometimes leading to ATM susceptibility restoration [33,39]. There were no in vivo studies evaluating this combination.

### 3.5. Quinolones

Twenty-nine papers evaluating FOS in combination with quinolones were reviewed (Table 5). Quinolones are bactericidal antibiotics that directly inhibit bacterial DNA synthesis. Breakpoints for quinolones were inferred from EUCAST breakpoints [10]. Synergism rates were not unanimous on all studies for isolates of *P. aeruginosa*. In 1 in vivo study synergism rate was 100% according to Mikuniya et al. [40]. Antagonism was observed in 1 in vivo [41] and 1 in vitro studies [39]. For *E. coli* isolates there was a weak synergism. In a recent in vitro study there was complete FOS and ciprofloxacin susceptibility restoration [42]. The combinations showed different synergistic rates for *Staphylococcus* spp. isolates with 100% synergistic rate in 1 in vitro study [43] and in 1 in vivo study [44]. No antagonism was observed for *E. coli* and *Staphylococcus* spp. isolates. There were some differences in the synergistic effect depending on the quinolone tested. The most frequent effect of FOS + ciprofloxacin was indifferent even though it showed in vitro 95% synergistic effect with *S. aureus* [45] The combination with levofloxacin showed mainly an additive effect in *P. aeruginosa* [38,39,46] and in *Acinetobacter* spp. [38] isolates.

In summary good additive/synergistic effect rates are reported when quinolones + FOS are used against *S. aureus* and *P. aeruginosa* isolates.

### 3.6. Aminoglycosides

Aminoglycosides (AMG) act through inhibition of protein synthesis, resulting in a potent and broad-spectrum antibacterial activity but with a potential high nephro- and oto-toxicity [47]. In the attempt to overcome increasing aminoglycosides resistance, development of novel AMG (such as arbekacin and plazomicin) has occurred, but combination strategies are important opportunities to treat resistant bacteria and to reduce toxicity. Inhaled delivery of tobramycin, allowing for greater exposure within the lungs and reducing systemic toxicity, is also approved for the treatment of patients with chronic *P. aeruginosa* lung infection associated with cystic fibrosis (CF) in United States and Europe [47]. Overall, 41 papers evaluating FOS in combinations with AMG were reviewed (Table 6). Available EUCAST aminoglycosides breakpoints were applied in all studies except one [48]. Due to the peculiarity of possible AMG therapeutic use (e.g. inhaled formulation in cystic fibrosis), many studies investigated the AMG + FOS combination also when administered by inhaled topical use; moreover, the activity of this combination on biofilm formation and in anaerobic conditions was also evaluated. Different AMG were tested as partner of FOS towards several bacterial species in a total of 67 evaluations: mainly gentamicin (31.3%, n = 21), amikacin (23.9%, n = 16) and tobramycin (22.4%, n = 15) were used. Synergism rates were not unanimous on all studies, considering the different bacteria analyzed and the different types of aminoglycosides tested. Overall, a synergistic effect of FOS together with different AMG, even if with different percentages, was revealed in 51 evaluations (74.6%). No synergism was reported in 16 cases (23.9%), even regarding effects on *P. aeruginosa* and *Acinetobacter* spp. In one study, data on synergism were not available [49]: however, a potential beneficial effect was indeed reported, demonstrating that FOS enhanced the activity of tobramycin with a 100% additive effect during in vitro evaluation on *P. aeruginosa* biofilms on cystic fibrosis airway epithelial cells. An antagonistic effect, testing the combination of FOS with gentamicin, was reported in 1985 by Alvarez et al. in 2.7% of 148 MRSA isolates [12] and in 2005 by Pruekprasert et al. in 27% of 22 *P. aeruginosa* strains [22].

Focusing on different bacterial strains, generally a synergistic or additive effect of FOS + AMG was demonstrated on KPC-producing *K. pneumoniae* [50,51,52]; however, Souli et al. observed an indifferent effect of FOS + gentamycin combination in all of their tested KPC+ strains [53].

When tested, a generally positive effect of FOS and AMG combination on biofilm formation and an improved AMG activity in anaerobic conditions were also reported for *P. aeruginosa* and *Acinetobacter* spp., resulting moreover in lower required AMG doses.

Activity of FOS plus an AMG was also evaluated against *Streptococcus* spp. (streptomycin) and *Neisseria gonorrhoeae* (both, gentamicin) in two studies [14,54]: No synergistic effect was revealed but antagonism was not even reported. Interestingly, synergistic activity (assessed as a fourfold reduction of MIC when fosfomycin was combined with gentamicin 1 mcg/mL) and additive effect were revealed for 8 vancomycin-resistant *E. faecium* (VRE) isolates (63% and 13%, respectively) [55].

The combination of AMG + FOS against *P. aeruginosa* appears to be the most clinically appealing.

### 3.7. Macrolides

Six papers evaluating FOS in combination with macrolides, in particular with erythromycin (ERY), azithromycin (AZT), clarithromycin (CLT), or midecamycin (MDM), were reviewed (Table 7). Macrolides are a large class of antibiotics that act binding 50S ribosomal subunit, inhibiting bacterial proteins synthesis. They have broad-spectrum activity, mainly against many Gram-positive bacteria and some Gram-negative bacteria [56]. Only one in vitro study evaluated FOS + ERY combination against Enterobacterales (87 strains of *E. cloacae, E. coli*, *Proteus* spp. and *Klebsiella pneumoniae*), reporting synergistic effect against 52% of isolates and additive effect against 30% [14]; in the same study FOS + ERY combination was also tested against *P. aeruginosa* and *S. aureus*, proving in most cases additive effect or, less frequently, synergistic effect [14]. When this combination was tested against *Streptococcus* spp. synergistic effect was observed against 15% of isolates, while additive (27%) or indifferent (58%) was seen against the remaining [14]. Some studies evaluated FOS + AZT combination, reporting indifferent effect in 100% of cases, either when tested against *N. gonorrhoeae* (2 studies) [54,57] or against *S. epidermidis* (1 study) [58]. Finally, FOS + CLT and FOS + MDM combinations were evaluated against *S. pseudointermedius* and *P. aeruginosa* respectively; in both cases additive or synergistic effect was demonstrated in vitro or in vivo experiments [59,60]. No antagonistic effect was observed for any combination against any isolate.

From the clinical point of view the combination of macrolides + FOS appears the less appealing.

### 3.8. Glycopeptides

Eighteen articles evaluating FOS in combination with glycopeptides (vancomycin and teicoplanin) have been reviewed (Table 8). Articles were from Spain (n = 5), Taiwan (n = 3), China (n = 2), France (n = 2), Germany (n = 2), Italy (n = 2), Austria (n = 1), and Brazil (n = 1). 

Glycopeptides possess an antimicrobial activity selectively directed against Gram-positive bacteria, while Gram-negatives are protected by the outer membrane that is impermeable to these antibiotics. Glycopeptides inhibit the peptidoglycan synthesis by interacting with the terminal D-alanyl-D-alanine present on the pentapeptide side chains of the peptidoglycan precursors. 

384 strains have been studied, belonging to several species as *S. aureus* (n = 219), *S. epidermidis* (n = 52), *E. faecalis* (n = 39), *S. pneumoniae* (n = 28), *Acinetobacter baumannii* (n = 20), *Enterococcus faecium* (n = 16) and other coagulase-negative staphylococci (CoNS) (n = 10). Synergy was detected with FOS-vancomycin (VAN) combination (40 out of 308 strains tested, 13%) in 33.3% of *E. faecalis*, 30% of *E. faecium*, 16.7% of *S. aureus*, 13.5% of *S. epidermidis*, and 3.6% of *S. pneumoniae*. Higher rates of synergistic interactions were detected with FOS-teicoplanin (TEC) combination (63 out of 130 strains tested, 48.5%) in 71.8% of *E. faecalis*, 43.7% of *E. faecium*, 60% of other CoNS, 34.3% of *S. aureus* and 33.3% *S. epidermidis*. Synergistic concentration ranges were 1-64 mg/L for FOS, 1-7.5 mg/L for VAN and only 8 mg/L for TEC. Regarding resistant isolates, FOS-VAN synergy was detected in one heterogeneous glycopeptide-intermediate *Staphylococcus aureus* (hGISA), 27 MRSA, 5 *S. aureus* strains with borderline MIC values for VAN (2 mg/L) and in 6 VRE strains, while FOS-TEC in 10 MRSA and 11 VRE strains. Antagonism FOS-VAN was detected in 5 *S. aureus* and one *S. epidermidis* strains. Only in 8 FOS-resistant *S. aureus* strains the activity of FOS was restored in combination with VAN. In vivo application of FOS-VAN combinations showed significant survival of ≥50% of treated animals or patients with infections caused by *S. aureus* or *S. epidermidis* [24,36,61,62,63].

In summary the combination of VAN + FOS resulted in good synergistic effect rates against *Enterococcus* spp. isolates and seems to be the most clinically relevant combination.

### 3.9. Tetracyclines

Ten papers evaluating FOS in combination with tetracyclines, mostly with minocycline (MIN) and in few cases with doxycycline (DOX) or tetracycline (TEC), were reviewed (Table 9). Tetracyclines are a large class of antibiotics that acts binding the 30S ribosomal subunits, inhibiting bacterial proteins synthesis. They have broad-spectrum activity, being active against many Gram-positive bacteria, Gram-negative, and atypical bacteria [64]. Almost all studies evaluated in vitro FOS + MIN combination against different bacterial species. When evaluated against Enterobacterales (20 strains), FOS + MIN proved to have additive effect most of the time (65% of isolate), but only in few cases synergistic effect [38]. Similar results were observed when it was tested against multidrug-resistant *P. aeruginosa* [38] and *A. baumannii* isolates; furthermore, in the last case, complete restoration of susceptibility of MIN was reported [65]. Only one study evaluated FOS + TEC combination against Enterobacterales (100 isolates), observing indifference in almost 100% of cases [66]. 2 studies evaluated FOS + MIN combination against vancomycin-resistant *E. faecium* or *E. faecalis* (51 strains), reporting most often indifferent effect and some sporadic case of synergism [13,67]. Otherwise, FOS + DOX combination was tested once against 24 isolates of vancomycin-resistant *E. faecium*, demonstrating to have synergistic or additive effect in most of cases [68]. Finally, when FOS + MIN was tested against MRSA (152, strains, 3 studies) proved to have synergistic effect in numerous cases [18,69,70]. No study reported any case of antagonism.

The combination of minocycline + FOS against *A. baumannii* appears interesting.

### 3.10. Polymyxins

Thirty-two papers evaluating FOS in combination with polymyxins were reviewed (Table 10). Polymyxins are bactericidal drugs that bind to lipopolysaccharide (LPS) and phospholipids in the outer cell membrane of Gram-negative bacteria and leads to disruption of this. Twenty-eight papers evaluated colistin. Colistin breakpoints are ≤ 2 µg/mL for Enterobacterales, *Acinetobacter* spp. and *Pseudomonas* spp. according to the EUCAST [10]. Synergism rates were not unanimous on all studies but was reported in 23/29 papers. Synergisms rate were 100% in 2 in vitro studies against *K. pneumoniae* [50,71] and 2 in vivo studies respectively against *A. baumannii* and *E.coli* [72,73]. The overall effect was indifferent on most isolates of *P. aeruginosa* and Enterobacterales. Antagonism was reported in vitro against *K. pneumoniae* and *A. baumannii*. In particular the combination was antagonist in 100% of all *K. pneumoniae* OXA-48 isolates according to Evren et al. [74].

Four papers evaluated polymyxin B. Polymyxin B breakpoints for Enterobacterales, *Acinetobacter* spp. and *Pseudomonas* spp. are ≤ 2 µg/mL according to CLSI. Synergism was observed in 100% of in vitro isolates of CP *K. pneumoniae* according to Bulman et al. [75]. FOS + polymyxin had a prevalent addictive effect in vitro against *Pseudomonas* spp. [76] and *A. baumannii* [65]. In a study there was a complete polymyxin B susceptibility restoration [65]. No antagonistic effect was observed either in in vitro or in vivo studies.

The combination of polymyxins and FOS appears a good option against Enterobacterales and *P. aeruginosa* strains.

### 3.11. Daptomycin

Thirteen papers evaluating FOS in combination with daptomycin (DAP) were reviewed (Table 11). DAP is a cyclic lipopeptide administered intravenously for Gram-positive infections, acting through bacterial membrane depolarization [77]. Its breakpoints are ≤1 µg/mL for *Staphylococcus* spp. and ≤2 µg/mL for *Enterococcus* spp. [10,78].

When evaluated against *S. aureus* isolates, the combination FOS + DAP had a synergistic effect in vitro against 37–100% of isolates (synergistic effect of the combination against 100% of the tested isolates was reported in 4 in vitro studies [63,79,80,81] and 2 in vivo studies [37,79]). DAP showed excellent synergistic activity in association with FOS against *Enterococcus* spp., resulting in synergistic effect in all 34 tested isolates (4 studies). FOS + DAP also exhibited a greater efficacy against *E. faecalis* biofilm formation than FOS or DAP alone. Efficacy in vivo sometimes differed from the results obtained in vitro, resulting in greater [37] or less [82] efficacy. No antagonistic effect was observed either in in vitro or in vivo studies.

The combination of daptomycin + FOS has good synergistic effect rates against *S. aureus* and *Enterococcus* spp. and deserves clinical interest.

### 3.12. Tigecycline

Fourteen papers evaluating FOS in combination with TIG were reviewed (Table 12). TIG is the first glycylcycline antibiotic, a broad-spectrum class of bacteriostatic derivate from tetracyclines, that acts binding the 30S ribosomal subunits, inhibiting bacterial proteins synthesis. It is only available for intravenous administration and shows activity against either Gram-positive or Gram-negative or atypical bacteria [64]. Its breakpoint are ≤0.5 mg/L both for *S. aureus* and Enterobacterales and ≤0.25 mg/L for *Enterococcus* spp. [10].

When evaluated in vitro against Enterobacterales or *A. baumannii* (10 studies, 338 isolates) FOS + TIG had synergistic effect approximately in 17% of cases and additive effect in the 43%, while indifference was reported for all remaining cases [38,73,74,83,84,85,86,87,88,89]. Furthermore, indifferent effect against all isolates was observed in one in vivo experiment against *E. coli* [73]. Mostly indifference was observed also when it was tested against *N. gonorrhoeae* or *P. aeruginosa* [54,86]. When tested against 61 isolates of *Enterococcus* spp. (3 studies) many cases of synergistic effect was reported in vitro (about 40% of cases) [55,90,91] and in vivo against *E. faecalis* [90]. Finally, 2 studies evaluated FOS + TIG combination in vitro against MRSA, but with inconclusive results (total indifference or almost total synergism) [69,90]. In all in vitro studies only 2 cases of antagonism were reported, against *K. pneumoniae* [89].

According to the literature the combination of TIG + FOS appears to be particularly interesting (good synergistic effect rates) against Enterobacterales and *Enterococcus* spp.

### 3.13. Linezolid

Thirteen papers evaluating FOS in combination with linezolid (LZD) were reviewed (Table 13). LZD is a synthetic antibiotic which binds rRNA on both 30S and 50S ribosomal subunits, inhibiting bacterial proteins synthesis [92]. It is used for Gram-positive infections treatment, including MRSA and *E. faecium* vancomycin-resistant (VREF) infections [93]. Its breakpoint is ≤4 µg/mL both for *S. aureus* and *E. faecium*. 

When evaluated against *S. aureus* isolates (9 studies), combination FOS + LZD had a synergistic effect in vitro approximately in 95% of cases (synergistic effect of the combination against 100% of the tested isolates was reported in 6 in vitro studies [36,43,63,94,95]) and even against staphylococcal biofilm cultures [69]; furthermore, the only 2 in vivo studies performed proved FOS + LZD combination to have higher efficacy than FOS or LZD alone [36,95]. One study evaluated the combination on 2 strains of *S. epidermidis* proving synergism on both [43]. Otherwise, in the 4 studies in which it was tested against *E. faecium*, this combination showed in most cases additive effect and only few cases of synergism. In no case was reported synergistic effect against *E. faecalis* (2 studies). No antagonistic effect was observed either in in vitro or in vivo studies.

The good synergistic effects reported make LZD + FOS a promising combination against *staphylococci*.

### 3.14. Rifampin

Fourteen papers evaluating FOS in combinations with rifampin were reviewed (Table 14). Rifampin breakpoints are ≤0.06 µg/mL for *Staphylococcus* spp., *Streptococcus* spp. and ≤0.125 µg/mL for *S. pneumoniae*. Rifampin inhibits bacterial DNA-dependent RNA polymerase with a concentration related effect. It is used for the treatment of intracellular pathogens and it has a broad-spectrum antibacterial activity. Rifampin breakpoints are not defined either by EUCAST or by CLSI for *Acinetobacter* spp., Enterobacterales and *Enterococcus* spp. Based on literature data, susceptibility was defined as a MIC ≤ 1 µg/mL for *Enterococcus* spp. [71]. Rifampin showed synergistic activity in association with FOS against *Enterococcus* spp., resulting in synergistic effect in 20−100% of cases. High activity was reported in vitro and in vivo in a recent paper where FOS + RIFA also exhibited a greater efficacy against *E. faecalis* biofilm formation [90]. When evaluated against *S. aureus* isolates, the combination FOS + rifampin had a synergistic effect in vitro against 34−100% of isolates. Synergistic effect of the combination against 100% of the tested isolates was reported in 3 in vitro studies [43,90,96] and 2 in vivo studies [37,96]. Antagonistic effect was observed only in 33% of isolates in the study by Quentin et al. [35] where the antibiotic combination was antagonist for the isolates susceptible and intermediate to rifampin and indifferent for those resistant. No antagonistic effect was observed in other studies. 

In clinics RIF + FOS should be considered (usually with a third agent) against *S. aureus* sustained infections, especially when biofilm production is likely.

### 3.15. Miscellanea

Two papers evaluating FOS in combination with metronidazole (MTZ) were reviewed (Table S1). MTZ is a bacteriostatic antimicrobial, active on bacteria (mainly anaerobic) and parasites. When evaluated in vitro against *Helicobacter pylori*, combination FOS + MTZ had a prevalent indifferent effect, an additive effect in only 21% of cases and an antagonist effect in 4% [97]. In vivo study showed a significantly decrease mortality and increase cure rates if the animal treated with MTZ + FOS [98]. 

One paper evaluating FOS in combination with spectinomycin (SCM) was reviewed (Table S1). SCM is an aminocyclitol aminoglycoside antibiotic with bacteriostatic activity, used to treat gonorrhea. In vitro study reported that antimicrobial combinations of SMC + FOS no synergistic effect was found [54].

One paper evaluating FOS in combination with sulbactam (SLB) was reviewed (Table S1). SLB is an irreversible β-lactamase inhibitor capable to binding to penicillin-binding proteins and with weak antimicrobial activity. When evaluated in vitro against *A. baumannii* OXA-23, combination FOS + SLB had a synergistic effect in 75% of case, and an indifferent effect in 25% of cases [99].

One paper evaluating FOS in combination with lincomycin (LNM) was reviewed (Table S1). LMN is a protein synthesis inhibitor with activity against gram positive and anaerobic bacteria. When evaluated in vitro against *S. aureus*, combination FOS + LNM had a synergistic effect in 81% of case and an additive effect in 25% of cases [14].

One paper evaluating FOS in combination with nitroxoline (NTX) was reviewed (Table S1). NTX is a urinary antibacterial agent active against susceptible Gram-positive and Gram-negative organisms. In vitro study, NTX was synergistic with FOS in only 12% of cases and in other cases shoed an indifferent effect (88%) [66].

Two papers evaluating FOS in combination with quinupristin/dalfopristin (Synercid) were reviewed (Table S1). Synercid is a protein synthesis inhibitor used to treat infections by staphylococci and by vancomycin-resistant strain. When evaluated in vitro against methicillin resistant or susceptible *Staphyloccoccus* spp., combination FOS + Synercid had a synergistic effect in 100% of case [43,100].

Three papers evaluating FOS in combination with fusidic acid (FSA) were reviewed (Table S1). FSA is a bacteriostatic antibiotic with acts as a bacterial protein synthesis inhibitor. When evaluated in vitro against MRSA, combination FOS + FSA had a various behavior, showing a synergistic effect in 88–100% of case or an indifferent effect in 100% of cases. No antagonism was found [69,101,102].

Four papers evaluating FOS in combination with chloramphenicol (CHL) were reviewed (Table S1). CHL is a synthetic broad-spectrum antimicrobial, mainly bacteriostatic, active on numerous Gram-positive and Gram-negative, aerobic and anaerobic bacteria; it acts binding 50S ribosomal subunit, inhibiting bacterial protein synthesis [103]. Its breakpoint is ≤ 8 mg/L both for *S. aureus* and Enterobacterales [10]. When evaluated in vitro against either Enterobacterales (468 isolates, 4 studies), combination FOS + CHL had synergistic effect approximately in 40% of cases, while additive effect in 35% and indifferent effect in the remaining cases [14,66,104,105]. Furthermore, one study tested this combination against *S. aureus*, with similar results (synergistic effect against 44% of isolates) [14]. No antagonistic effect was observed.

Three papers evaluating FOS in combination with trimethoprim-sulfamethoxazole (TMP-SMX) were reviewed (Table S1). TMP-SMX is a fixed combination of 2 antimicrobials that inhibits bacterial synthesis of tetrahydrofolate, a necessary cofactor for bacterial DNA synthesis. It is available in oral or intravenous preparation and it is mainly used for treatment of urinary and respiratory infections [106]. Its breakpoint is ≤ 2 µg/mL both *S. aureus* and Enterobacterales [10]. When evaluated in vitro against either *S. aureus* (148 isolates) or Enterobacterales (120 isolates), combination FOS + TMP-SMX had indifferent effect approximately against 92% of isolates [12,38,66]. Only in few cases, against Enterobacterales, was reported synergistic or additive effect (1 study) [38] and even antagonistic effect was reported in 4 cases when tested against *S. aureus* [12].

Two papers evaluating FOS in combination with nitrofurantoin (NTF) were reviewed (Table S1). NTF is a synthetic antibiotic administered orally mainly for treatment of lower urinary tract infections. Its breakpoint is ≤ 64 µg/mL both *E. faecalis* and Enterobacterales [10]. When evaluated in vitro against either vancomycin-resistant *E. faecium* (32 isolates) or Enterobacterales (100 isolates), combination FOS + NTF had indifferent effect against 100% of isolates [66,67]. No synergistic, additive or antagonistic effect was observed.

### 3.16. Non-Antibiotic Molecules

One paper evaluating FOS in combination with auranofin (AF) was reviewed (Table S2). AF is an orally active gold compound for the treatment of rheumatoid arthritis. When evaluated in vitro against *Staphyloccoccus* spp., combination FOS + AF had showed a reduction of bacterial load for both MSSA and MRSA strains. In vivo, this combination had showed a synergistically inhibition of abscess and inflammation formation. No interactions were showed against *S. epidermidis* MS [107]. Three paper evaluating FOS in combination with dilipid ultrashort cationic lipopeptides, tobramycin-efflux pump inhibitor (TOB-EPI) conjugates or amphiphilic lysine-tobramycin conjugates (ALT) against *P. aeruginosa*, were reviewed (Table S2). For all combinations, in vitro studies had showed a synergistic effect (100%). Furthermore, in presence of TOB-EPI or ALT conjugates MICs of FOS were dramatically reduced [108,109,110]. One paper evaluating FOS in combination with β-chloro-L-alanine (β-CLA) was reviewed (Table S2). β-CLA is an amino acid analog of FOS. When evaluated in vitro against MRSA, combination FOS + β-CLA had showed a synergistic effect on biofilm production [111]. One paper evaluating FOS in combination with plectasin NZ2114, compound capable to inhibits a cell wall biosynthesis, was reviewed (Table S2). When plectasin NZ2114 evaluated in vitro against *E. faecalis*, in combination with FOS it no show a synergistic effect [112]. One paper evaluating FOS in combination with 2 quinolone derivatives (A and B) was reviewed (Table S2). When evaluated in vitro against *E. faecalis* VRE and MRSA, combination FOS + A had always showed a synergistic effect, while FOS + B had showed a synergistic effect in 64% of cases and in other cases shoed an additive effect (36%) [113]. One paper evaluating FOS in combination with N-acetylcysteine (NAC), a mucolytic agent, was reviewed (Table S2). The in vitro analysis against *E. coli*, had showed a capable of NAC to reduce biofilm if used in combination with FOS. The most effective combination was that obtained using FOS at 2000 mg/L and NAC at 2 mg/mL [114]. One paper evaluating FOS in combination with sophoraflavanone G (SFG), a phytoalexins, was reviewed (Table S2). When evaluated in vitro against MRSA, combination FOS + SFG had showed a synergistic effect (100%) [115]. One paper evaluating FOS in combination with arenaemycin (ARM), also called pentalenolactones, was reviewed (Table S2). When evaluated in vitro against *P. vulgaris* and *S. gallinarum*, combination FOS + ARM had showed a synergistic effect (100%) [116]. One paper evaluating FOS in combination with chlorogenic acid (CHA) and caffeic acid (CFA) was reviewed (Table S2). When evaluated in vitro against *Resistant Listeria monocytogenes*, combination FOS + CHA had showed a reduction in the cell growth equal to 98% and FOS + CFA as to 85,2%. Moreover, CHA restored a FOS susceptibility in 100%, if 3 mg/L [117]. One paper evaluating FOS in combination with silver (AgNPs) and zinc oxide (ZnONPs) nanoparticles, are molecules known to affect bacterial membranes, was reviewed (Table S2). When evaluated in vitro against *S. aureus*, *S. enterica*, and *E. coli*, combination FOS + AgNPs or ZnONPs had showed a synergistic effect (100%) [118].

## 4. Discussion

FOS is an inhibitor of bacterial wall synthesis with a unique mechanism of action. Its use in clinic is increasing as is often active against MDR bacteria. Intravenous FOS is often administered in combination with other antibiotics therefore the knowledge of pharmacodynamic interactions is of fundamental importance. In this review, we have investigated the role of FOS as partner drug, by analyzing literature studies in which it has been used in vitro and in vivo in combination with other antibiotics and evaluating the antimicrobial activity of combinations against the most common bacterial pathogens. From this huge data collection, no clinically significant antagonistic effect came out between FOS and any most common used antibiotics for the treatment of nosocomial infections.

FOS has been studied in combination with the major antibiotic classes (penicillins, cephalosporins, carbapenems, monobactams, quinolones, aminoglycosides, macrolides, glycopeptides, tetracyclines, polimyxins, lipopeptides, oxazolydinones, and rifampicin) against both Gram-negative and Gram-positive bacteria. A total of 185 literature reports accounted for 9,927 study isolates. FOS-based synergistic interactions were detected in 33.7% of total isolates, although additive and indifferent interactions were more prevalent (65.4%). Antagonism occurred sporadically (0.9% of total isolates).

Clinically significant synergistic interactions were mostly distributed in combination with penicillins (51%), carbapenems (43%), chloramphenicol (39%), and cephalosporins (33%) in *Enterobactaerales*; with linezolid (74%), tetracyclines (72%), and daptomycin (56%) in *S. aureus*; with chloramphenicol (53%), aminoglycosides (43%) and cephalosporins (36%) against *P. aeruginosa*; with daptomycin (97%) in *Enterococcus* spp. and with sulbactam (75%) and penicillins (60%) and in *Acinetobacter* spp.

Notably, 31.2% of synergistic interactions occurred in Enterobacterales (FOS in combination with 3 different antibiotics), followed by 31% occurred in *S. aureus* (FOS in combination with 4 different antibiotics) and 7.6% occurred *Enterococcus* spp. (FOS in combination with 5 different antibiotics). 

From a clinical point of view, taking into account the antimicrobial stewardship principles and the priorities in terms of MDR impact, our work points out good pharmacodynamic interactions rates (additive/synergistic effects) when FOS is especially combined with:

(1) Cephalosporins and cephalosporins + β-lactamase inhibitors, including ceftazidime/avibactam and ceftolozane/tazobactam, for Enterobacterales and *P. aeruginosa*;
(2) carbapenems for *K. pneumoniae* and *P. aeruginosa*;
(3) quinolones for *P. aeruginosa*;
(4) polymyxins for *K. pneumoniae*;
(5) daptomycin for *Staphylococcus* spp (MRSA included), and *Enterococcus* spp.;
(6) linezolid for *Staphylococcus* spp.; and
(7) sulbactam for *A. baumannii*.

When FOS is combined with molecules other than antibiotics, chlorogenic acid and caffeic acid appeared to be good partner drugs against *L. monocytogenes*.

Our tables (including the summarizing Table 15) could act as a useful consultation tool for clinicians using FOS both as empirical or targeted antibiotic regimen.

## 5. Conclusions

In conclusion, taken together, these data, the pharmacological characteristics (i.e., excellent distribution in body sites, the safety and tolerability profile) and the encouraging positive clinical outcome of treated patients highlight the role of FOS as partner drug (mostly intravenously) for the treatment of infections caused by common (including MDR) pathogens. In particular, the presence of synergistic interactions and the almost total absence of antagonisms, make FOS a good partner drug in clinical practice. Moreover, improving FOS-based combinations could act as a meropenem- and colistin-sparing agent, mostly contributing to prevent AMR, especially related to last resource antibiotics.

**Table 1:** Studies on combination between fosfomycin and penicillins, penicillins + β-lactamase inhibitors, penicillinase-resistant penicillins. CB: checkerboard assay; TK: time–kill assay; ET: E-test.

Strain	Year and Country	Author	Penicillin	Number of Isolates	Known Resistance Mechanisms or Determinants (%)	Fosfomycin-Resistant (%)	penicillin-Resistant (%)	In Vitro (methods)/In Vivo (Animal and Site of Infection)	Synergistic Effect (%)	Additive Effect (%)	Indifferent Effect (%)	Antagonistic Effect (%)	Fosfomycin Susceptibility Restoration (%)	Penicillin Susceptibility Restoration (%)	Comments	Reference
Enterobacterales	2019, USA	Avery	piperacillin/tazobactam	49	8 *E. coli*: KPC (25%), NDM (75%), ESBL (62.5%); 35 *Klebsiella* spp: KPC (45.7%), NDM (40%); OXA (14.3%), VIM (8.6%), ESBL (88.6%), fosA (44%); 2 *Citrobacter* spp: KPC (50%), NDM (50%), ESBL (50%), 4 *E. cloacae*: KPC (75%), NDM (25%), ESBL (75%)	20 (40.8%)	49 (100%)	in vitro (ET)	1 (2%)	2 (4%)	46 (94%)	0%	-	-	Data on synergism reported without distinction for bacterial strains. % of FOS-R isolates estimated on the basis of the reported MIC50.	[11]
	2019, USA	Flamm	piperacillin/tazobactam	20	-	-	-	in vitro (CB, TK)	12 (60%)	7 (35%)	0%	0%	-	-	For 1 isolate the efficacy of FOS + PIP/TAZ remained indeterminate.	[38]
	1978, Spain	Olay	ampicillin, carbenicillin	Ampicillin: 17 *E. coli*, 11 *Klebsiella* spp., 7 *E. cloacae*, 14 *Proteus* spp., 22 *Salmonella* spp. Carbenicillin: 16 *E. coli*, 32 *S. marcescens*, 26 *Proteus* spp.	-	-	-	in vitro (CB)	ampicillin: 31 (43%); carbenicillin: 24 (32%)	ampicillin: 31 (43%); carbenicillin: 31 (41%)	ampicillin: 9 (12%); carbenicillin: 19 (25%)	0%	-	-	-	[14]
*E. coli*	2020, Korea	Seok	piperacillin/tazobactam	2	ESBL (100%)	0%	1 (50%)	in vitro (TK)	0%	0%	2 (100%)	0%	-	-	-	[119]
	2018, France	Berleur	temocillin	3	KPC (33.3%), OXA (33.3%)	0%	Breakpoints NA	in vitro (CB, TK); in vivo (mouse, peritonitis)	0%	in vitro: 3 (100%); in vivo: 3 (100%)	0%	0%	-	-	-	[15]
	2014, Sweden	Hickam	mecillinam	2	ESBL, OXA (50%)	0%	0%	in vitro (CB, TK)	2 (100%)	0%	0%	0%	-	-	-	[120]
	1977, Poland	Borowski	ampicillin	10	-	-	-	in vitro (CB)	7 (70%)	1 (10%)	2 (20%)	0%	-	-	-	[121]
*K. pneumoniae*	2014, Sweden	Hickam	mecillinam	1	ESBL, OXA (100%)	0%	0%	in vitro (CB, TK)	1 (100%)	0%	0%	0%	-	-	-	[120]
*Salmonella* spp.	1977, Spain	Perea	ampicillin	90	-	17 (18.9%)	11 (12%)	in vitro (CB, TK)	74 (82%)	7 (7%)	7 (7%)	0%	-	-	For 2 isolates the effect of FOS + ampicillin remained indeterminate. The authors considered synergistic the effect for FICI up to 0.75.	[104]
	1977, Spain	Figueroa	ampicillin	16	-	-	-	in vitro (CB)	15 (93%)	1 (6%)	0%	0%	-	-	*S. typhi*. The authors considered synergistic the effect for FICI up to < 1. They also evaluated different antibiotic combinations on patients with typhoid fever: FOS + AMP resulted in the highest rate of cures.	[105]
*Shigella* spp.	1977, Spain	Perea	ampicillin	50	-	27 (54%)	30 (60%)	in vitro (CB, TK)	27 (54%)	9 (18%)	14 (28%)	0%	-	-	The authors considered synergistic the effect for FICI up to 0.75.	[104]
*P. aeruginosa*	2019, USA	Avery	piperacillin/tazobactam	103	-	NA (at least 71)	103 (100%)	in vitro (ET)	3 (2%)	26 (25%)	74 (71%)	0%	-	15 (14.6%)	-	[33]
	2019, USA	Flamm	piperacillin/tazobactam	5	-	-	-	in vitro (CB, TK)	0%	5 (100%)	0%	0%	-	-	-	[38]
	2013, Brazil	dos Santos	piperacillin/tazobactam	4	-	4 (100%)	2 (50%)	in vitro (CB)	4 (100%)	0%	0%	0%	2 (50%)	1 (50%)	-	[48]
	2002, Japan	Okazaki	piperacillin	30	-	15 (50%)	30 (100%)	in vitro (efficacy time index)	3 (10%)	6 (20%)	21 (70%)	0%	0%	15 (50%)	-	[39]
	1984, Japan	Takahashi	piperacillin	20	-	-	-	in vitro (CB)	4 (20%)	16 (80%)	0%	0%	-	-	-	[122]
	1978, Spain	Olay	carbenicillin	in vitro: 73; in vivo: 2	-	-	-	in vitro (CB); in vivo (mouse, peritonitis)	in vitro: 21 (28%); in vivo: 2 (100%)	in vitro: 40 (54%)	in vitro: 12 (16%)	0%	-	-	-	[14]
*Acinetobacter* spp.	2019, USA	Flamm	piperacillin/tazobactam	5 (*A. baumannii-calcoaceticus* species complex)	-	-	-	in vitro (CB, TK)	3 (60%)	1 (20%)	0%	0%	-	-	For 1 isolate the efficacy of FOS + PIP/TAZ remained indeterminate.	[38]
*S. aureus*	2015, Spain	del Río	amoxicillin + clavulanic acid	10	Methicillin-resistant *Staphylococcus aureus* (MRSA) (100%)	1 (10%)	10 (100%)	in vitro (TK)	in vitro: 8 (80%); in vivo: 2 (100%)	in vitro: 2 (20%)	0%	0%	-	-	-	[28]
	2003, Japan	Nakazawa	ampicillin	32	MRSA (100%)	29 (91%)	31 (96%)	in vitro (efficacy time index)	4 (12%)	2 (6%)	26 (81%)	0%	-	-	-	[18]
	1997, Italy	Ferrara	oxacillin	16	MRSA (100%)	NA (at least 8)	16 (100%)	in vitro (TK)	3 (18%)	3 (18%)	4 (25%)	-	-	-	Addition or indifference was observed for the remaining 6 strains (data not shown).	[123]
	1994, Japan	Komatsuzawa	oxacillin	38	MRSA (60.5%)	33 (86.8%)	23 (60%)	in vitro (CB)	20 (52%)	17 (44%)	1 (2%)	0%	-	-	-	[124]
	1985, USA	Alvarez	methicillin	148	MRSA (100%)	NA (< 15)	148 (100%)	in vitro (CB)	69 (46%)	-	-	1 (1%)	-	-	For the 78 remaining strains it was not specified if the combination FOS + methicillin acted with an additive or indifferent effect.	[12]
	1978, Spain	Olay	ampicillin, carbenicillin	ampicillin: 27; carbenicillin: 28	-	-	-	in vitro (CB)	ampicillin: 15 (55%); carbenicillin: 10 (35.7%)	ampicillin: 9 (33%); carbenicillin: 18 (64%)	ampicillin: 3 (11%); carbenicillin: 0%	0%	-	-	-	[14]
	1977, Poland	Borowski	penicillin G	11	-	-	-	in vitro (CB)	5 (45%)	2 (18%)	4 (36%)	0%	-	-	-	[121]
*S. epidermidis*	1997, Italy	Ferrara	oxacillin	12	MRSE (100%)	NA (at least 6)	12 (100%)	in vitro (TK)	6 (50%)	1 (8%)	1 (8%)	-	-	-	Data of the other 4 strains are not shown.	[123]
*Streptococcus* spp.	2017, Germany	Gonzalez Moreno	benzylpenicillin	3	-	1 (33.3%)	0%	in vitro (microcalorimetry for biofilms)	0%	0%	3 (100%)	0%	-	-	*S. agalactiae*, *S. pyogenes*, *S. oralis*. High-dose FOS caused a delay of 8 h in the production of heat, compared with untreated controls, suggesting that the treatment could result in a reduction in the number of viable sessile cells, although not in complete biofilm eradication.	[9]
	1981, Spain	Vicente	penicillin G	17	-	9 (53%)	5 (29%)	in vitro (CB, TK); in vivo (rabbit, endocarditis)	in vitro: 4 (23%)	in vitro: 12 (71%); in vivo: 100%	in vitro: 1 (6%)	0%	-	-	*S. sanguis*. The mean log10 CFU per gram of vegetations in the FOS + penicillin groups was significantly lower than that in the FOS groups but was not significantly lower than that in the penicillin group.	[17]
	1978, Spain	Olay	ampicillin	37	-	-	-	in vitro (CB)	12 (32%)	11 (29%)	14 (37%)	0%	-	-	-	[14]
*S. pneumoniae*	2001, Spain	Bañón Arias	penicillin	10	-	1 (10%)	8 (80%)	in vitro (TK)	10 (100%)	0%	0%	0%	-	-	Synergistic effect difficult to determine. It is reported as synergistic against all isolates based on authors’ considerations and on the comparison between cumulative efficacy of MIC + MIC and MIC/4 + MIC/4.	[125]
	1996, France	Chavanet	amoxicillin	1	-	0%	1 (100%)	in vivo (rabbit, fibrin clot infection)	1 (100%)	0%	0%	0%	-	-	-	[23]
	1995, Japan	Kikuchi	benzylpenicillin	51	-	0%	51 (100%)	in vitro (CB, TK)	9 (17%)	42 (82%)	0%	0%	-	-	-	[126]
*Enterococcus* spp.	2013, Taiwan	Tang	ampicillin	10 *E. faecium*, 9 *E. faecalis*	VRE (100%)	13 (68%)	9 (47%)	in vitro (TK, biofilm)	TK: 3 (15%)	-	-	biofilm: 6 (31%)	-	-	The 3 isolates exhibiting synergistic effect were all *E. faecium*. The 6 isolates exhibiting antagonistic effect on biofilm formation were all *E. faecalis*. From the data reported in the paper it was not possible to establish the effect of the combination against the other isolates.	[13]
	1995, France	Pestel	penicillin	10	-	10 (100%)	6 (60%)	in vitro (CB, TK)	6 (60%)	-	-	0%	-	-	*E. faecalis*, *E. faecium*, *E. casseliflavus*, *E. durans*. The authors did not distinguish between additive and indifferent effect.	[127]
*E. faecalis*	2011, Italy	Farina	ampicillin	27	-	2 (7%)	0%	in vitro (ET)	2 (7%)	0%	25 (92%)	0%	-	-	The Authors considered 0.5 < FICI ≤ 4 as indifferent.	[128]
*E. faecium*	2013, USA	Descourouez	amoxicillin	4	VRE (100%)	0%	4 (100%)	in vitro (TK)	100%	0%	0%	0%	-	-	The combination resulted also strongly bactericidal.	[67]

**Table 2:** Studies on combination between fosfomycin and cephalosporins, cephalosporins + β-lactamase inhibitors. CB: checkerboard assay; TK: time–kill assay; ET: E-test.

Strain	Year and Country	Author	Cephalosporin	Number of Isolates	Known Resistance Mechanisms or Determinants (%)	FOS-Resistant (%)	cephalosporin-Resistant (%)	In Vitro (Methods)/In Vivo (Animal and Site of Infection)	Synergistic Effect (%)	Additive Effect (%)	Indifferent Effect (%)	Antagonistic Effect (%)	FOS Susceptibility Restoration (%)	Cephalosporin Susceptibility Restoration (%)	Comments	Reference
Enterobacterales	2019, USA	Avery	cefepime (FEP), ceftolozane/tazobactam (C/T), ceftazidime (CTZ), ceftazidime/avibactam (CZA)	49 (26 tested for CZA)	8 *E. coli*: KPC (25%), NDM (75%), ESBL (62%); 35 *Klebsiella* spp: KPC (45%), NDM (40%); OXA (14%), VIM (8%), ESBL (88%), fosA (44%); 2 *Citrobacter* spp: KPC (50%), NDM (50%), ESBL (50%), 4 *E. cloacae*: KPC (75%), NDM (25%), ESBL (75%)	20 (40%)	49 (100%)	in vitro (ET)	FEP: 2 (4%); C/T: 8 (16%); CTZ: 3 (6%); CZA: 0%	FEP: 5 (10%); C/T: 11 (22%); CTZ: 8 (16.3%); CZA: 3 (11.5%)	FEP: 42 (85%); C/T: 30 (61%); CTZ: 38 (77%); CZA: 23 (88%)	0%	0%	0%	Data on synergism reported without distinction for bacterial strains. % of FOS-R isolates estimated on the basis of the reported MIC50.	[11]
	2019, USA	Flamm	ceftazidime	20	-	-	-	in vitro (CB, TK)	8 (40%)	10 (50%)	0%	0%	-	-	For 2 isolates the efficacy of FOS + CTZ remained indeterminate.	[38]
	1978, Spain	Olay	cephalexin	23 *E. coli*, 29 *Salmonella* spp., 8 *Klebsiella* spp., 11 *E. cloacae*, 16 *S. marcescens*, 16 *Proteus* spp.	-	-	-	in vitro (CB)	42 (40%)	46 (44%)	15 (14%)	0%	-	-	-	[14]
*E. coli*	2020, Korea	Seok	cefixime	4	ESBL (50%)	0%	2 (50%)	in vitro (TK)	4 (100%)	0%	0%	0%	-	-	-	[119]
	2014, France	Lefort	cefoxitim	2	ESBL (50%)	0%	breakpoints NA	in vitro (TK); in vivo (mouse, urinary tract infection)	in vitro: 2 (100%); in vivo: 2 (100%)	0%	0%	0%	-	-	-	[30]
*K. pneumoniae*	2019, Poland	Ojdana	ceftazidime-avibactam	19	NDM (52%); KPC (42%); OXA (5%)	10 (53%)	10 (53%)	in vitro (ET)	9 (47%)	7 (36%)	3 (15%)	0%	-	-	-	[31]
	2019, USA	Mikhail	ceftazidime-avibactam	21	fosA/fosA-like, KPC, ESBL, OXA (100%)	15 (71%)	0%	in vitro (CB, TK)	10 (47%)	9 (42%)	2 (9%)	0%	-	0% (all S)	It is reported only the reduction of CZA in combination and time–kill was performed only on 2 isolates randomly selected, therefore a reduction of at least 4 times was considered as synergistic. A 2-fold reduction was considered as additive. No reduction was considered as indifferent. In increase of MIC in combination was considered antagonistic.	[21]
	1977, Spain	Daza	cephapirin	33	-	100%	breakpoints NA	in vitro (CB)	1 (3%)	-	-	-	0%	Breakpoints NA (reduction of MIC from 16 to 4 µg/mL)	The authors reported only the number of isolates on which the combination had a synergistic effect.	[66]
*P. aeruginosa*	2020, Brazil	Cuba	ceftolozane/tazobactam	27	carbapenemase-producing (74%)	26 (96%)	22 (81%)	in vitro (ET, TK)	24 (88%)	3 (11%)	0%	0%	24 (92%)	-	It is not possible to establish the % of strains with FOS susceptibility restoration because the MIC for all R strains was > 64 ug/mL and it is not reported the MIC in combination but the MIC fold reduction. It is however strongly reduced (range: 2–16 fold reduction).	[32]
	2020, USA	Mullane	cefepime, ceftolozane/tazobactam	28 CEF; 15 C/T	-	-	-	in vitro (CB, TK)	CEF: 5 (18%); C/T: 5 (33%)	CEF: 20 (71%); C/T: 8 (53%)	CEF: 3 (11%); C/T: 2 (14%)	0%	-	CEF: 1 (4%); C/T: 5 (33%)	-	[129]
	2019, USA	Mikhail	ceftazidime-avibactam	21	fosA/fosA-like, KPC, ESBL, OXA (100% at least 1 resistance gene)	19 (90%)	5 (23%)	in vitro (CB, TK)	7 (33%)	6 (28%)	8 (38%)	0%	-	1 (20%)	It is reported only the reduction of CZA in combination and time–kill was performed only on 2 isolates randomly selected, therefore a reduction of at least 4 times was considered as synergistic. A 2-fold reduction was considered as additive. No reduction was considered as indifferent. In increase of MIC in combination was considered antagonistic.	[21]
	2019, USA	Papp-Wallace	ceftazidime-avibactam	1	-	0%	1 (100%)	in vitro (CB, TK); in vivo (mouse)	in vitro: 100%; in vivo: 100%	0%	0%	0%	-	-	-	[29]
	2019, USA	Avery	cefepime (FEP), ceftolozane/tazobactam (C/T), ceftazidime (CAZ), ceftazidime/avibactam (CZA)	92 FEP, 14 C/T, 81 CAZ, 16 CZA	Carbapenem-resistant (100%)	-	100%	in vitro (ET)	FEP: 22 (23%); C/T: 7 (50%); CAZ: 42 (51%); CZA: 4 (25%)	FEP: 53 (57%); C/T: 5 (35%); CAZ: 31 (38%); CZA: 12 (75%)	FEP: 17 (18%); C/T: 2 (14%); CAZ: 8 (9%); CZA: 0%	0%	-	FEP: 56 (60%); C/T: 10 (71%); CAZ: 46 (56%); CZA: 11 (68%)	-	[33]
	2019, USA	Flamm	ceftazidime	5	-	-	-	in vitro (CB, TK)	2 (40%)	3 (60%)	0%	0%	-	-	-	[38]
	2018, USA	Monogue	ceftolozane/tazobactam	4	-	3 (75%)	2 (50%)	in vitro (TK)	1 (25%)	2 (50%)	1 (25%)	0%	-	-	-	[34]
	2013, Brazil	dos Santos	ceftazidime	3	-	3 (100%)	3 (100%)	in vitro (CB)	3 (100%)	0%	0%	0%	1 (33%)	2 (66%)	-	[48]
	2005, Thailand	Pruekprasert	ceftazidime	18	-	-	-	in vitro (CB)	2 (11%)	6 (33%)	6 (33%)	4 (22%)	-	-	-	[22]
	2002, Japan	Okazaki	ceftazidime, cefepime	30	-	15 (50%)	CAZ: 28 (93%), CEFP: 26 (86.7%)	in vitro (efficacy time index)	CAZ: 21 (70%); CEFP: 24 (80%)	CAZ: 8 (26%); CEFP: 1 (3.3%)	CAZ: 1 (3%); CEFP: 5 (16%)	0%	CAZ: 3 (20%); CEFP: 6 (40%)	CAZ: 19 (67%); CEFP: 26 (100%)	-	[39]
	1999, Japan	Hayami	ceftazidime	26	-	NA (at least 13)	NA (at least 5)	in vitro (CB, TK)	7 (26%)	14 (53%)	5 (19%)	0%	-	-	-	[130]
	1997, France	Tessier	ceftazidime	40	-	21 (52%)	14 (35%)	in vitro (CB)	0%	8 (20%)	32 (80%)	0%	20 (95%)	8 (57%)	Although the combination had a synergistic effect on no tested strains, it is of clinical relevance as it restored FOS and CTZ susceptibility in many resistant isolates.	[131]
	1984, Japan	Takahashi	cefoperazone, cefsulodin	20 (cefoperazone), 23 (cefsulodin)	-	-	-	in vitro (CB)	cefoper: 17 (85%); cefsul: 19 (92%)	cefoper: 3 (15%); cefsul: 4 (17%)	0%	0%	-	-	-	[122]
*A. baumannii*	2019, USA	Flamm	ceftazidime	5 (*A. baumannii*-calcoaceticus species complex)	-	-	-	in vitro (CB, TK)	2 (40%)	1 (20%)	1 (20%)	0%	-	-	For 1 isolate the efficacy of FOS + CTZ remained indeterminate.	[38]
	1996, Spain	Martinez-Martinez	ceftazidime	34	-	34 (100%)	32 (94%)	in vitro (CB)	1 (3%)	NA	NA	0%	-	-	Only synergistic and antagonistic effect reported.	[132]
*Staphylococcus* spp.	1995, Italy	Marchese	cefdinir	6 *S. aureus*, 8 *S. epidermidis*, 2 *S. hominis*, 2 *S. xylosus*, 5 *S. saprophyticus*, 2 *S. haemolyticus*	Penicillin-resistant (100%)	-	-	in vitro (CB, TK)	4 (16%)	-	-	0%	-	-	The authors considered 0.5 < FICI ≤ 4 as indifferent, therefore it is not possible to establish if the effect was additive or indifferent for most strains.	[114]
*S. aureus*	2003, Japan	Nakazawa	flomoxef sodium (FS), cefmetazole (CEM), cefotiam (CET), cefoperazone/sulbactam (CS)	32	MRSA (100%)	29 (91%)	FS: 29 (91%); CEM: 16 (50%); CET: 30 (94%); CS: 27 (84%)	in vitro (efficacy time index)	FS: 7 (22%); CEM: 26 (81%); CET: 7 (22%); CS: 19 (59%)	FS: 11 (34%); CEM: 3 (9%); CET: 1 (3%); CS: 8 (25%)	FS: 14 (44%); CEM: 3 (9%); CET: 22 (69%); CS: 5 (15%)	0%	-	-	-	[18]
	1978, Spain	Olay	cephalexin	24	-	-	-	in vitro (CB)	17 (70.8%)	7 (29.2%)	0%	0%	-	-	-	[14]
	2015, Spain	del Río	ceftriaxone	in vitro 10; in vivo 2	MRSA (100%)	1 (10%)	10 (100%)	in vitro (TK); in vivo (rabbit, endocarditis)	in vitro: 8 (80%); in vivo: 2 (100%)	in vitro: 2 (20%)	0%	0%	-	-	% of sterile vegetations: FOS alone 0%, IMI alone 0%, FOS + CRO 62%.	[28]
	1985, Germany	Portier	cefotaxime, cephalotin, cefoperazone, cefamandole	10	MRSA (100%)	0%	10 (100%)	in vitro (CB)	cefotaxime, cephalotin, cefoperazone, cefamandole: 10 (100%)	0%	0%	0%	-	-	-	[20]
	1990, France	Chavanet	cefotaxime	1	MGRSA (100%)	0%	1 (100%)	in vivo (rabbit, subcutaneous fibrin clots)	1 (100%)	0%	0%	0%	-	-	Synergistic effect was observed when both drugs were administered in two divided doses.	[27]
	1985, France	Kazmierczak	cefotaxime	1	-	0%	1 (100%)	in vivo (rabbit, meningitis)	0%	1 (100%)	0%	0%	-	-	Cefotaxime: variable drop in bacterial numbers from one rabbit to another during the first 12 h, then a bacteriostasis. FOS: rapid bactericidal effect during the first 12 h, becoming slower during the following 36 h (0.03% surviving bacteria at 48 h). Cefotaxime + FOS: rapid bactericidal effect remaining steady over the 48-h period (0.001% surviving bacteria at 48 h).	[26]
	1991, Japan	Matsuda	cefmetazole	25	MRSA (100%)	25 (100%)	25 (100%)	in vitro (CB, TK)	11 (44%)	11 (44%)	3 (12%)	0%	-	-	-	[133]
	1986, Japan	Utsui	cefmetazole	14 in vitro, 7 in vivo	MRSA (100%)	-	14 (100%)	in vitro (CB, TK); in vivo (mouse)	in vitro: 10 (71%); in vivo: 5 (71%)	in vitro: 4 (28%); in vivo: 2 (28%)	0%	0%	-	-	-	[25]
	1987, France	Courcol	ceftriaxone	6	-	1 (16.%)	6 (100%)	in vitro (CB, TK)	CB: 1 (16%); TK: 1 (16%)	CB: 0%; TK: -	CB: 4 (66%); TK: 3 (50%)	CB: 1 (16%); TK: -	-	-	Different activity of the drug combination with checkerboard assay or time–kill assay. The effect of FOS + ceftriaxone on 2 isolates remained indeterminate. The authors considered the combination antagonistic when the FICI was > 2.	[19]
	1985, USA	Alvarez	cefamandole	148	MRSA (100%)	NA (<15)	-	in vitro (CB)	97 (66%)	-	-	0%	-	-	For the 78 remaining isolates it was not specified if the combination FOS + cefamandole acted with an additive or indifferent effect.	[12]
	2001, Austria	Grif	cefazolin	5	MRSA (20%), GISA (20%)	-	-	in vitro (CB, TK)	5 (100%)	0%	0%	0%	-	-	-	[43]
*S. epidermidis*	2001, Austria	Grif	cefazolin	2	-	-	-	in vitro (CB, TK)	0%	0%	2 (100%)	0%	-	-	-	[43]
	1987, France	Courcol	ceftriaxone	6	-	2 (33.3%)	6 (100%)	in vitro (CB, TK)	CB: 1 (16%); TK: 5 (83.3%)	CB: 0%; TK: -	CB: 5 (83%); TK: -	CB: 1 (16%); TK: -	-	-	Different activity of the drug combination with checkerboard assay or time–kill assay. The effect of FOS + ceftriaxone on 1 isolate remained indeterminate. The authors considered the combination antagonistic when the FICI was > 2.	[19]
*S. pneumoniae*	2006, Spain	Ribes	ceftriaxone	2	-	0%	2 (100%)	in vitro (TK); in vivo (rabbit, meningitis)	0%	in vitro: 1 (50%); in vivo: 2 (100%)	in vitro: 1 (50%)	0%	-	-	-	[24]
	2001, Spain	Bañón Arias	ceftriaxone	10	-	1 (10%)	7 (70%)	in vitro (TK)	10 (100%)	0%	0%	0%	-	-	Synergistic effect difficult to determine. It is reported as synergistic against all isolates based on authors’ considerations and on the comparison between cumulative efficacy of MIC + MIC and MIC/4 + MIC/4.	[125]
	1994, France	Doit	ceftriaxone	26	-	0%	20 (76%)	in vitro (TK)	0%	26 (100%)	0%	0%	-	-	-	[134]
	1993, France	Barakett	cefotaxime	7	-	0%	2 (28%)	in vitro (TK)	3 (42%)	1 (14%)	3 (42%)	0%	-	-	-	[135]
	1995, France	Chavanet	cefotaxime, ceftriaxone	1	-	0%	1 (100%)	in vitro (TK); in vivo (rabbit, fibrin clot infection)	in vitro: 0%; in vivo: 1 (100%, cefotaxime)	in vitro: 1 (100%, both cefotaxime and ceftriaxone); in vivo: 1 (100%, ceftriaxone)	0%	0%	-	-	-	[23]
*S. sanguis*	1981, Spain	Vicente	cefoxitim	17	-	9 (53%)	3 (16%)	in vitro (CB, TK); in vivo (rabbit, endocarditis)	in vitro: 8 (47%); in vivo: 100%	in vitro: 8 (47%)	in vitro: 1 (6%)	0%	-	-	The mean log10 CFU per gram of vegetations in the FOS + cefoxitim groups was significantly lower than that in the FOS groups and in the cefoxitim groups.	[17]
*Enterococcus* spp.	1995, France	Pestel	cefotaxime	50	-	48 (96%)	50 (100%)	in vitro (CB, TK)	45 (90%)	-	5 (10%)	0%	-	-	*E. faecalis*, *E. faecium*, *E. casseliflavus*, *E. durans*. The authors did not distinguish between additive and indifferent effect.	[127]
*E. faecalis*	2011, Italy	Farina	ceftriaxone	27	-	2 (7%)	27 (100%)	in vitro (ET)	15 (55%)	0%	12 (44%)	0%	-	-	The authors did not distinguish between additive and indifferent effect, considering 0.5 < FICI ≤ 4 as indifferent.	[128]
*N. gonorrhoeae*	2015, Switzerland	Hauser	ceftriaxone	8	-	0%	1 (12.5%)	in vitro (CB)	0%	0%	8 (100%)	0%	-	-	-	[57]
	2015, The Netherlands	Wind	cefixime, ceftriaxone	4	-	-	-	in vitro (ET)	0%	cefixime: 1 (25%); ceftriaxone: 2 (50%)	cefixime: 3 (75%); ceftriaxone: 2 (50%)	0%	-	-	-	[54]
	2014, USA	Barbee	cefixime, ceftriaxone	32	-	0%	cefotaxime: 29 (90%), cefixime: 6 (18%), ceftriaxone: 0%	in vitro (ET)	0%	0%	32 (100%)	0%	-	-	The authors did not distinguish between additive and indifferent effect, considering 0.5 < FICI ≤ 4 as indifferent.	[136]

**Table 3:** Studies on combination between fosfomycin and carbapenems. CB: checkerboard assay; TK: time–kill assay; ET: E-test.

Strains	Year and Country	Author	Carbapenem	Number of Isolates	Known Resistance Mechanisms or Determinants (%)	FOS-Resistant (%)	Carbapenem-Resistant (%)	In Vitro (Methods)/In Vivo (Animal and Site of Infection)	Synergistic Effect (%)	Additive Effect (%)	Indifferent Effect (%)	Antagonistic Effect (%)	FOS Susceptibility Restoration (%)	Carbapenem Susceptibility Restoration (%)	Comments	Reference
Enterobacterales	2019, USA	Avery	meropenem	49	8 *E. coli*: KPC (25%), NDM (75%), ESBL (62%); 35 *Klebsiella* spp: KPC (45%), NDM (40%); OXA (14%), VIM (8%), ESBL (88%), fosA (44%); 2 *Citrobacter* spp: KPC (50%), NDM (50%), ESBL (50%), 4 *E. cloacae*: KPC (75%), NDM (25%), ESBL (75%)	20 (40.8%)	49 (100%)	in vitro (ET)	1 (2%)	10 (20%)	38 (77%)	0%	-	-	Data on synergism reported without distinction for bacterial strains. % of FOS-R isolates estimated on the basis of the reported MIC50.	[11]
	2019, USA	Flamm	meropenem	20	-	-	-	in vitro (CB, TK)	8 (40%)	10 (50%)	0%	0%	-	-	For 2 isolates the efficacy of FOS + meropenem (MER) remained indeterminate.	[38]
*E. coli*	2020, Egypt	El-Wafa	imipenem	8	-	3 (37.5%)	7 (87.5%)	in vitro (CB, TK)	2 (25%)	5 (62%)	0%	0%	2 (66%)	6 (87%)	For 1 isolate the efficacy of FOS + MER remained indeterminate	[42]
	2019, India	Sugathan	meropenem	50	-	0%	8 (16%)	in vitro (TK)	34 (68%)	14 (28%)	2 (4%)	0%	0% (all S)	2 (25%)	-	[137]
	2019, Germany	Loose	meropenem, ertapenem	4	-	1 (25%)	3 (75%)	in vitro (CB)	4 (100%)	0%	0%	0%	-	-	-	[138]
	2013, Austria	Lingscheid	doripenem	10	ESBL (80%), AmpC (20%)	0%	-	in vitro (CB, TK)	8 (80%)	-	.	0%	-	-	The authors reported FICI ranging from 0.5 to 4, without distinction between additive and indifferent effect.	[139]
	2012, Greece	Samonis	imipenem, meropenem, doripenem	20	ESBL (100%)	0%	0%	in vitro (ET)	IMI: 11 (55%); MER: 5 (25%); DORI: 6 (30%)	IMI: 9 (45%); MER: 15 (75%); DOR: 14 (70%)	0%	0%	-	-	-	[86]
	2010, Thailand	Netikul	ertapenem, imipenem, meropenem, doripenem	8	ESBL (87%)	0%	8 (100%)	in vitro (ET)	0%	ERT: 5 (62%); IMI: 2 (25%); MER: 2 (25%); DOR: 1 (12%)	ERT: 3 (37%); IMI: 6 (75%); MER: 6 (75%); DOR: 7 (87%)	0%	-	-	-	[140]
*K. pneumoniae*	2020, India	Bakthavatchalam	meropenem	50	OXA (78%), NDM (32%)	-	50 (100%)	in vitro (TK)	10 (20%)	0%	40 (80%)	0%	-	-	-	[141]
	2020, Turkey	Erturk Sengel	meropenem	17	OXA (70%), NDM (70%)	7 (41%)	17 (100%)	in vitro (CB, TK)	15 (88%)	2 (11%)	0%	0%	4 (23%)	-	-	[142]
	2019, Germany	Loose	meropenem, ertapenem	3	-	3 (100%)	2 (66%)	in vitro (CB)	2 (66%)	1 (33%)	0%	0%	-	-	-	[138]
	2019, Brazil	Perdigão Neto	meropenem	9	ESBL, KPC (100%); OXA (4%), fosA (100%)	9 (100%)	9 (100%)	in vitro (CB, TK)	8 (88%)	0%	1 (11%)	0%	2 (22%)	0%	-	[143]
	2017, Taiwan	Tseng	meropenem	25	see comments	12 (48%)	24 (96%)	in vitro (CB)	25 (100%)	0%	0%	0%	-	-	The 25 isolates were randomly selected among 642 isolates with the following resistance determinants: fosA3 (5.5%), foskp96 (4.2%), KPC (10.1%), IMP (0.8%), VIM (0.2%). It is not reported which carbapenemases and fosfomycinases were present in the 25 isolates tested for synergism.	[144]
	2017, China	Yu	imipenem, ertapenem	136	KPC (100%)	78 (57%)	136 (100%)	in vitro (CB, TK)	IMI: 21 (15%); ERT: 30 (22%)	IMI: 114 (83%); ERT: 104 (76%)	IMI: 1 (1%); ERT: 2 (1%)	0%	-	-	-	[89]
	2016, Brazil	Albiero	meropenem	18	KPC (100%)	13 (72%)	16 (89%)	in vitro (CB)	12 (66%)	3 (16%)	3 (16%)	0%	12 (92.3%)	4 (25%)	-	[145]
	2014, Sweden	Tängdén	meropenem	4	NDM (50%), VIM (50%), ESBL (100%)	2 (50%)	3 (75%)	in vitro (TK)	0%	0%	4 (100%)	0%	-	-	-	[146]
	2013, Turkey	Evren	imipenem, meropenem	12	OXA-48 (100%)	12 (100%)	12 (100%)	in vitro (CB)	IMI: 5 (41%); MER: 4 (33%)	IMI: 6 (50%); MER: 6 (50%)	IMI: 1 (8%); MER: 2 (16%)	0%	-	-	-	[74]
	2013, Austria	Lingscheid	doripenem	5	ESBL (60%), AmpC (100%)	0%	-	in vitro (CB, TK)	5 (100%)	0%	0%	0%	-	-	-	[139]
	2012, Greece	Samonis	imipenem, meropenem, doripenem	64	KPC (78%), ESBL (21%)	1 (1%)	51 (78% )	in vitro (ET)	KPC: IMI: 37 (74%); MER: 35 (70%); DOR: 37 (74%). ESBL: IMI: 11 (78%); MER: 6 (42%); DOR: 6 (42%)	KPC: IMI: 13 (26%); MER: 15 (30%); DOR: 13 (26%). ESBL: IMI: 3 (21%); MER: 8 (57%); DOR: 8 (57%)	0%	0%	-	-	-	[86]
	2011, Greece	Souli	meropenem	17	KPC (100%)	4 (23%)	17 (100%)	in vitro (TK)	11 (64%)	0%	6 (35%)	0%	-	-	-	[53]
	2010, Thailand	Netikul	ertapenem, imipenem, meropenem, doripenem	8	ESBL (87%)	4 (50%)	8 (100%)	in vitro (ET)	0%	ERT: 5 (62%); IMI: 2 (25%); MER: 1 (12%); DOR: 2 (25%)	ERT: 3 (37%); IMI: 6 (75%); MER: 7 (87%); DOR: 6 (75%)	0%	-	-	-	[140]
*E. cloacae*	2019, Germany	Loose	meropenem, ertapenem	2	-	2 (100%)	1 (50%)	in vitro (CB)	0%	2 (100%)	0%	0%	-	-	-	[133]
	2013, Austria	Lingscheid	doripenem	3	1 (33%)	0%	-	in vitro (CB, TK)	1 (33%)	-	-	0%	-	-	The authors reported FICI ranging from 0.5 to 4, without distinction between additive and indifferent effect.	[139]
*P. aeruginosa*	2020, USA	Mullane	meropenem	30	-	14 (47%)	30 (100%)	in vitro (CB, TK)	5 (17%)	9 (30%)	16 (53%)	0%	0%	0%	-	[129]
	2019, USA	Avery	meropenem	153	-	NA (at least 71)	153 (100%)	in vitro (ET)	29 (19%)	55 (35%)	69 (45%)	0%	-	21 (13%)	-	[33]
	2019, Brazil	Albiero	meropenem	19	MBL (52%)	17 (89%)	16 (84%)	in vitro (CB)	15 (88%)	3 (15%)	1 (5%)	0%	15 (88%)	7 (43%)	-	[147]
	2019, USA	Flamm	meropenem	5	-	-	-	in vitro (CB, TK)	1 (20%)	3 (60%)	1 (20%)	0%	-	-	-	[38]
	2019, Brazil	Perdigão Neto	meropenem	1	OXA, fosA (100%)	1 (100%)	1 (100%)	in vitro (CB, TK)	1 (100%)	0%	0%	0%	1 (100%)	1 (100%)	-	[143]
	2018, USA	Drusano	meropenem	1	-	-	-	in vitro (hollow-fiber infection model)	1 (100%)	0%	0%	0%	-	-	Combination therapy was able to counterselect resistance emergence.	[148]
	2017, Spain	Hamou-Segarra	imipenem	4	-	1 (25%)	-	in vitro (TK)	4 (100%)	0%	0%	0%	-	-	FOS and imipenem (IMI) alone lead to bacterial regrowth, while no regrowth was observed with the combination FOS + IMI.	[149]
	2015, Thailand	Kunakonvichaya	imipenem, meropenem, doripenem	70	-	-	70 (100%)	in vitro (CB, TK)	IMI: 38%; MER: 40%; DOR: 45%	-	-	-	-	-	FOS in association with a carbapenem was observed to reduce also biofilm formation.	[150]
	2013, Brazil	dos Santos	imipenem	4	-	4 (100%)	2 (50%)	in vitro (CB)	4 (100%)	0%	0%	0%	3 (75%)	1 (50%)	-	[48]
	2013, Austria	Lingscheid	doripenem	18	-	-	-	in vitro (CB, TK)	0%	0%	18 (100%)	0%	-	-	The authors reported FICI ranging from 0.5 to 4, without distinction between additive and indifferent effect, and considered the combination "indifferent" against all isolates.	[139]
	2012, Greece	Samonis	imipenem, meropenem, doripenem	15	-	1 (1%)	9 (60%)	in vitro (ET)	IMI: 7 (46%); MER: 8 (53%); DOR: 11 (73%)	IMI: 8 (53%); MER: 7 (46%); DOR: 4 (26%)	0%	0%	-	-	-	[86]
	2005, Thailand	Pruekprasert	imipenem	29	-	-	-	in vitro (CB)	11 (38%)	4 (14%)	12 (41%)	2 (7%)	-	-	-	[22]
	2002, Japan	Okazaki	imipenem, meropenem	30	-	15 (50%)	IMI: 29 (96%); MER: 27 (90%)	in vitro (efficacy time index)	IMI: 22 (73%); MER: 26 (86%)	IMI: 0%; MER: 2 (6%)	IMI: 8 (26%); MER: 2 (6%)	0%	IMI: 2 (13%); MER: 3 (20%)	IMI: 21 (72%); MER: 16 (59%)	-	[39]
	1999, Japan	Hayami	meropenem	26	-	NA (at least 13)	NA (at least 5)	in vitro (CB, TK)	3 (11%)	15 (57%)	8 (30%)	0%	-	-	-	[130]
	1997, France	Tessier	imipenem	40	-	20 (50%)	9 (22%)	in vitro (CB)	0%	15 (37%)	25 (62%)	0%	17 (85%)	8 (88%)	Although the combination had a synergistic effect on no tested strains, it is of clinical relevance as it restored FOS and IMI susceptibility in almost all R isolates.	[131]
*A. baumannii*	2019, USA	Flamm	meropenem	5 (*A. baumannii-calcoaceticus* species complex)	-	-	-	in vitro (CB, TK)	1 (20%)	3 (60%)	0%	0%	-	-	For 1 isolate the efficacy of FOS + MER remained indeterminate.	[38]
	2018, China	Zhu	imipenem	21	-	20 (95%)	21 (100%)	in vitro (CB)	12 (57%)	3 (14.3%)	6 (28%)	0%	-	-	-	[151]
	2018, Thailand	Singkham-In	imipenem, meropenem	23	OXA (100%)	23 (100%)	23 (100%)	in vitro (CB, TK)	IMI: 65%; MER: 0%	IMI: 30.4%; MER: 87%	IMI: 4%; MER: 13%	0%	-	-	-	[152]
	2016, Brazil	Leite	imipenem, meropenem	20	OXA (100%), IMP (15%)	20 (100%)	20 (100%)	in vitro (CB, TK)	IMI: 0%; MER: 0%	IMI: 4 (20%); MER: 0%	IMI: 16 (80%); MER: 100%	0%	-	-	-	[83]
	1996, Spain	Martinez-Martinez	imipenem	34	-	34 (100%)	NA (at least 7)	in vitro (CB)	1 (3%)	-	-	0%	-	-	The Authors reported only the number of isolates on which the combination had a synergistic or an antagonistic effect.	[132]
*S. aureus*	2019, Spain	Coronado-Álvarez	imipenem	4	MRSA (50%)	-	-	in vitro (TK)	4 (100%)	0%	0%	0%	-	-	-	[63]
	2015, Spain	del Río	imipenem	10 (in vitro); 2 (in vivo)	MRSA (100%)	1 (10%)	4 (40%)	in vitro (TB); in vivo (rabbit, endocarditis)	in vitro: 9 (90%); in vivo: 2 (100%)	in vitro: 1 (10%)	0%	0%	-	-	% of sterile vegetations: FOS alone 0%, IMI alone 7%, FOS + IMI 73%.	[28]
	2013, Austria	Lingscheid	doripenem	39	MRSA (100%)	0%	-	in vitro (CB, TK)	37 (94%)	-	-	0%	-	-	The authors reported FICI ranging from 0.5 to 4, without distinction between additive and indifferent effect.	[139]
	2012, Spain	Garrigós	imipenem	1	MRSA (100%)	0%	0%	in vitro (TK); in vivo (rat, foreign-body infection)	0%	in vitro: 1 (100%)	in vitro: 0%; in vivo: 1 (100%)	0%	-	-	-	[37]
	2011, Spain	Pachón-Ibáñez	imipenem	1	GISA (100%)	0%	100%	in vitro (TK); in vivo (mouse, peritonitis)	in vitro: 1 (100%); in vivo: 1 (100%)	0%	0%	0%	-	-	FOS + IMI reached statistical difference when compared to IMI as single therapy in the mouse model.	[36]
	2003, Japan	Nakazawa	imipenem, panipenem	32	MRSA (100%)	29 (91%)	28 (88%)	in vitro (efficacy time index)	IMI: 16 (50%); PAN: 21 (66%)	IMI: 3 (9%); PAN: 8 (25%)	IMI: 13 (41%); PAN: 3 (9%)	0%	-	-	-	[18]
	1987, France	Quentin	imipenem	5	-	1 (20%)	1 (20%)	in vitro (TK)	1 (20%)	0%	3 (60%)	1 (20%)	-	-	-	[35]
*S. aureus + S. epidermidis*	2001, Austria	Grif	meropenem	5 *S. aureus* + 2 *S. epidermidis*	MRSA (25%), GISA (25%)	-	-	in vitro (CB, TK)	*S. aureus*: 5 (100%)	0%	*S. epidermidis*: 2 (100%)	0%	-	-	-	[43]
	1992, Austria	Guggenbichler	imipenem	1 *S. aureus* + 2 *S. epidermidis*	-	-	-	in vitro (TK)	3 (100%)	0%	0%	0%	-	-	The study was conducted on catheters infected in laboratory. Bacterial regrowth was observed in catheters treated with FOS or IMI alone, but did not occurred when the drugs were tested in combination.	[153]
*Staphylococcus* spp. + *Enterococcus* spp.	1986, Italy	Debbia	imipenem	76	-	-	-	in vitro (CB, TK)	54 (71%)	0%	22 (29%)	0%	-	-	% reported are those obtained with CB. Results of TK showed higher rates of synergism, but in the present Table are considered the results of CB as not all isolates were tested with TK.	[154]
*E. faecalis*	2011, Italy	Farina	imipenem	27	-	2 (7%)	0%	in vitro (ET)	0%	0%	10 (37%)	17 (62%)	-	-	The Authors did not distinguish between additive and indifferent effect, and defined the effect of FOS + IMI indifferent.	[128]
*S. pneumoniae*	1994, France	Doit	imipenem	26	-	0%	0%	in vitro (TK)	0%	26 (100%)	0%	0%	-	-	-	[134]
*N. gonorrhoeae*	2015, The Netherlands	Wind	ertapenem	4	-	-	-	in vitro (ET)	0%	3 (75%)	1 (25%)	0%	-	-	-	[54]

**Table 4:** Studies on combination between fosfomycin and aztreonam. CB: checkerboard assay; TK: time–kill assay; ET: E-test.

Strain	Year and Country	Author	Number of Isolates	Known Resistance Mechanisms or Determinants (%)	FOS-Resistant (%)	Aztreonam-Resistant (%)	In Vitro (Methods)/In Vivo (Animal and Site of Infection)	Synergistic Effect (%)	Additive Effect (%)	Indifferent Effect (%)	Antagonistic Effect (%)	FOS Susceptibility Restoration (%)	Aztreonam Susceptibility Restoration (%)	Comments	Reference
Enterobacterales	2019, USA	Avery	48	48 not specified between: 8 *E. coli*: KPC (25%), NDM (75%), ESBL (62%); 35 *Klebsiella* spp: KPC (45%), NDM (40%); OXA (14%), VIM (8.%), ESBL (88%), fosA (44%); 2 *Citrobacter* spp: KPC (50%), NDM (50%), ESBL (50%), 4 *E. cloacae*: KPC (75%), NDM (25%), ESBL (75%)	20 (40%)	48 (100%)	in vitro (ET)	4 (8%)	13 (27%)	31 (64%)	0%	0%	0%	Data on synergism reported without distinction for bacterial strains. % of FOS-R isolates estimated on the basis of the reported MIC50.	[11]
	2019, USA	Flamm	20	-	-	-	in vitro (CB, TK)	5 (25%)	5 (25%)	1 (5%)	0%	-	-	For 9 isolates the efficacy of FOS + ATM remained indeterminate.	[38]
*E. coli*	2014, Sweden	Hickam	2	ESBL, OXA (50%)	0%	1 (50%)	in vitro (CB, TK)	2 (100%)	0%	0%	0%	-	-	-	[120]
*K. pneumoniae*	2014, Sweden	Hickam	1	ESBL, OXA (100%)	0%	1 (100%)	in vitro (CB, TK)	0%	1 (100%)	0%	0%	-	-	-	[120]
*P. aeruginosa*	2019, USA	Avery	103	-	NA (at least 71)	103 (100%)	in vitro (ET)	16 (15.5%)	68 (66%)	19 (18%)	0%	-	21 (13%)	-	[33]
	2019, USA	Flamm	5	-	-	-	in vitro (ET)	1 (20%)	3 (60%)	0%	0%	-	-	For 1 isolate the efficacy of FOS + ATM remained indeterminate.	[38]
	2002, Japan	Okazaki	30	-	15 (50%)	29 (96%)	in vitro (efficacy time index)	23 (76.%)	3 (10%)	4 (13%)	0%	4 (26%)	6 (20%)	-	[39]

**Table 5:** Studies on combination between fosfomycin and quinolones. CB: checkerboard assay; TK: time–kill assay; ET: E-test.

Strain	Year and Country	Author	Quinolone	Number of Isolates	Known Resistance Mechanisms or Determinants (%)	FOS-Resistant (%)	Quinolone-Resistant (%)	In Vitro (Methods)/In Vivo (Animal and Site of Infection)	Synergistic Effect (%)	Additive Effect (%)	Indifferent Effect (%)	Antagonistic Effect (%)	FOS Susceptibility Restoration (%)	Quinolone Susceptibility Restoration (%)	Comments	Reference
Enterobacterales	2019, USA	Flamm	Levofloxacin	20	7 MDR (of which 29% ESBL and 29% KPC-producer)	-	-	in vitro (CB)	30%	60%	10%	0%	-	-	-	[38]
*E. coli*	2020, Egypt	El-Wafa	Ciprofloxacin	8	-	100%	100%	in vitro (CB, TK)	3 (37%)	-	-	-	3 (100%)	3 (100%)	Triple combination (FOS/IMP/CIP o FOS/CIP/TOB) increased synergism against all isolates.	[42]
	2019, USA	Wang	Ciprofloxacin	8	-	25%	25%	in vitro (ET, biofilm)	2 (25%)	-	6 (75%)	-	0%	0%	-	[155]
	2019, India	Sugathan	Ciprofloxacin	50	biofilm producers (100%)	0%	98%	in vitro (CB, TK)	3 (6%)	20 (40%)	27 (54%)	0%	-	0%	The optimal combination of fosfomycin with N-acetylcystein produces the reduction of *E. coli* sessile cell viability and biofilm formation up to 60–73%.	[137]
*S. flexneri*	2019, China	Liu	Ciprofloxacin	80	-	43 (54%)	100%	in vitro (CB, TK); in vivo (*Galleria mellonella*)	31 (38%)	0%	49 (61)%	0%	65 (81%)	3 (4%)	-	[156]
*P. aeruginosa*	2019, USA	Wang	Ciprofloxacin	7	-	0%	14%	in vitro (ET, biofilm)	4 (57%)	-	3 (42%)	-	-	0%	-	[155]
	2019, USA	Flamm	Levofloxacin	5	7 MDR (of which 29% ESBL and 29% KPC-producer)	-	-	in vitro (CB)	1 (20%)	4 (80%)		0%	-	-	-	[38]
	2016, Australia	Walsh	Ciprofloxacin	4	-	75%	50%	in vitro (TK)	21% (23/108)	15% (16/108)	38% (41/108)	-	-	-	The total number of experiments was 108 (9 combinations of FOS + CIP at different concentrations, in 3 different times).	[76]
	2013, Brazil	Dos Santos	Ciprofloxacin	2	MDR (50%)	100%	50%	in vitro (CB, TK)	2 (100%)	-	-	-	2 (100%)	0%	-	[48]
	2007, Japan	Mikuniya	Prulifloxacin, ciprofloxacin, levofloxacin	1	biofilm forming (100%)	-	-	in vivo (rat, UTI)	1 (100%)	-	-	-	-	-	*After 3 consecutive days’ co-administration.	[40]
	2007, Japan	Yamada	Ciprofloxacin	74	-	-	-	in vitro (CB)	20 (27%)	-	54 (73%)	0%	-	-	-	[157]
	2005, Japan	Micuniya	Ciprofloxacin, Ulifloxacin, Levofloxacin	1	-	100%	100%	in vitro (ATP bioluminescence assay)	-	100%	-	-	0%	0%	-	[46]
	2002, Japan	Monden	Ofloxacin	4	-	3 (75%)	1 (25%)	in vitro (biofilm)	3 (75%)	-	-	-	-	-	-	[158]
	2001, Japan	Okazaki	Levofloxacin	30	MDR (50%)	13/30 (43%)	21/30 (70%)	in vitro (Efficacy time index)	3/30 (1%)	17/30 (56%)	-	10/30 (33)%	-	-	ETI < 0.5 antagonism; 0.5 ≤ ETI <1 indifferent; 1 ≤ ETI < 8 additive; ETI ≥ 8 synergistic	[39]
	1999, Japan	Hayami	Ciprofloxacin	26	-	-	-	in vitro (CB, TK)	10(38%)	15 (57%)	1 (3%)	0%	-	-	-	[130]
	1997, France	Bugnon	Pefloxacin	2	-	-	-	in vivo (rabbit, endocarditis)	-	-	-	100%	-	-	-	[41]
	1997, France	Tessier	Ciprofloxacin	40	MDR (100%)	23 (57%)	19 (47%)	in vitro (CB)	6 (15%)	32 (80%)	2 (5%)	-	16 (70%)	12 (63%)	-	[131]
	1995, Japan	Kumon	Ofloxacin	1	-	-	-	in vitro (TK)	1 (100%)	-	-	-	-	-	-	[159]
	1994, France	Xiong	Ciprofloxacin	2	MDR (50%)	0%	50%	in vitro (CB); in vivo (rabbit, endocarditis)	2 (100%) early thp; 1 (50%) Late thp	0% early thp; 1 (50%) Late thp	-	-	-	-	in vivo results.	[160]
	1994, France	Xiong	Pefloxacin	2	MDR (50%)	0%	50%	in vitro (CB); in vivo (rabbit, endocarditis)	1 (50%) early thp; 1 (50%) late thp	1 (50%) early thp	1 (50%) late thp	-	-	-	in vivo results.	[160]
	1989, Germany	Vogt	Ciprofloxacin	25	-	1 (4%)	2 (8%)	in vitro (TK)	20%	-	-	-	-	-	-	[161]
	1988, USA	Figueredo	Ciprofloxacin	-	-	-	-	in vitro (CB)	60% (EV) 17% (OS)	-	-	0%	-	-	-	[162]
	1987, Germany	Ullmann	Ciprofloxacin	37	-	-	-	in vitro (CB)	29 (78%)	8 (22%)	0%	0%	100%	-	-	[45]
*A. baumannii*	1996, Spain	Martinez-Martinez	Ciprofloxacin	34	-	100%	100%	in vitro (CB)	1 (3%)	-	-	0%	-	-	-	[132]
*A. baumannii-A. calcoaceticus* spp. complex	2019, USA	Flamm	Levofloxacin	5	7 MDR (29% ESBL and 29% KPC-producer)	-	-	in vitro (CB)	0%	4 (80%)	1 (20%)	0%	-	-	-	[38]
Gram negative	1977, Spain	Daza	Nalidixic acid	100	-	100%	-	in vitro (CB)	0%	-	100%	0%	-	-	*Klebsiella* spp., *Pseudomonas* spp., *E. coli*, *Serratia* spp., *Proteus* spp., *Enterobacter* spp., *Acinetobacter* spp., *Levinea* spp.	[66]
*Staphylococcus* spp.	2003, Japan	Nakazawa	Ofloxacin	32	MRSA (100%)	-	-	in vitro (efficacy time index)	3 (9%)	2 (6%)	27 (84%)	-	-	-	synergism = high efficacy; additive = efficacy; indifferent = invalid	[18]
	2001, Austria	Grif	Moxifloxacin	7	MRSA (100%)	-	-	in vitro (CB)	100%	-	-	-	-	-	-	[43]
	1997, Italy	Ferrara	Sparfloxacin	16	MRSA (100%)	>50%	∼100%	in vitro (TK)	0%	-	-	-	-	-	-	[123]
	1988, France	Thauvin	Pefloxacin	1	MRSA (100%)			in vivo (rat, endocarditis)	100%	-	-	-	-	-	-	[44]
	1987, France	Weber	Ofloxacin	8	MRSA (37%)	-	-	in vitro (TK)	2 (25%)	6 (75%)	-	-	-	-	-	[163]
	1987, Germany	Ullmann	Ciprofloxacin	20	-	-	-	in vitro (CB)	19 (95%)	1 (5%)	-	-	-	-	*S. aureus*.	[45]
	1987, France	Quentin	Pefloxacin	6	-	16%	0%	in vitro (TK)	0%	0%	100%	0%	-	-	*S. aureus*. Indifferent effect.	[35]
*S.epidermidis*	1997, Italy	Ferrara	Sparfloxacin	12	MRSE (100%)	>50%	∼100%	in vitro (TK)	6/12 (50%)	-	-	-	-	-	-	[123]
	1987, France	Quentin	Pefloxacin	2	-	50%	-	in vitro (TK)	0%	0%	100%	0%	-	-	Indifferent effect.	[35]
*N. gonorrhoeae*	2014, Netherlands	Wind	Moxifloxacin	4	-	-	-	in vitro (ET)	0%	-	-	-	-	-	-	[54]

**Table 6:** Studies on combination between fosfomycin and aminoglycosides. CB: checkerboard assay; TK: time–kill assay; ET: E-test.

Strain	Year and Country	Author	Aminoglycoside	Number of Isolates	Known Resistance Mechanisms or Determinants (%)	FOS-Resistant (%)	Aminoglycoside-Resistant (%)	In Vitro (Methods)/In Vivo (Animal and Site of Infection)	Synergistic Effect (%)	Additive Effect (%)	Indifferent Effect (%)	Antagonistic Effect (%)	FOS Susceptibility Restoration (%)	Aminoglycoside Susceptibility Restoration (%)	Comments	Reference
Enterobacterales	2019, USA	Avery	Tobramycin	45	45 not specified between: 8 *E. coli*: KPC (25%), NDM (75%), ESBL (62%); 35 *Klebsiella* spp: KPC (45%), NDM (40%); OXA (14%), VIM (8%), ESBL (88%), fosA (44%); 2 *Citrobacter* spp: KPC (50%), NDM (50%), ESBL (50%), 4 *E. cloacae*: KPC (75%), NDM (25%), ESBL (75%)	20/49 (40%)	45 (100%)	in vitro (ET)	2 (4%)	7 (15%)	36 (80%)	0%	-	-	Data on synergism reported without distinction for bacterial strains. Percentages of FOS-R isolates estimated on the basis of the reported MIC50.	[11]
	2019, USA	Flamm	Gentamicin	20	-	-	-	in vitro (CB, TK)	6 (30%)	13 (65%)	1 (5%)	0%	-	-	-	[38]
	1978, Spain	Olay	Streptomycin, gentamicin, kanamycin	Streptomycin: 18 *E. coli*. Gentamicin: 30 *E. coli*, 24 *Klebsiella* spp., 39 *S. marcescens*, 33 *Proteus* spp. Kanamycin: 21 *E. coli*, 12 *Klebsiella* spp., 16 *Proteus* spp., 5 *E. cloacae*, 22 *S. marcescens*	-	-	-	in vitro (CB)	streptomycin: 0%; gentamicin: 16 (12%); kanamycin: 21 (27%)	streptomycin: 9 (50%); gentamicin: 52 (41%); kanamycin: 37 (48%)	streptomycin: 9 (50%); gentamicin: 58 (46%); kanamycin: 18 (23%)	0%	-	-	-	[14]
*E. coli*	2020, Egypt	El-Wafa	tobramycin	8	-	3 (37.5%)	8 (100%)	in vitro (CB, TK)	2 (25%)	0%	0%	0%	2 (66%)	2 (25%)	For 6 isolates the efficacy of FOS + TOB remained indeterminate.	[42]
	2019, USA	Wang	Gentamicin	8	-	0%	2/8 (25%)	in vitro (ET, biofilm)	75% (6/8)	0%	(2/8) 25%	0%	-	1/2 50%	-	[155]
	2019, India	Sugathan	Amikacin	50	-	0%	26 (52%)	in vitro (TK)	29 (58%)	21 (42%)	0%	0%	0% (all S)	22 (84%)	The Authors also studied the efficacy of combination of FOS + AMK and found it reduced significantly biofilm formation.	[137]
	2013, Switzerland	Corvec	Gentamicin	1	CTX-M15, ESBL	0%	0%	in vitro (TK); in vivo (foreign-body infection model)	0%	100%	0%	0%	-	-	Cure rate of FOS plus gentamicin 42%.	[73]
	2011, Greece	Samonis	Netilmicin	20	ESBL	0%	35%	in vitro (ET)	25% (5/20)	-	-	-	-	-	-	[86]
	1977, Poland	Borowski	Streptomycin	10	-	-	-	in vitro (CB)	7 (70%)	3 (30%)	0%	0%	-	-	-	[121]
*K. pneumoniae*	2020, Turkey	Erturk Sengel	Amikacin	17	OXA-48, NDM	41%	76%	in vitro (CB)	29%	29%	24%	0%	-	-	Combination of FOS plus amikacin seems not a good choice for NDM producing strains.	[142]
	2018, China	Yu	Amikacin	3	-	0%	-	in vitro (TK)	100% (3/3)	0%	0%	0%	-	-	-	[164]
	2017, China	Yu	Amikacin	3	KPC-2	0%	33%	in vitro (TK)	66%	0%	33%	0%	-	-	FOS (8 g q8h)/AMK (15 mg/kg qd) most bactericidal activity, but resistance occurred.	[50]
	2017, China	Yu	Amikacin	136	KPC (100%)	78 (57%)	80 (58%)	in vitro (CB, TK)	7 (5%)	109 (80%)	20 (14%)	0%	-	-	-	[89]
	2015, Spain	Rodriguez-Avial et al.	Plazomicin	4 (CB); 2 (TK)	Carbapenemase-producing strains (KPC, VIM)	100%	NA	in vitro (CB, TK)	25–100%	50–0%	25–0%	0%	-	-	-	[51]
	2014, USA	Montgomery	Amikacyn	20	KPC-2 (20%), KPC-3 (15%)	-	100%	in vitro (agar diluition, antibiotic potentation study in A:F 5:2 ratio)	100%	-	-	0%	-	-	Synergy defined: reduction of FOS and AMK MIC when used in combination.	[52]
	2011, Greece	Samonis	Netilmicin	65	serine carbapenem-producing (50/65); ESBL (14/65); MBL (1/65)	98%	87%	in vitro (ET)	41% (27/65) overall. In ESBL 42% (6/14). In serine enzymes 42% (21/50)	-	-	-	-	54% (25/46)	-	[86]
	2011, Greece	Souli	gentamicin	17	KPC (100%)	4 (23%)	7 (41%)	in vitro (TK)	0%	0%	15/15 (100%)	-	-	-	Efficacy of FOS + GEN was not evaluated in 2 isolates.	[53]
	1977, Spain	Daza	Tobramicin	23	-	-	-	in vitro (CB)	2/23 (8%)	-	-	0%	-	-	-	[66]
*M. morganii*	1977, Spain	Daza	Gentamicin	2	-	-	-	in vitro (CB)	50% (1/2)	-	-	0%	-	-	-	[66]
*P. aeruginosa*	2019, USA	Wang	Gentamicin	7	-	25%	1/7 (14%)	in vitro (ET, biofilm)	4 (57%)	0%	3 (42%)	0%	-	0%	-	[155]
	2019, USA	Avery	tobramycin	42	-	NA (at least 71)	42 (27%)	in vitro (ET)	8 (19%)	13 (31%)	21 (50%)	0%	-	8 (19%)	-	[33]
	2019, New Zealand	Li Bassi	Amikacin	15	Strains resistant to nebulized fosfomycin and amikacin (100%)	-	-	in vivo (pigs, pneumonia)	0%	0%	100%	0%	-	-	No difference in *P. aeruginosa* lung tissue concentration, bronchoalveolar lavage concentration and lung hystopathology score when amikacin and FOS were administered by aerosol alone or in combination therapy.	[165]
	2019, USA	Flamm	gentamicin, amikacin	5	-	-	-	in vitro (CB, TK)	0%	genta: 4 (80%); amika: 4 (80%)	genta: 1 (20%); amika: 1 (20%)	0%	-	-	-	[38]
	2018, Spain	Diez-Aguilar	Tobramycin	6	-	100%	67%	in vitro (CB)	83%	17%	0%	0%	-	-	Synergy tested in biofilm.	[166]
	2015, Australia	Walsh	Tobramycin	3	-		1/4 (25%)	in vitro (TK)	18% (15/81)	25% (20/81)	-	-	-	-	-	[76]
	2015, Spain	Diez-Aguilar	Tobramycin	8	mexZ mutation (25%), ANT(2’)-I enzyme (37.5%),	100%	37%	in vitro (TK)	25%	0%	75%	0%	-	-	-	[166]
	2014, USA	Montgomery	Amikacin	21	GES-1, OXA-2 plus OXA-10 plus VIM-2, OXA 14, VIM-4 (each, 4.8%), VIM-2 (19%)	-	100%	in vitro (agar dilution, antibiotic potentation study in A:F 5:2 ratio)	100%	-	-	-	-	-	Synergy defined: reduction of FOS and AMK MIC when used in combination.	[52]
	2013, Brazil	Ferrari dos Santos Lima	Tobramycin	2	IMP-R (100%)	100%	100%	in vitro (broth microdilution, CB)	100%	0%	0%	0%	100%	0%	Authors do NOT report FOS and AMG MIC (they referred to CLSI criteria except for FOS-Eucast S ≤ 32 µg/mL); FOS MIC restoration 32.	[48]
	2013, USA	Anderson	Tobramycin	1	-	-	-	in vitro (effects on biofilms on CF airway epithelial cells)	-	100%	0%	0%	-	-	FOS:TOBRA (4:1) formulas for inhalation treatment; results suggest that fosfomicon enhanced the activity of tobramycin (much less level of tobramycin needed). FOS alone does NOT result in biofilm inhibition, TOBRA alone require HIGHER doses for biofilm inhibition.	[49]
	2012, UK/USA	McCaughey	Tobramycin	15	-	-	-	in vitro (agar dilution, TK)	100%	-	-	0%	-	-	Synergism defined as FOS:TOBRA bactericidal activity; Time kill studies in a subset of isolates; biofilm studies were also performed.	[167]
	2011, Greece	Samonis	Netilmicin	15	MDR	93%	13%	in vitro (ET)	13% (2/15)	-	-	-	-	-	-	[86]
	2009, China	Cai	Amikacin	20	-	-	NA (MIC90 32)	in vitro (CB); in vivo (rat, biofilm-infected model)	80%	15%	-	0%	-	MIC90 decrease of 64-fold	F + T (lowest FICI amikacina and isepamicina) had synergistic effect on planctonic *P. aeruginosa*.	[168]
	2009, China	Cai	Gentamicin	20	-	-	NA (MIC90 16)	in vitro (CB); in vivo (rat, biofilm-infected model)	70%	15%	-	0%	-	MIC90 decrease of 8-fold	F + T (lowest FICI amikacina and isepamicina) had synergistic effect on planctonic *P. aeruginosa*.	[168]
	2009, China	Cai	Netilmicin	20	-	-	NA (MIC90 16)	in vitro (CB); in vivo (rat, biofilm-infected model)	65%	20%	-	0%	-	MIC90 decrease of 8-fold	F + T (lowest FICI amikacina and isepamicina) had synergistic effect on planctonic *P. aeruginosa*.	[168]
	2009, China	Cai	Tobramycin	20	-	-	NA (MIC90 8)	in vitro (CB); in vivo (rat, biofilm-infected model)	60%	20%	-	0%	-	MIC90 decrease of 2-fold	F + T (lowest FICI amikacina and isepamicina) had synergistic effect on planctonic *P. aeruginosa*.	[168]
	2005, Thailand	Pruekprasert	gentamicin	22	-	-	-	in vitro (CB)	1 (4%)	9 (42%)	6 (27%)	6 (27%)	-	-	-	[22]
	2002, Japan	Okazaki	gentamicin	30	-	15 (50%)	19 (63%)	in vitro (efficacy time index)	0%	9 (30%)	21 (70%)	0%	0%	15 (50%)	-	[39]
	1999, Japan	Hayami	amikacin	26	-	NA (at least 13)	NA (< 5)	in vitro (CB, TK)	0%	10 (38%)	16 (61%)	0%	-	-	-	[130]
	1991, Nigeria	Chinwuba	Gentamicin	8	-	-	0%	in vitro (CB, TK)	0%	0%	100%	0%	-	-	-	[169]
	1997, France	Tessier	amikacin	40	-	23 (57%)	13 (32%)	in vitro (CB)	3 (7%)	21 (52%)	16 (40%)	0%	18 (78%)	11 (84%)	Although the combination had a synergistic effect on no tested strains, it is of clinical relevance as it restored FOS and AMK susceptibility in many resistant strains.	[131]
	1978, Spain	Olay	gentamicin, kanamycin	77 gentamicin, 15 kanamycin	-	-	-	in vitro (CB)	gentamicin: 55 (71%); kanamycin: 4 (26%)	gentamicin: 17 (22%); kanamycin: 8 (53%)	gentamicin: 5 (6%); kanamycin: 3 (20%)	0%	-	-	-	[14]
*A. baumannii*	2019, USA	Flamm	gentamicin, amikacin	5 (*A. baumannii*-*calcoaceticus* species complex)	-	-	-	in vitro (CB, TK)	genta: 2 (40%); amika: 2 (40%)	genta: 3 (60%); amika: 3 (60%)	0%	0%	-	-	-	[38]
	2016, Brazil	Leite	gentamicin, amikacin	20	OXA (100%), IMP (15%)	20 (100%)	genta: 11 (55%); amika: 19 (95%)	in vitro (CB, TK)	0%	genta: 2 (10%); amika: 0%	genta: 18 (90%); amika: 20 (100%)	0%	-	-	"2-well" method showed synergistic activity in about 20% of tested strain, but the Authors considered it not fully reliable and concluded the association had an indifferent effect.	[83]
	2014, USA	Montgomery	Amikacyn	21	OXA-23 plus OXA-51 (23.8%); OXA-24 plus OXA-51 (9.5%), OXA-51, OXA-51 plus OXA-58 (each, 4.8%)	-	100%	in vitro (agar dilution, antibiotic potentation study in A:F 5:2 ratio)	100%	-	-	0%	-	-	Synergism defined as reduction of FOS and AMK MIC when used in combination.	[52]
	1996, Spain	Martinez-Martinez	amikacin, tobramycin	34	-	34 (100%)	amika: 31 (91%); tobra: 33 (97.%)	in vitro (CB)	amika: 15 (44%); tobra: 11 (32%)	-	-	0%	-	-	The authors reported only synergistic and antagonistic effect rates.	[132]
Gram-negative	1977, Spain	Daza	Tobramycin	75	-	-	-	in vitro (CB)	0%	0%	100%	0%	-	-	33 *Klebsiella* spp., 21 *P. aeruginosa*, 3 *P. cepacia*, 12 *E.coli*,11 *S. marcescens*, 9 *Enterobacter* spp., 8 *Proteus* spp., 2 *A. calcoaceticus*, 1 *L. malonatica*, 5 *K. pneumoniae oxytoca*, 5 *K. Ozenae*, 5 *E. aerogenes*, 2 *E. hafniae*, 1 *E. cloacae*, 1 *E. liquefaciens*, 4 *P. mirabilis*, 2 *P. rettgeri*	[66]
	1977, Spain	Daza	Gentamicin	75	-	-	-	in vitro (CB)	0%	0%	100%	0%	-	-		[66]
*S. aureus*	2017, Spain	Lopez Diaz	Plazomicin	12 (BC); 5 (TK)	MRSA Strains carrying aminoglycosides-modifying enzymes (100%)	56%	-	in vitro (CB, TK)	33.3–0%	66–100%	0%	0%	-	-	-	[170]
	2012, UK/USA	McCaughey	Tobramycin	5	MRSA	100%	-	in vitro (agar dilution, TK)	60%	-	-	0%	-	-	Synergism defined as F:T bactericidal activity; Time kill studies in a subset of isolates; biofilm studies were also performed	[167]
	2005, Japan	Morikawa	Arbekacin	1	MRSA	100%	100% MIC 0.5 (no available breakpoint)	in vivo (rat, carboxymethyl cellulose pouch infection model)	100%	-	-	-	-	-	NOT available arbikacin EUCAST breakpoints; Synergistic effect was evaluated by i) morphological and histological studies showing dramatic change in biofilm and inflammatory response and by ii) decrease in the number of viable bacteria in vivo.	[171]
	1994, Japan	Kono	Arbekacin	96	MRSA	38%	-	in vitro	66% (60/90)	-	-	0%	-	-	Better results of FOS-arbekacin combination in FOS susceptible strains.	[172]
	1987, Spain	Rodriguez	Gentamicin	1	MRSA	0%	0%	in vivo (endocarditis in 10 rabbits)	100% (1/1) 0% n. of rabbits’ death (0/10)	0%	0%	0%	-	-	-	[61]
	1985, USA	Alvarez	Gentamicin	148	MRSA	-	-	in vitro (microtiter technique in a 1:1 ratio)	(10/148) 7%	0%	90% (134/148)	(4/148) 3%	-	-	Synergy was indicated if the MICs of both drugs decreased by at least one-fourth. If the MIC of one drug owed a fourfold or greater increase, it was assumed to be an indication of antagonism.	[12]
	1978, Spain	Olay	streptomycin, gentamicin, kanamycin	18 streptomycin, 29 gentamicin, 21 kanamycin	-	-	-	in vitro (CB)	streptomycin: 1 (5%); gentamicin: 0%; kanamycin: 9 (43%)	streptomycin: 10 (55%); gentamicin: 3 (10%); kanamycin: 7 (33%)	streptomycin: 7 (38%); gentamicin: 26 (89%); kanamycin: 5 (23%)	0%	-	-	-	[14]
*Streptococcus* spp.	1978, Spain	Olay	streptomycin	16	-	-	-	in vitro (CB)	0%	9 (56%)	7 (43%)	0%	-	-	-	[14]
*E. faecium*	2019, Thailand	Hemapanpairoa	Gentamicin	8	VRE (100%)	100%	13%	in vitro (ET for FOS, broth microdilution for gentamicin)	63%	13%	25%	0%	63%	-	Synergistic activity assessed as a fourfold reduction of MIC when FOS combined with gentamicin 1 mcg/mL.	[55]
*N. gonorrhoeae*	2015, The Netherlands	Wind	gentamicin	4	-	-	-	in vitro (ET)	0%	1 (25%)	3 (75%)	0%	-	-	-	[54]
Miscellaneous	2009, USA	MacLeod	Tobramycin	27 (4 *S. aureus*, 17 *P. aeruginosa*, 5 *E.coli*, 1 *H. influenzae*)	-	-	-	in vitro (CB, TK); in vivo (rat, pneumonia)	7% (1 *P. aeruginosa*, 1 *E.coli*)	-	93%	0%	-	-	In vitro (agar plate dilution, broth microdilution, CB ON a SUBSET of ISOLATES, TK) and in vivo (rat bacterial pneumonia). NB: CB for 27 total strains: 4 *S. aureus*, 17 *P. aeruginosa*, 5 *E. coli*, 1 *H. influenzae*. FOS:TOBRA 4:1 was rapidly bactericidal and exhibited concentration -bactericidal killing in TK, with excellent activity against *S. aureus* and *H. influenzae*, but poor activity against *S. maltophilia*, *B. cepacia*; it was active against *M. catarrhalis*, *E. coli*, *Klebsiella* and *S. pneumoniae*.	[173]

**Table 7:** Studies on combination between fosfomycin and macrolides. CB: checkerboard assay; TK: time–kill assay; ET: E-test.

Strain	Year and Country	Author	Macrolide	Number of Isolates	Known Resistance Mechanisms or Determinants (%)	FOS-Resistant (%)	Macrolide-Resistant (%)	In Vitro (Methods)/In Vivo (Animal and Site of Infection)	Synergistic Effect (%)	Additive Effect (%)	Indifferent Effect (%)	Antagonistic Effect (%)	FOS Susceptibility Restoration (%)	Macrolide Susceptibility Restoration (%)	Comments	Reference
*E. coli*	1978, Spain	Olay	Erythromycin	14	-	-	-	in vitro (CB)	42%	29%	28%	0%	-	-	Authors considered Synergistic effect when MIC of both antimicrobials was at least fourfold lower over initial MIC; partial synergy when MIC of one antimicrobials was at least fourfold lower and MIC of the other one 2 times lower over initial MIC; Indifferent effect when MIC of both antimicrobials was 2 times lower; antagonism when MIC of both increased 4 times over initial MIC.	[14]
*Klebsiella spp*.	1978, Spain	Olay	Erythromycin	44	-	-	-	in vitro (CB)	50%	23%	27%	0%	-	-	Authors considered Synergistic effect when MIC of both antimicrobials was at least fourfold lower over initial MIC; partial synergy when MIC of one antimicrobials was at least fourfold lower and MIC of the other one 2 times lower over initial MIC; Indifferent effect when MIC of both antimicrobials was 2 times lower; antagonism when MIC of both increased 4 times over initial MIC.	[14]
*E. cloacae*	1978, Spain	Olay	Erythromycin	16	-	-	-	in vitro (CB)	62%	38%	0%	0%	-	-	Authors considered Synergistic effect when MIC of both antimicrobials was at least fourfold lower over initial MIC; partial synergy when MIC of one antimicrobials was at least fourfold lower and MIC of the other one 2 times lower over initial MIC; Indifferent effect when MIC of both antimicrobials was 2 times lower; antagonism when MIC of both increased 4 times over initial MIC.	[14]
*Proteus spp. (Indole +)*	1978, Spain	Olay	Erythromycin	13	-	-	-	in vitro (CB)	53%	46%	0%	0%	-	-	Authors considered Synergistic effect when MIC of both antimicrobials was at least fourfold lower over initial MIC; partial synergy when MIC of one antimicrobials was at least fourfold lower and MIC of the other one 2 times lower over initial MIC; Indifferent effect when MIC of both antimicrobials was 2 times lower; antagonism when MIC of both increased 4 times over initial MIC.	[14]
*P. aeruginosa*	1982, Japan	Kasai	Midecamycin	2	-	0%	2 (100%)	in vitro (TK)/in vivo (Mice, peritonitis or subcutaneous infection)	0%	2 (100%)	0%	0%	-	-	In all in vivo experiment survival rates of mice that received MDM + FOS was statistically significant higher then when FOS or MDM were administrated alone, proving synergistic effect.	[59]
	1978, Spain	Olay	Erythromycin	29	-	-	-	in vitro (CB)	38%	59%	3%	0%	-	-	Authors considered Synergistic effect when MIC of both antimicrobials was at least fourfold lower over initial MIC; partial synergy when MIC of one antimicrobials was at least fourfold lower and MIC of the other one 2 times lower over initial MIC; Indifferent effect when MIC of both antimicrobials was 2 times lower; antagonism when MIC of both increased 4 times over initial MIC.	[14]
*S. aureus*	1978, Spain	Olay	Erythromycin	34	-	-	-	in vitro (CB)	26%	68%	6%	0%	-	-	Authors considered Synergistic effect when MIC of both antimicrobials was at least fourfold lower over initial MIC; partial synergy when MIC of one antimicrobials was at least fourfold lower and MIC of the other one 2 times lower over initial MIC; Indifferent effect when MIC of both antimicrobials was 2 times lower; antagonism when MIC of both increased 4 times over initial MIC.	[14]
*S. epidermidis*	2009, Austria	Presterl	Azithromycin	11	-	2 (18%)	5 (45%)	in vitro (Microtitre plate assay on Biofilm culture)	-	-	-	-	-	-	Combination of azithromycin with any of the tested antimicrobial agents did not reduce the biofilm ODr compared to the ODr of biofilms treated with single agents	[58]
*S. pseudointermedius*	2014, Canada	DiCicco	Clarithromycin	8	MRSP (100%)	5 (62%)	8 (100%)	in vitro (Microtitre plate assay)	5 (62%)	2 (25%)	0%	0%	-	-	FICI for 1 strains was reported as "Not available".	[60]
*Streptococcus spp*.	1978, Spain	Olay	Erythromycin	26	-	-	-	in vitro (CB)	15%	27%	57%	0%	-	-	Authors considered Synergistic effect when MIC of both antimicrobials was at least fourfold lower over initial MIC; partial synergy when MIC of one antimicrobials was at least fourfold lower and MIC of the other one 2 times lower over initial MIC; Indifferent effect when MIC of both antimicrobials was 2 times lower; antagonism when MIC of both increased 4 times over initial MIC.	[14]
*N. gonorrhoeae*	2015, Switzerland	Hauser	Azithromycin	8 (4 TK)	AZT-HLR (12,%)	0%	1 (12%)	in vitro (CB, TK)	CK: 0%; TK: 0%	CK: 0%; TK: 0%	CK: 8 (100%); TK: 4 (100%)	CK: 0%; TK: 0%	-	-	Only 4 strains were tested with TKA. Authors used *Enterobacterales* FOS breakpoint as presumptive breakpoint for *N. gonorrhoeae* (EUCAST: S ≤ 32 mg/L; CLSI: S ≤ 64 mg/L).	[57]
	2015, Netherlands	Wind	Azithromycin	4	Azithromycin and Ceftriaxone Resistant (100%)	-	-	in vitro (ET)	0%	0%	4 (100%)	-	-	-	-	[54]

**Table 8:** Studies on combination between fosfomycin and glycopeptides. CB: checkerboard assay; TK: time–kill assay; ET: E-test.

Strain	Year and Country	Author	Glycopeptide	Number of Isolates	Known Resistance Mechanisms or Determinants (%)	FOS-Resistant (%)	Glycpeptide-Resistant (%)	In Vitro (Methods)/In Vivo (Animal and Site of Infection)	Synergistic Effect (%)	Additive Effect (%)	Indifferent Effect (%)	Antagonistic Effect (%)	FOS Susceptibility Restoration (%)	Glycopeptide Susceptibility Restoration (%)	Comments	Reference
*A baumannii*	2016, Brazil	Leite	Vancomycin	20	OXA-23 (50%), OXA-143 (35%), IMP-type (15%), depletion of OMP 43 kDa (20%)	19 (95%)	Natural resistance	in vitro (CB, TK)	0%	0%	CB: 20 (100%)	0%	0%	Breakpoints not available	TK showed indifference in all strains.	[83]
*S. aureus*	2018, China	Xu	Vancomycin	3	-	1 (33%)	0%	in vitro (CB)	0%	2 (66%)	1 (33%)	0%	1 (100%)	No resistant isolates	In vitro concentrations - VAN (0.5, 1, 2 mg/L); FOS (32, 64 mg/L).	[174]
	2017, Spain	Coronado-Alvarez	Vancomycin	4	Methicillin resistance (50%)	-	-	in vitro (TK)	0%	4 (100%)	0%	0%	-	-	The study also evaluated 15 patients with bacteremia caused by MRSA were treated with FOS in combination with VAN. Of these, 7 patients (46.7%) had negative blood cultures after 48 h of combination therapy.	[63]
	2012, Taiwan	Tang	Vancomycin, teicoplanin	8	Methicillin resistance (100%)	2 (6%)	VAN: 0%; TEC: 0%	in vitro (TK)	VAN: 8 (100%)	0%	TEC: 8 (100%)	0%	0%	No resistant isolates	Synergistic concentrations were 64 mg/L for FOS and 2 mg/L for VAN, at 24 h. Indifference was detected with 8 mg/L for TEC at 24 h. Significant reduction of colony count in biofilm model when FOS was in combination with either VAN and TEC after 5 days.	[69]
	2011, Taiwan	Tang	Vancomycin	5	Methicillin resistance (100%)	0%	0%	in vitro (TK)	5 (100%)	0%	0%	0%	No resistant isolates	No resistant isolates	All strains had borderline MIC values for VAN (2 mg/L). In vitro synergistic concentrations were 2 mg/L for VAN and 64 mg/L for FOS.	[175]
	2010, Spain	Pachon-Ibanez	Vancomycin	1	hGISA (100%)	0%	0%	in vitro (TK); in vivo (*mouse, peritonitis*)	1 (100%)	0%	0%	0%	No resistant isolate	No resistant isolate	Resistant (4 mg/L) sub-population frequency: 3.6 × 10^−6^ CFU/mL; in vitro synergistic concentrations were 1–2–4 mg/L for FOS and 1–2 mg/L for VAN at 24 h. In vivo combination was significant and effective in reducing bacteremia rates in 57% (n = 8 out of 14) of mice treated.	[36]
	2005, Italy	Pistella	Vancomycin, teicoplanin	7	Methicillin resistance (100%)	5 (71%)	VAN: 3 (42%); TEC: 6 (85.7%)	in vitro (TK)	VAN: 7 (100%); TEC: 0%	VAN: 0%; TEC: 7 (100%)	0%	0%	7 (100%)	0%	Synergistic concentrations were 8 mg/L for FOS and 1 × MIC for VAN (1, 2 or 4 mg/L respectively) at 24 h.	[176]
	1987, Spain	Rodriguez	Vancomycin	1	Methicillin resistance (100%)	0%	0%	in vitro (TK); in vivo (*rabbit*, endocarditis)	1 (100%)	0%	0%	0%	No resistant isolates	No resistant isolates	In vitro synergism at 24 and 48 h. Fixed concentrations of FOS at 8 mg/L and VAN at 1 mg/L. In vivo combination was successful in 10 rabbits (100%) showing sterile vegetations.	[61]
	1985, Spain	Alvarez	Vancomycin	148	Methicillin resistance (100%)	15 (10%)	1 (1%)	in vitro (CB)	0%	0%	145 (98%)	3 (2%)	-	-	1 strain was resistant to VAN (MIC > 32 mg/L).	[12]
*S. aureus, S. epidermidis*	2014, China	Shi	Vancomycin	3 (2 *S. aureus*, 1 *S. epidermidis*)	Methicillin resistance (67%)	3 (100%)	0%	in vitro (TK); in vivo (biofilm in rats’ tissues)	3 (100%)	0%	0%	0%	0%	No resistant isolates	In vitro synergistic concentrations at 1 mg/L for VAN and 64 mg/L for FOS at 6h and 24 h. In vivo significative reduction of biofilm formation in rats’ tissues (4, 100%).	[62]
	2001, Austria	Grif	Vancomycin	7 (5 *S. aureus*; 2 *S. epidermidis*)	*S. aureus*: GISA 1 (20%), MRSA 1 (20%)	-	0%	in vitro (CB, TK)	0%	0%	CB: *S. epidermidis* 2 (100%); *S. aureus* 5 (71%)	CB: *S. epidermidis* 0%; *S. aureus* 2 (28%)	-	-	TK showed indifference for all strains, with fixed concentration of FOS at 40 mg/L and VAN at 10 mg/L.	[43]
	1989, Germany	Gatermann	Vancomycin	33 (15 *S. aureus*; 18 *S. epidermidis*)	-	-	-	in vitro (CB)	*S. aureus*: 1 (6%); *S. epidermidis*: 1 (5%)	*S. aureus*: 8 (53%); *S. epidermidis*: 7 (39%)	*S. aureus*: 6 (40%); *S. epidermidis*: 9 (50%)	*S. aureus*: 0%; *S. epidermidis*: 1 (5%)	-	-	Synergistic concentrations not specified.	[177]
*E. faecalis, E. faecium*, *S. aureus*, *S. epidermidis*, CONS	1986, Italy	Debbia	Teicoplanin	76 strains: 30 *E. faecalis*, 6 *E. faecium*, 20 *S. aureus*, 10 *S. epidermidis*, 10 CoNS	Methicillin resistance (50% of *S. aureus*)	-	-	in vitro (CB, TK)	CB: 20 (67%) *E. faecalis*; 4 (67%) *E. faecium*; 6 (60%) *S. aureus*; 6 (60%) MRSA; 1 (10%) *S. epidermidis*; 6 (60%) CONS	CB: 10 (33%) *E. faecalis*; 2 (33%) *E. faecium*; 4 (40%) *S. aureus*; 4 (40%) MRSA; 9 (90%) *S. epidermidis*; 4 (40%) CONS	0%	0%	-	-	Synergistic concentrations not specified. 46 strains were tested also by TK. TK results-Synergism: 11 (92%) *E. faecalis*; 4 (100%) *E. faecium*; 6 (100%) *S. aureus*; 8 (100%) MRSA; 6 (75%) *S. epidermidis*; 8 (100%) CoNS. Additive effect: 1 (8%) *E. faecalis*; 2 (25%) *S. epidermidis*.	[178]
*S. pneumoniae*	2006, Spain	Ribes	Vancomycin	2	Resistance to penicillin (50%) and ceftriaxone (100%)	0%	0%	in vitro (TK); in vivo (rabbit, menigitis)	1 (50%)	1 (50%)	0%	0%	No resistant isolates	No resistant isolates	In vitro synergism at 24 h, at concentrations achievable in CSF. In vivo combination significant and effective in eradicating meningitis with sterile blood cultures (8, 100%).	[24]
	1994, France	Doit	Vancomycin	26	Isolates not susceptible to penicillin (100%)	0%	0%	in vitro (TK)	0%	0%	100%	0%	No resistant isolates	No resistant isolates	Fixed concentrations of FOS at 40 mg/L and VAN at 3 mg/L.	[134]
*S. epidermidis*	1990, France	Gaillanrd	Vancomycin	1	-	0%	0%	in vitro (TK)	1 (100%)	0%	0%	0%	No resistant isolates	No resistant isolates	Synergism at 4 h. Fixed concentrations of FOS at 12.5 mg/L and VAN at 7.5 mg/L. Effective to reduce biofilm formation (1; 100%).	[179]
	1990, Germany	Simon	Vancomycin, teicoplanin	20	Methicillin resistant (100%)	10 (50%)	VAN: 0%; TEC: 2 (10%)	in vitro (CB)	VAN: 4 (20%); TEC: 9 (45%)	VAN: 5 (25%); TEC: 6 (30%)	VAN: 11 (55%); TEC: 5 (25%)	VAN: 0%; TEC: 0%	-	VAN: no resistant isolates; TEC: NS	Synergistic concentrations at 0.5 X MIC for FOS, TEC and VAN. Good efficacy in artificial biofilm model when isolates were fully susceptible to FOS.	[180]
*E. faecalis - E. faecium*	2013, Taiwan	Tang	Vancomycin, teicoplanin	19 strains: 9 *E. faecalis*; 10 *E. faecium*	Vancomycin resistant (100%)	5 (55%) *E. faecalis*; 7 (70%) *E. faecium*	VAN: 19 (100%) both; TEC: 1 (11%) *E. faecalis*; 6 (60%) *E. faecium*	in vitro (TK)	VAN: 3 (33%) *E. faecalis*, 3 (30%) *E. faecium*; TEC: 8 (89%) *E. faecalis*, 3 (30%) *E. faecium*	0%	VAN: 6 (67%) *E. faecalis*, 7 (70%) *E. faecium*; TEC: 1 (11%) *E. faecalis*, 7 (70%) *E. faecium*	0%	0%	VAN: 3 (33%) *E. faecalis*; 3 (30%) *E. faecium*; TEC: 0%	Synergistic concentrations were 64 mg/L for FOS, 4 mg/L for VAN and 8 mg/L for TEC, at 24 h. FOS-TEC had synergistic effect against biofilm-producing *E. faecalis* (4; 44%) and one *E. faecium* (1; 10%) isolates. FOS-VAN had synergistic effect against only one biofilm-producing *E. faecalis* isolate (1; 11%).	[13]

**Table 9:** Studies on combination between fosfomycin and tetracyclines. CB: checkerboard assay; TK: time–kill assay; ET: E-test.

Strain	Year and Country	Author	Tetracycline	Number of Isolates	Known Resistance Mechanisms or Determinants (%)	FOS-Resistant (%)	Tetracycline-Resistant (%)	In Vitro (Methods)/In Vivo (Animal and site of Infection)	Synergistic Effect (%)	Additive Effect (%)	Indifferent Effect (%)	Antagonistic Effect (%)	FOS Susceptibility Restoration (%)	Tetracycline Susceptibility Restoration (%)	Comments	Reference
Enterobacterales	2019, USA	Flamm	Minocycline	20	7/30 MDR strains (*A. baumannii*, *Enterobacterales* e *P. aeruginosa*) included 2 ESBL e 2 KPC *Enterobacterales*	-	-	in vitro (CB)	4 (20%)	13 (65%)	1 (5%)	0%	-	-	Authors considered Partial Sinergy when FICI was between 0.5–1 and Additive effect for FICI = 1. Results for 2/20 strains (10%) were indeterminate.	[38]
	1977, Spain	Daza	Tetracycline	100	-	100 (100%)	-	in vitro (CB)	2 (2%)	-	98%	0%	-	-	Authors considered Synergistic effect when MIC was at least fourfold lower over initial MIC.	[66]
*P. aeruginosa*	2019, USA	Flamm	Minocycline	5	7/30 MDR strains (*A. baumannii*, *Enterobacterales* e *P. aeruginosa*)	-	-	in vitro (CB)	2 (40%)	3 (60%)	0%	0%	-	-	Authors considered Partial Sinergy when FICI was between 0.5–1 and Additive effect for FICI = 1.	[38]
*A. baumannii*	2013, China	Zhang	Minocycline	25	Pan-Drug-Resistant (100%)	100%	See Comments	in vitro (CB)	12%	56%	32%	0%	0%	100%	Mean MIC for Minocycline was 16, MIC range 4-16. Authors used CLSI breakpoint for MIN (S ≤ 4 mg/L).	[65]
*S. aureus*	2012, Taiwan	Tang	Minocycline	33 (8 TK)	MRSA (100%)	6%	61%	in vitro (TK, Biofilm MTT-staining method)	-	-	-	-	-	-	Only 8 strains were tested with TK. Biofilm cultures were 94% MIN resistant and 94% FOS resistant. Cases of synergism were observed with FOS+MIN combination. Percentages or other data were not reported by authors. Combination of FOS + MIN determined a statistically significant reduction on ODRs in biofilm cultures compared to single drugs.	[69]
	2011, China	Sun	Minocycline	87	MRSA (100%)	35 (40%)	13 (14%)	in vitro (CB)	76 (87%)	-	11 (12%)	0%	100%	92%	Authors considered Indifferent effect for FICI between 0,5 and 4. CLSI breakpoint was used for MIN (S ≤ 4 mg/L) and *E. faecalis* FOS breakpoint as presumptive breakpoint for MRSA (S ≤ 64 mg/L).	[70]
	2003, Japan	Nakazawa	Minocycline	32	MRSA (100%)	29 (91%)	26 (81%)	in vitro (Efficacy Time Index)	10 (31%)	1 (3%)	21 (65%)	-	-	-	-	[18]
*E. faecalis*	2013, Taiwan	Tang	Minocycline	9	VRE (100%)	56%	89%	in vitro (TK, Biofilm Model)	TKA: 2 (22%); BM: 1 (11%)	-	-	-	-	-	Additive, Indifferent and antagonistic effect were not evaluated.	[13]
*E. faecium*	2013, Taiwan	Tang	Minocycline	10	VRE (100%)	70%	80%	in vitro (TK, Biofilm Model)	TKA: 4 (40%); BM: 1 (10%)	-	-	-	-	-	Additive, Indifferent and antagonistic effect were not evaluated.	[13]
	2012, USA	Descourouez	Minocycline	32	VRE (100%)	9%	See Comments	in vitro (TK)	0%	0%	100%	0%	-	-	The authors considered MIC ≤ 64 mg/L as FOS breakpoint. Most of strains were minocycline resistant (MIC range 4–32, mean MIC 16 mg/L).	[67]
	2019, USA	Davis	Doxycycline	24	VRE (100%)	96%	8%	in vitro (ET, TK)	CK: 11 (46%); TK: 10 (41%)	CK: 13 (54%); TK: 4 (16%)	CK: 0%; TK: 10 (41%)	CK: 0%; TK 0%	-	-	Authors used CLSI breakpoint for DOX (S ≤ 4 mg/L) and *E. faecalis* FOS breakpoint as presumptive breakpoint for *E. faecium* (S ≤ 64 mg/L).	[68]
*N. gonorrhoeae*	2015, Netherlands	Wind	Minocycline	4	Azithromycin and Ceftriaxone Resistant (100%)	-	-	in vitro (ET)	0%	0%	4 (100%)	-	-	-	-	[54]

**Table 10:** Studies on combination between fosfomycin and polymyxins. CB: checkerboard assay; TK: time–kill assay; ET: E-test.

Strain	Year and Country	Author	Polymyxin	Number of Isolates	Known Resistance Mechanisms or Determinants (%)	FOS-Resistant (%)	Polymyxin-ResIstant (%)	In Vitro (Methods)/In Vivo (Animal and Site of Infection)	Synergistic Effect (%)	Additive Effect (%)	Indifferent Effect (%)	Antagonistic Effect (%)	FOS Susceptibility Restoration (%)	Polymyxin Susceptibility Restoration (%)	Comments	Reference
Enterobacterales	2019, USA	Flamm	Colistin	20	carbapenem-resistant (5%), KPC (10%), ESBL (10%)	-	-	in vitro (CB, TK)	1 (5%)	5 (25%)	8 (40%)	0%	-	-	For 6 isolates the effect of the combination was indeterminate.	[38]
	2015, UK	Albur	Colistin	6	NDM-1 (100%)	3 (50%)	0%	in vitro (TK)	3 (50%)	0%	3 (50%)	0%	-	-	The combination was synergistic against FOS-S isolates. Against FOS-R isolates, an additive effect was observed after 12h, but then regrowth occurred.	[181]
*E. coli*	2013, Switzerland	Corvec	Colistin	1	CTX-M15, ESBL (100%)	0%	0%	in vitro (TK), in vivo (foreign-body infection model)	1 (100%)	0%	0%	0%	-	-	-	[73]
	2011, France	Berçot	Colistin	1	NDM-1	0%	0%	in vitro (CB, TK)	0%	1 (100%)	0%	0%	-	-	*E. coli* J53	[85]
	2011, Greece	Samonis	Colistin	20	ESBL (100%)	0%	0%	in vitro (ET)	3 (15%)	-	-	0%	-	-	-	[86]
*E. cloacae*	2011, France	Berçot	Colistin	2	NMD-1	1 (50%)	0%	in vitro (CB, TK)	0%	2 (100%)	0%	0%	-	-	-	[85]
*K. pneumoniae*	2020, Turkey	Buket Erturk Sengel	Colistin	17	KPC (OXA-48, NDM) (100%)	41%	65%	in vitro (CB, TK)	7 (41%)	3 (18%)	5 (29%)	2 (12%)	-	-	-	[142]
	2019, India	Bakthavatchalam	Colistin	50	CR-Kp, NDM, OXA-43 (100%)	24 (48%)	14 (30%)	in vitro (TK)	8 (16%)	0%	42 (84%)	0%	-	-	-	[141]
	2020, Sweden	Wistrand-Yuen	Polymyxin B	5 (4 used for FOS+PMB)	KPC-2, KPC-3, NMD-1, OXA-48, VIM-1 (100%)	3 (60%)	2 (40%)	in vitro (TK)	5 (31%)	2 (12%)	-	-	-	-	Synergistic rate inferred from 4 isolates monitored at different times. If evaluated only after 24 h, syn: 40%; add 20%.	[182]
	2019, France	Crémieux	Colistin	1	carbapenem-resistant (100%)	0%	0%	in vitro (TK); in vivo (rabbit, osteomyelitis)	1 (100%)	0%	0%	0%	-	-	-	[71]
	2018, China	Wang	Colistin	4	carbapenem-resistant (100%)	2 (50%)	0% (75% heteroresistant)	in vitro (TK)	31 (43%)	8 (11%)	33 (46%)	0%	-	-	3 isolates showed heteroresistance: the total number of experiments was 72 (3 different colistin concentrations tested in 6 different times).	[183]
	2018, China	Yu	Colistin	3	KPC (100%)	1 (33%)	3 (100%)	in vitro (TK)	2 (66%)	1 (33%)	0%	0%	-	-	-	[164]
	2017, Taiwan	Ku	Colistin	9	ESBL-producing KP (5/9 carbapenem-R, 4/9 carbapenem-S)	4 (45%)	1 (11%)	in vitro (TK)	5 (55%)	0%	4 (45%)	0%	-	-	-	[84]
	2017, China	Yu	Colistin	3	KPC2 (100%)	0%	1 (33%)	in vitro (TK)	3 (100%)	0%	0%	0%	-	-	-	[50]
	2017, China	Yu	Colistin	136	KPC-Kp (100%)	78 (57%)	1 (1%)	in vitro (CB, TK)	5 (3%)	109 (80%)	22 (16%)	0%	-	-	-	[89]
	2018, USA	Bulman	Polymyxin B	2	KPC-2 (100%)	0%	0%	in vitro (TK); in vivo (hollow-fibre infection model)	2 (100%)	-	-	-	-	-	-	[75]
	2014, Sweden	Tängdeén	Colistin	4	VIM (50%), NDM (50%)	2 (50%)	0%	in vitro (TK)	3 (75%)	0%	1 (25%)	0%	-	-	Synergism in 1 VIM- and 2 NDM-producing isolates, although NDM-producing isolates were FOS-R.	[146]
	2013, Turkey	Evren	Colistin	12	OXA-48 (100%)	11 (92%)	2 (17%)	in vitro (CB)	0%	0%	0%	12 (100%)	-	-	-	[74]
	2011, France	Berçot	Colistin	3	NDM-1 (100%)	0%	0%	in vitro (CB, TK)	0%	1 (33%)	2 (66%)	0%	-	-	-	[85]
	2011, Greece	Samonis	Colistin	50	carbapenem-resistant (100%)	3%	25%	in vitro (ET)	18 (36%)	-	-	0%	-	-	-	[86]
	2011, Greece	Samonis	Colistin	14	ESBL (100%)	3%	25%	in vitro (ET)	1 (7%)	-	-	0%	-	-	-	[86]
	2011, Greece	Souli	Colistin	17	KPC-2 (100%)	4 (23%)	7 (41%)	in vitro (TK)	2 (12%)	0%	15 (88%)	0%	-	-	-	[53]
*K. oxytoca*	2011, France	Berçot	Colistin	1	NDM-1	0%	0%	in vitro (CB, TK)	0%	100%	0%	0%	-	-	-	[85]
*P. rettgeri*	2011, France	Berçot	Colistin	1	NDM-1	0%	100%	in vitro (CB, TK)	0%	0%	100%	0%	-	-	-	[85]
*P. aeruginosa*	2019, USA	Flamm	Colistin	5	-	-	-	in vitro (CB, TK)	0%	1 (20%)	4 (80%)	0%	-	-	-	[38]
	2016, Australia	Walsh	Polymyxin B	4	MDR (75%)	50%	50%	in vitro (TK)	19 (18%)	27 (25%)	-	-	-	-	FOS in combination with polymyxin B increased bacterial killing, but did not suppress emergence of FOS resistance. The total number of experiments was 108 (9 combinations of FOS + CIP at different concentrations, in 3 different times).	[76]
	2011, Greece	Samonis	Colistin	15	MDR (100%)	6%	0%	in vitro (ET)	2 (13%)	-	-	0%	-	-	-	[86]
	2015, China	Di	Colistin	87	CRPA (100%)	75%	4% (5/87)	in vitro (CB, TK)	19 (21%)	29 (33)%	39 (44%)	0%	-	3 (60%)	-	[184]
*A. baumannii-A. calcoaceticus spp. Complex*	2019, USA	Flamm	Colistin	5	MDR (20%)	-	-	in vitro (CB, TK)	2 (40%)	1 (20%)	1 (20%)	0%	-	-	For 1 isolate the effect of the combination was indeterminate.	[38]
*A. baumannii*	2020, South Korea	Su Ku	Colistin	1	OXA-23 (100%)	100%	0%	in vitro (TK); in vivo (mouse, nasal inoculation)	1 (100%)	0%	0%	0%	-	-	-	[72]
	2019, Turkey	Sertcelik	Colistin	23	carbapenem-resistant (100%)	100%	26%	in vitro (CB)	1 (4%)	10 (43%)	12 (52%)	0%	-	-	-	[185]
	2019, China	Bian	Colistin	9	carbapenem-resistant (100%)	-	0%	in vitro (CB, TK)	1 (11%)	-	-	-	-	-	-	[186]
	2018, China	Zhu	Colistin	21	-	100%	61% (13/21)	in vitro (CB)	0%	2 (9%)	19 (90%)	0%	-	-	The authors reported 8 isolates to be colistin-R, but only 3 isolates had MIC > 2.	[151]
	2018, Thailand	Leelasupasri	Colistin	15	carbapenem-resistant (100%)	100%	0%	in vitro (CB, ET)	4 (26%)	7 (46%)	4 (26%)	0%	-	-	-	[187]
	2017, Thailand	Lertsrisatit	Colistin	17	CoR-AB; carbapenemase-producing; efflux-pump (100%)	100%	100%	in vitro (CB, ET)	-	-	-	0%	-	-	Treatment in vivo (patients) with COL + FOS lead to death (2/2).	[188]
	2016, China	Fan	Colistin	12	XDR (100%)	100%	0%	in vivo (mouse, thigh-infection)model	1 (8%)	-	-	0%	-	-	-	[189]
	2016, Brazil	Leite	Colistin	20	OXA-23, OXA-143 (100%)	100%	35% (7/20)	in vitro (CB, TK, 2-well)	0%	-	-	-	-	-	-	[83]
	2015, China	Wei	Colistin	50	XDR (100%)	94%	50%	in vitro (CB)	25 (50%)	0%	22 (44%)	3 (6%)			Synergism (FICI: =< 0.5). Indifference (FICI: 0.5–4). Antagonism (FICI: >= 4).	[190]
	2013, China	Zhang	Polymyxin B	25	PDR (100%)	100%	100%	in vitro (TK)	4 (16%)	11 (44%)	10 (40%)	0%	0%	25 (100%)	-	[65]
	2011, Thailand	Santimaleeworagun	Colistin	8	carbapenem-resistant (100%)	0%	-	in vitro (CB, TK)	13%	-	-	-	-	-	-	[99]
*N. gonorrhoeae*	2014, Netherlands	Wind	Colistin	4	-	-	-	in vitro (ET)	0%	-	-	-	-	-	-	[54]

**Table 11:** Studies on combination between fosfomycin and daptomycin. CB: checkerboard assay; TK: time–kill assay; ET: E-test.

Strains	Year and Country	Author	Number of Isolates	Known Resistance Mechanisms or Determinants (%)	FOS-Resistant (%)	Daptomycin-Resistant (%)	In Vitro (Methods)/In Vivo (Animal and Site of Infection)	Synergistic Effect (%)	Additive Effect (%)	Indifferent Effect (%)	Antagonistic Effect (%)	FOS Susceptibility Restoration (%)	Daptomycin Susceptibility Restoration (%)	Comments	Reference
*S. aureus*	2019, Taiwan	Lee	100	MRSA (100%)	15 (15%)	0%	in vitro (CB)	37 (37%)	44 (44%)	19 (19%)	0%	-	-	All isolates had MIC daptomycin = 1 (previously selected among 1353 isolates).	[191]
	2019, Spain	Coronado-Alvarez	4	MRSA (50%)	-	-	in vitro (TK)	4 (100%)	0%	0%	0%	-	-	The authors also performed a retrospective review of 75 patients with severe Gram-positive infections and found that DAP + FOS (30) was the most effective combination.	[63]
	2018, Spain	Garcìa-de-la-Mària	5 (in vitro); 1 (in vivo)	MRSA (100%)	0%	0%	in vitro (TK), in vivo (rabbit, endocarditis)	in vitro: 5 (100%); in vivo: 1 (100%)	0%	0%	0%	-	-	-	[79]
	2017, Turkey	Aktas	25	MRSA (100%)	11 (44%)	0%	in vitro (CB)	25 (100%)	0%	0%	0%	-	-	-	[80]
	2015, Austria	Lingscheid	1	MRSA (100%)	0%	0%	in vivo (rats, implant-associated osteomyelitis)	1 (100%)	0%	0%	0%	-	-	-	[81]
	2013, Spain	Garrigós	1	MRSA (100%)	0%	0%	in vitro (TK), in vivo (rat, foreign-body infection)	in vitro: 0%; in vivo: 1 (100%)	0%	in vitro: 1 (100%)	0%	-	-	-	[37]
	2012, Spain	Miró	14	MRSA (35%); GISA (14%)	0%	1 (7%)	in vitro (TK)	11 (79%)	3 (21%)	0%	0%	-	-	The combination was bactericidal against 8 (57%) isolates. The authors also reported the case reports of 3 patients with *S. aureus* (1 MSSA, 2 MRSA) endocarditis successfully treated with high-dose DAP (10/kg/day) + FOS.	[192]
	2011, Austria	Poeppl	1	MRSA (100%)	0%	0%	in vivo (rats, osteomyelitis)	0%	0%	1 (100%)	0%	-	-	FOS and FOS + DAP were significantly superior to placebo and to DAP alone. FOS + DAP was not more effective than FOS alone.	[193]
*E. faecalis*	2019, China	Zheng	4 (TK) + 4 (biofilm assay)	-	1 (12%)	2 (25%)	in vitro (TK, biofilm assay)	TK: 4 (100%). Biofilm assay: 3 (75%)	0%	TK: 0%. Biofilm assay: 1 (25%)	0%	-	-	TK performed on 4 linezolid-R isolates. Biofilm assay performed on 4 linezolid-S isolates. DAP + FOS demonstrated significantly more anti-biofilm activities then DAP or FOS alone.	[194]
	1992, USA	Rice	1	-	0	1 (100%)	in vitro (TK), in vivo (rats, endocarditis)	in vitro: 1 (100%)	in vitro: 0%; in vivo: 1 (100%)	0%	0%	-	in vitro: 1 (100%)	The isolate was highly R to gentamicin. DAP + FOS sterilized more valves (59% VS 35%) than DAP alone. Despite this, the combination in vivo was considered "additive" because it was not possible to demonstrate a statistically significant superiority in comparison with DAP alone.	[82]
	1989, USA	Rice	21	-	0	0	in vitro (TK)	21 (100%)	0%	0%	0%	-	-	All isolates were highly R to gentamicin. The bactericidal effect of DAP alone was not increased by the addition of FOS.	[195]
*E. faecium*	2013, USA	Descourouez	4	VRE (100%)	0%	0%	in vitro (TK)	4 (100%)	0%	0%	0% [196]	-	-	The combination resulted strongly bactericidal.	[67]
*Staphylococcus* spp., *Enterococcus* spp.	1988, Italy	Debbia	50	-	-		in vitro (CB, TK)	CB: 80%TK: 95%	0%	CB: 20%TK: 5%	0%	-	-	A total of 50 strains was tested with CB, and only 20 strains were tested with TK.	[197]

**Table 12:** Studies on combination between fosfomycin and tigecycline. CB: checkerboard assay; TK: time–kill assay; ET: E-test.

Strain	Year and Country	Author	Number of Isolates	Known Resistance Mechanisms or Determinants (%)	FOS-Resistant (%)	Tigecycline-Resistant (%)	In Vitro (Methods)/In Vivo (Animal and Site of Infection)	Synergistic Effect (%)	Additive Effect (%)	Indifferent Effect (%)	Antagonistic Effect (%)	FOS Susceptibility Restoration (%)	Tigecycline Susceptibility Restoration (%)	Comments	Reference
Enterobacterales	2019, USA	Flamm	20	7/30 MDR strains (*A. baumannii*, Enterobacterales e *P. aeruginosa*) included 2 ESBL e 2 KPC Enterobacterales	-	-	in vitro (CB)	5 (25%)	10 (50%)	5 (25%)	0%	-	-	Authors considered Partial Sinergy when FICI was between 0.5–1 and Additive effect for FICI = 1.	[38]
	2017, Taiwan	Ku	9	ESBL KP producing (100%)	4 (44,4%)	4 (44%)	in vitro (TK)	6 (66%)	0%	3 (33%)	0%	-	-	-	[84]
	2011, France	Berçot	9	NDM-1 KPC (100%)	2 (22%)	3 (33%)	in vitro (CB)	0%	-	9 (100%)	0%	-	-	Authors considered Indifferent effect for FICI between 0.5 and 4.	[85]
*E. coli*	2013, Switzerland	Corvec	1	Bj HDE-1 (100%) (ESBL and Ciprofloxacin resistant)	0%	0%	in vitro (TK); in vivo (Guinea pigs, cage infection)	TK: 0%; in vivo: 0%	TK: 100%; in vivo: 0%	TK: 0%; in vivo: 100%	0%	-	-	-	[73]
	2011, Greece	Samonis	20	ESBL (100%)	0%	1 (5%)	in vitro (ET)	5 (25%)	-	15 (75%)	0%	-	-	Authors considered Indifferent effect for FICI between 0.5 and 4. In vivo experiment: bacterial count using FOS + TIG combination was reduced ≥ 2 log over single antimicrobials	[86]
*K. pneumoniae*	2019, China	Huang	30	KPC (100%)	19 (63%)	11 (36%)	in vitro (ET, CB)	ET: 5 (16%); CK: 4 (13%)	ET: 9 (30%); CK: 11 (36%)	ETt: 16 (53%); CK: 15 (50%)	0%	ET: 14/19 (73%); CK: 6/15 (40%)	ET: 5/11 (45%); CK: 7/13 (53,%)	ET and CB showed different rates of FOS and TIG resistance and different rates of susceptibility restoration; otherwise the 2 methods had similar resulted in establishing synergistic, additive or indifferent effect.	[88]
	2019, Greece	Papoutsaki	11	KPC (100%)	35%	96%	in vitro (ET, TK)	ET: 16/33 (48%); TKA: 1/22 (4%)	ET: 17/33 (51%); TKA: 21/22 (95%)	0%	0%	-	-	ET was performed three times with different methods: a) Etest/Agar method; b) Cross formation method; c) MIC/MIC ratio method. TK was performed two times: a) TIG 1,3 mg/L + FOS 0,5xMIC and b) TIG 1,3 mg/L + FOS 30 mg/L.	[87]
	2017, China	Yu	136	KPC (100%)	78 (57%)	25 (18%)	in vitro (CB, TK)	CK: 2 (1%); TKA: 0%	CK: 113 (83%); TKA: 3 (75%)	CK: 19 (14%); TKA: 1 (25%)	CK: 2 (1%); TKA: 0%	-	-	Only 4 strains were tested with TK.	[89]
	2013, Turkey	Evren	12	OXA-48 (100%)	11 (92%)	5 (41%)	in vitro (CB)	4 (33%)	6 (50%)	2 (16%)	0%	-	-	Authors considered Indifferent effect for FICI between 0.5 and 4. In vivo experiment: bacterial count using FOS + TIG combination was reduced ≥ 2 log over single antimicrobials	[74]
	2011, Greece	Samonis	65	Serine-KPC (77%) - MBL (1%) - ESBL (21%)	1 (1%)	10 (15%)	in vitro (ET)	18 (27%)	-	47 (72%)	0%	-	-	Authors considered Indifferent effect for FICI between 0.5 and 4. In vivo experiment: bacterial count using FOS + TIG combination was reduced ≥ 2 log over single antimicrobials	[86]
*P. aeruginosa*	2011, Greece	Samonis	15	MDR (100%)	1 (6%)	15 (100%)	in vitro (ET)	2 (13%)	-	13 (86%)	0%	-	-	Authors considered Indifferent effect for FICI between 0.5 and 4. In vivo experiment: bacterial count using FOS + TIG combination was reduced ≥ 2 log over single antimicrobials	[86]
*A. baumannii*	2019, USA	Flamm	5	7/30 MDR strains (*A. baumannii*, Enterobacterales e *P. aeruginosa*)	-	-	in vitro (CB)	0%	4 (80%)	1 (20%)	0%	-	-	Authors considered Partial Sinergy when FICI was between 0.5–1 and Additive effect for FICI = 1.	[38]
	2016, Netherlands	Leite	20	Colistin-Resistant (65%)	20 (100%)	5%	in vitro (CB, 2-Well Method)	0%	-	-	-	-	-	Any synergistic effect was reported. Additive, Indifferent and antagonistic effect were not evaluated.	[83]
*S. aureus*	2018, Italy	Simonetti	15	MRSA (100%)	0	0%	in vitro (CB); in vivo (mice, wound infection)	12 (80%)	-	3 (20%)	0%	-	-	Authors considered Indifferent effect for FICI between 0.5 and 4. In vivo experiment: bacterial count using FOS + TIG combination was reduced ≥ 2 log over single antimicrobials.	[90]
	2012, Taiwan	Tang	33 (8 TK)	MRSA (100%)	6%	0%	in vitro (TK, Biofilm MTT-staining method)	0%	-	100%	0%	-	-	Only 8 strains were tested with Time–kill Assay. Biofilm cultures were 100% TIG resistant and 94% FOS resistant. No FICI were reported by authors, no synergistic effect was seen on any strains.	[69]
*E. faecalis*	2018, Italy	Simonetti	15	-	0%	0%	in vitro (CB); in vivo (mice, wound infection)	12 (80%)	-	3 (20%)	0%	-	-	Authors considered Indifferent effect for FICI between 0.5 and 4. In vivo experiment: bacterial count using FOS + TIG combination was reduced ≥ 2 log over single antimicrobials.	[90]
	2013, Taiwan	Tang	9	VRE (100%)	56%	0%	in vitro (TK, Biofilm Model)	TKA: 3 (33%); BM: 5 (56%)	-	-	-	-	-	Additive, Indifferent and antagonistic effect were not evaluated.	[13]
*E. faecium*	2019, Thailand	Hemapampairoa	12	VRE (100%)	12 (100%)	3 (25%)	in vitro (CB)	1 (8%)	9 (75%)	2 (16)%	0%	-	-	-	[55]
	2018, Italy	Simonetti	15	-	0%	0%	in vitro (CB)	10 (66)%	-	5 (33%)	0%	-	-	Authors considered Indifferent effect for FICI between 0.5 and 4.	[90]
	2013, Taiwan	Tang	10	VRE (100%)	70%	0%	in vitro (TK, Biofilm Model)	TKA: 3 (30%); BM: 1 (10%)	-	-	-	-	-	Additive, Indifferent and antagonistic effect were not evaluated.	[13]
*N. gonorrhoeae*	2015, Netherlands	Wind	4	Azithromycin and Ceftriaxone Resistant (100%)	-	-	in vitro (ET)	0%	0%	4 (100%)	-	-	-	-	[54]

**Table 13:** Studies on combination between fosfomycin and linezolid. CB: checkerboard assay; TK: time–kill assay; ET: E-test.

Strain	Year and Country	Author	Number of Isolates	Known Resistance Mechanisms or Determinants (%)	FOS-Resistant (%)	Linezolid-Resistant (%)	In Vitro (Methods)/In Vivo (Animal and Site of Infection)	Synergistic Effect (%)	Additive Effect (%)	Indifferent Effect (%)	Antagonistic Effect (%)	FOS Susceptibility Restoration (%)	Linezolid Susceptibility Restoration (%)	Comments	Reference
*S. aureus*	2018, China	Chen	11 (3 TK)	MRSA (50%)	0%	0%	in vitro (CB, TK)	CK: 8 (72%); TK: 3 (100%)	CK: 3 (27%); TK: 0%	CK: 0%; TK: 0%	CK: 0%; TK: 0%	-	-	Only 3 strains were tested with TK. For the same 3 strains, the authors also evaluated. Post-Antibiotic Effect (PAE) of LZD alone and in combination with FOS. PAE of LZD + FOS seemed to be increased with the increase in time of exposure, even if no statistically significant difference was found.	[198]
	2018, Spain	Coronado-Alvarez	2	MRSA (100%)	-	-	in vitro (TK)	2 (100%)	0%	0%	0%	-	-	Synergy was defined as a reduction > 3 log CFU/mL over antimicrobial agent alone, additive effect was defined as areduction < 3 log CFU/mL. Synergistic effect was demonstrated only when 4 x MIC LZD + 2 x MIC FOS were used; 1 × MIC LZD + 2 × MIF FOS regimen showed Additive effect.	[63]
	2016, China	Chai	3 (1 TK)	MRSA (100%)	2 (66%)	0%	in vitro (CB, TK)	CK: 3 (100%); TK: 1 (100%)	CK: 0%; TK: 0%	CK: 0%; TK: 0%	CK: 0%; TK: 0%	-	-	Only 1 strain was tested with Time–kill Assay. The authors also evaluated in vitro and in vivo efficacy of LIN + FOS on MRSA biofilm (all 3 strains), demonstrating a synergistic effect only in vitro when using 1/2 MIC LZD + 1/2 MIC FOS and not with lower concentrations.	[94]
	2014, China	Xu-Hong	102	MRSA (100%)	**MIC range 16-128 mg/L	0%	in vitro (CB)	100 (98%)	-	2 (2%)	0%	100%	100%	The authors considered Indifferent effect for FICI between 0.5 and 4. Fosfomycin MIC range in combination was 2-32 mg/L, LZD MIC in combination was 0,125–1 mg/L.	[199]
	2012, Taiwan	Tang	33 (8 TK)	MRSA (100%)	6%	0%	in vitro (TK, Biofilm MTT-staining method)	-	-	-	-	-	-	Only 8 strains were tested with Time–kill Assay. Biofilm cultures were 100% LZD resistant and 94% FOS resistant. Combination of FOS + LZD determined a statistically significant reduction on ODRs in biofilm cultures.	[69]
	2010, Spain	Pachón-Ibáñez	1	GISA 100% (Gentamicin Intermediate *S. aureus*)	-	-	in vitro (TK); in vivo (Murine peritonitis model)	1 (100%)	0%	0%	0%	-	-	In vivo experiment on mice showed a higher rate of blood culture negativization when using FOS + LZD therapy (57%) then using FOS or LZD alone (43% and 27% respectively).	[36]
	2006, Spain	Sahuquillo Arce	5 (4 TK)	-	0%	0%	in vitro (CB, TK)	CK: 4 (80%); TK: 4 (100%)	CK: 1 (20%); TK: 0%	CK: 0%; TK: 0%	CK: 0%; TK: 0%	-	-	Synergistic effect at CB was confirmed with TK on 4 strains.	[200]
	2001, Austria	Grif	5 (1 TK)	MRSA (60%)	0%	0%	in vitro (CB, TK, TEM)	CK: 5 (100%); TK: 0%	-	CK: 0%; TK: (1) 100%	CK: 0%; TK: 0%	-	-	The authors did not consider additive effect. They also performed Transmission Electron Microscopy, demonstrating profound morphological alteration of 2 strains when using FOS + LZD, which were not seen using FOS or LZD alone.	[43]
	2018, China	Li	4	MRSA (50%)	0%	0%	in vitro (CB, TK); in vivo (*Galleria melonella* Survival Assay)	CK: 4 (100%); TK: 4 (100%)	CK: 0%; TK: 0%	CK: 0%; TK: 0%	CK: 0%; TK: 0%	-	-	TKA showed synergism, but bacteriostatic effect. In vivo experiment showed statistically significant higher efficacy of high-dose LZD + FOS combination, then high dose of FOS or LZD alone, but low-dose combination had no significant differences with monotherapy orhigh-dose combination.	[95]
*S. epidermidis*	2001, Austria	Grif	2	-	0%	0%	in vitro (CB)	2 (100%)	-	0%	0%	-	-	The authors did not consider additive effect. They also performed Transmission Electron Microscopy, demonstrating profound morphological alteration of 2 strains when using FOS + LZD, which were not seen using FOS or LZD alone.	[43]
*E. faecalis*	2013, Taiwan	Tang	9	VRE (100%)	56%	0%	in vitro (TK, Biofilm Model)	TKA: 0%; BM: 0%	-	-	-	-	-	The authors did not consider additive, indifferent or antagonistic effect.	[13]
	2019, China	Qi	2	VRE (50%)	2 (100%)	0%	in vitro (CB, TK, TEM)	CK: 0%; TK: 0%)	CK: 2 (100%); TK: 1 (50%)	CK: 0%; TK: 1 (50%)	CK: 0%; TK: 0%	2 (100%)	2 (100%)	Transmission Electron Microscopy, demonstrated more morphological alterations when using FOS + LZD, then using FOS or LZD alone.	[201]
*E. faecium*	2019, Thailand	Hemapampairoa	12	VRE (100%)	12 (100%)	0%	in vitro (CB)	3 (25%)	9 (75%)	0%	0%	-	-	-	[55]
	2013, Taiwan	Tang	10	VRE (100%)	70%	80%	in vitro (TK, Biofilm Model)	TKA: 1 (10%); BM: 0%	-	-	-	-	-	The authors did not consider additive, indifferent or antagonistic effect.	[13]
	2012, USA	Descourouez	32	VRE (100%)	9%	3%	in vitro (TK)	See comments	See comments	0%	0%	-	-	The authors considered MIC ≤ 64 mg/L as FOS breakpoint. FOS combined with LZD was either synergistic or additive yet bacteriostatic. Percentages of strains on which there was synergistic effect were not reported	[67]
	2019, China	Qi	4	VRE (75%)	4 (100%)	1 (25%)	in vitro (CB, TK, TEM); in vivo (Galleria Melonella Survival Assay)	CK: 2 (50%); TK: 2 (50%)	CK: 1 (25%); TK: 1 (25%)	CK: 1 (25%); TK: 1 (25%)	0%	3 (75%)	4 (100%)	Transmission Electron Microscopy, demonstrated more morphological alterations when using FOS + LZD, then using FOS or LZD alone. In vivo experiment showed higher survival rates of larvae when using FOS + LZD then LZD alone, but similar rates using FOS alone.	[201]

**Table 14:** Studies on combination between fosfomycin and rifampin. CB: checkerboard assay; TK: time–kill assay; ET: E-test.

Strain	Year and Country	Author	Number of Isolates	Known Resistance Mechanisms or Determinants (%)	FOS-Resistant (%)	Rifampin-Resistant (%)	In Vitro (Methods)/In Vivo (Animal and Site of Infection)	Synergistic Effect (%)	Additive Effect (%)	Indifferent Effect (%)	Antagonistic Effect (%)	FOS Susceptibility Restoration (%)	Rifampin Susceptibility Restoration (%)	Comments	Reference
*E. coli*	1978, Spain	Olay	17	-	-	-	in vitro (CB); in vivo (mouse, peritonitis)	1 (5,9%)	9 (52,9%)	7 (41,2%)	0%	-	-	-	[14]
*A. baumannii*	2016, Brazil	Leite	20	OXA-51, OXA-23, OXA-143 (100%)	20 (100%)	20 (100%)	in vitro (CB, TK)	0%	-	-	-	-	-	-	[83]
*S. aureus*	2018, Italy	Simonetti	16	MRSA (100%)	0%	2 (12%)	in vitro (CB, TK); in vivo (mouse, wound infection)	16 (100%)	0%	0%	0%	-	-	-	[90]
	2014, Switzerland	Mihailescu	1	MRSA (100%)	0%	0%	in vitro (ET, TK); in vivo (foreign-body infection model)	in vitro: 1 (100%); in vivo: 100% at day 12	0%	0%	0%	-	-	-	[96]
	2013, China	Tang	8	MRSA (100%)	0%	8 (100%)	in vitro (biofilm assay)	4 (50%)	-	-	-	-	-	-	[91]
	2012, Spain	Garrigos	1	MRSA (100%)	-	-	in vitro (TK); in vivo (rat, tissue cage infection)	in vivo: 1 (100%) at day 8 and day 11	-	-	in vitro: 1 (100%)	-	-	In vitro FOS antagonized the effect of RIF.	[37]
	2012, Taiwan	Tang	33	MRSA (100%)	6% (planktonic) 94% (biofilm)	0% (planktonic) 79% (biofilm)	in vitro (TK)	0%	-	-	-	-	-	-	[69]
	2001, Austria	Grif	5	MRSA (100%)	-	-	in vitro (CB, TK)	100%	-	-	-	-	-	-	[43]
	1987, France	Quentin	6	-	33%	0%	in vitro (TK)	0%	0%	33%	33%	-	-	RIF antagonizes FOS. In particular, it antagonizes FOS against susceptible and intermediate isolates to RIF. The combination resulted indifferent against RIF-resistant isolates. For 2 isolates it was not possible to infer their susceptibility to RIF.	[35]
	1984, Germany	Traub	6	GRMR (100%)	0%	0%	in vitro (CB); in vivo (mouse, peritonitis)	-	-	2 (33%)	-	-	-	-	[202]
	1978, Spain	Olay	38	-	-	-	in vitro (CB); in vivo (mouse, peritonitis)	13 (34%)	24 (63%)	1 (2%)	-	-	-	-	[14]
*S. pneumoniae*	1994, France	Doit	26	-	0%	0%	in vitro (TK)	0%	0%	100%	0%	-	-	-	[134]
*S. agalactiae, S. pyogenes, S. oralis*	2017, Germany	Gonzalez Moreno	3	-	33%	0%	in vitro (ET)	1 (100%) *S. oralis*	-	1 (100%) *S. agalactiae*; 1 (100%) *S. pyogenes*	-	-	-	-	[9]
*E. feacalis*	2018, Italy	Simonetti	16	-	0%	2 (12%)	in vitro (CB, TK); in vivo (mouse, wound infection)	12 (75%)	0%	4 (25%)*	0%	-	-	*The FICIs were interpreted as indifferent if > 0.5 and < 4.	[90]
	2013, Taiwan	Tang	9	VRE (100%)	56%	11%	in vitro (TK, biofilm)	TK: 3 (33%); biofilm: 9 (100%)	-	-	0%	-	-	-	[13]
*E. faecium*	2018, Italy	Simonetti	15	-	0%	2 (13%)	in vitro (CB, TK); in vivo (mouse, wound infection)	11 (73%)	0%	4 (27%)*	0%	-	-	*The FICIs were interpreted as indifferent if > 0.5 and < 4.	[90]
	2013, Taiwan	Tang	10	VRE (100%)	70%	90%	in vitro (TK)	TK: 2 (20%); biofilm: 4 (40%)	-	-	-	-	-	-	[13]
*S. epidermidis*	2011, Austria	Grif	2	MRSA (100%)	-	-	in vitro (CB, TK)	2 (100%)	-	-	-	-	-	-	[43]
	1987, France	Quentin	3	-	NA	NA	in vitro (TK)	0%	0%	50%	-	-	-	For 1 isolate it was not possible to infer its susceptibility to RIF.	[35]
*N. gonorrhoeae*	2015, Netherlands	Wind	4	-	-	-	in vitro (ET)	1 (25%)	-	-	-	-	-	-	[54]

**Table 15:** Effect of FOS in combination with different antibiotics: overview.

Antibiotic Class	Strains	Number of Studies	Number of Isolates	Synergistic Effect (%)	Additive Effect (%)	Indifferent Effect (%)	Antagonistic Effect (%)	Comments
Penicillins, penicillins + β-lactamase inhibitors, penicillinase-resistant penicillins	Enterobacterales	9	267	51	19	28		One study [11] reported high rates of indifferent effect of FOS + PIP/TAZ against PIP/TAZ-R isolates.
	*P. aeruginosa*	6	235	15	40	45		-
	*Acinetobacter* spp.	1	5	60	20	0		-
	*Staphylococcus* spp.	7	295	42	15	33		-
	*Streptococcus* spp.	6	119	30	55	15		-
	*Enterococcus* spp.	4	60	25	0	42	10	Antagonistic effect observed in biofilms of some *E. faecalis* isolates.
Cephalosporins, cephalosporins + β-lactamase inhibitors	Enterobacterales	8	251	33	33	20		One study [11] reported high rates of indifferent effect of FOS + 4 different cephalosporins against cephalosporin-R isolates.
	*P. aeruginosa*	13	318	36	40	23	1	Antagonistic effect against 4 *P. aeruginosa* isolates [22].
	*Acinetobacter* spp.	2	39	8	3	3		Effect of the combination indeterminate on 33 isolates.
	*Staphylococcus* spp.	12	284	57	12	9	1	Great heterogeneity of results.
	*Streptococcus* spp.	6	63	33	59	8		-
	*Enterococcus* spp.	2	77	78	0	22		-
	*N. gonorrhoeae*	3	44	0	5	95		-
Carbapenems	Enterobacterales	23	542	43	37	19		
	*P. aeruginosa*	15	445	29	25	36	1	-
	*Acinetobacter* spp.	5	103	28	17	22		-
	Gram + cocci	12	231	56	13	22	8	*S. aureus, S. epidermidis, Enterococci* spp., *S. pneumoniae*. High rates of antagonistic effect reported on *E. faecalis* isolates.
	*N. gonorrhoeae*	1	4	0	75	25		-
Monobactams	Enterobacterales	4	71	15	27	45		-
	*P. aeruginosa*	3	138	29	54	17		-
Quinolones	Enterobacterales	6	264	17	12	69		-
	*P. aeruginosa*	18	263	42	36	38	5	Synergism rates not concordant in all studies.
	*Acinetobacter* spp.	3	41	2	10	7		-
	*Staphylococcus* spp.	7	90	37	9	34		-
	*N. gonorrhoeae*	1	4	0	0	100		-
Aminoglycosides	Enterobacterales	19	713	20	31	36		Synergism rates not concordant in all studies.
	*P. aeruginosa*	23	440	43	29	27	1	Synergism rates not concordant in all studies.
	*Acinetobacter* spp.	5	102	37	5	18		Synergism rates not concordant in all studies.
	*S. aureus*	8	301	26	4	53	1	Antagonistic effect of FOS + gentamicin against 4 isolates [12].
	*Streptococcus* spp.	1	16	0	52	48		-
	*E. faecium*	1	8	62	13	25		-
	*N. gonorrhoeae*	1	4	0	25	75		-
	*H. influenzae*	1	1	0	0	100		-
Glycopeptides	*A. baumannii*	1	20	0	0	100		-
	*Staphylococcus* spp.	12	229	17	16	65	2	In 2 studies [69,176] VAN exhibited higher synergistic rates than TEC. Antagonistic effect with FOS + VAN against 5 isolates of *S. aureus* [12,43].
	*Enterococcus* spp.	2	55	55	22	24		-
	*S. pneumoniae*	2	28	4	4	92		-
Macrolides	Enterobacterales	1	87	53	34	14		-
	*N. gonorrhoeae*	2	12	0	0	100		-
	*P. aeruginosa*	2	31	19	79	2		-
	*S. aureus*	1	34	26	68	6		-
	*S. epidermidis*	1	11	0	0	100		-
	*S. pseudointermedius*	1	8	62	25	12		-
	*Streptococcus* spp.	1	26	15	27	58		Only erythromycin was tested in combination with FOS. Against almost half of strains additive or, less frequently, synergistic effect was observed.
Tetracyclines	Enterobacterales	2	120	5	11	84		Indifferent effect when tetracycline was tested, but one study showed additive or synergistic effect when using minocycline + FOS combination [38].
	*P. aeruginosa*	1	5	40	60	0		-
	*Acinetobacter* spp.	1	25	12	56	32		In all experiment minocycline susceptibility restoration was observed [65].
	*S. aureus*	3	152	72	1	27		-
	*Enterococcus* spp.	3	75	24	10	20		Indifferent effect when minocycline was tested, but one study showed additive or synergistic effect when using doxicycline + FOS combination [68].
	*N. gonorrhoeae*	1	4	0	0	100		-
Polymyxins	Enterobacterales	18	381	26	35	35	4	Antagonistic effect of FOS + colistin observed against 14 isolates of *K. pneumoniae*.
	*P. aeruginosa*	4	111	27	41	31		-
	*Acinetobacter* spp.	12	206	19	15	32	1	Antagonistic effect of FOS + colistin observed against 3 isolates of *A. baumannii*.
	*N. gonorrhoeae*	1	4	0	0	100		-
Daptomycin	*Staphylococcus* spp.	13	186	56	31	14		-
	*Enterococcus* spp.	5	49	97	0	3		-
Tigecycline	Enterobacterales	9	313	17	44	34	1	One in vivo study observed indifferent effect in 100% of cases against *E. coli* [73] and one in vitro study reported 2 cases of antagonistic effect against *K. pneumoniae* isolates [89].
	*P. aeruginosa*	1	15	13	0	87		-
	*Acinetobacter* spp.	2	25	0	16	4		-
	*S. aureus*	2	48	21	0	79		Conflicting results (total indifference or almost total synergistic effect).
	*Enterococcus* spp.	3	61	61	0	9		-
	*N. gonorrhoeae*	1	4	0	0	100		-
Linezolid	*Enterococcus* spp.	4	69	17	29	6		Synergistic effect was never observed for *E. faecalis* (2 studies) [13,201].
	*S. aureus*	9	166	74	2	2		-
	*S. epidermidis*	1	2	100	0	0		-
Rifampin	*E. coli*	1	17	6	53	41		-
	*A. baumannii*	1	20	0	0	100		-
	*S. aureus*	9	114	35	21	4	3	Antagonistic effect of FOS + RIF against 3 isolates [35,37].
	*S. epidermidis*	2	5	40	0	40		-
	*Streptococcus* spp.	2	29	3	0	97		-
	*Enterococcus* spp.	2	50	59	0	12		-
	*N. gonorrhoeae*	1	4	25	0	75		-
Metronidazole	Intestinal bacteria (not specified)	1	NA	-	-	-		-
	*H. pylori*	1	24	0	21	80		-
Spectinomycin	*N. gonorrhoeae*	1	4	0	0	100		-
Sulbactam	*A. baumannii*	1	8	75	0	25		-
Lincomycin	*S. aureus*	1	37	81	19	0		-
Nitroxoline	*P. aeruginosa*	1	8	12	0	88		-
Dalfopristin-Quinupristin	*Staphylococcus* spp.	2	12	100	0	0		-
Fusidic acid	*S. aureus*	3	239	63	4	33		-
Chloramphenicol	Enterobacterales	4	468	39	34	25		-
	*P. aeruginosa*	1	19	53	37	10		-
	*S. aureus*	1	48	44	37	19		-
Nitrofurantoin	Enterobacterales	1	100	0	0	100		-
	*Enterococcus* spp.	1	32	0	0	100		-
Trimethoprim-Sulfamethoxazole	Enterobacterales	2	120	2	5	89		-
	*S. aureus*	1	148	3	0	95	3	Antagonistic effect was reported for 4 isolates [12].

## Supplementary Materials

The following are available online at https://www.mdpi.com/2079-6382/9/8/500/s1, Table S1: Studies on combination between fosfomycin and different antibiotics. CB: checkerboard assay; TK: time–kill assay; ET: E-test, Table S2: Studies on combination between fosfomycin and molecules other than antibiotics. CB: checkerboard assay; TK: time–kill assay.
