# Supplementary material for: Fosfomycin as Partner Drug for Systemic Infection Management. A Systematic Review of Its Synergistic Properties from In Vitro and In Vivo Studies
Source: Antibiotics (Basel). 2020 Aug 10;9(8):500. doi: 10.3390/antibiotics9080500 (PMC7460049; doi:10.3390/antibiotics9080500)
Supplement: Supplementary file 1 [file antibiotics-09-00500-s001.pdf]

Supplementary materials

Table S1. Studies on combination between fosfomycin and different antibiotics. CB: checkerboard assay; TK: time-kill assay; ET: E-test.

Strain	Year and Country	Author	Antibiotic	Number of isolates	Known Resistance Mechanisms or Determinants (%)	FOS-resistant (%)	Other drug-resistant (%)	In Vitro (Methods)/in Vivo (animal and Site of Infection)	Synergistic effect (%)	Additive Effect (%)	Indifferent Effect (%)	Antagonistic Effect (%)	FOS Susceptibility Restoration (%)	Other drug Susceptibility Restoration (%)	Comments	Reference
H. pylori	2005, Austria	Blacky	Metronidazole	24	-	0%	33%	in vitro (CB)	0%	21%	100%	4%	-	-	-	[1]
Intestinal bacteria (not specified)	1984, Sweden	Nord	Metronidazole	-	-	-	-	in vivo (rats, intra-abdominal infection)	-	-	-	-	-	-	Animal treated with MTZ + FOS showed a significantly decrease mortality and increase cure rates. Mortality with MTZ alone was 40%, FOS alone 33%, MTZ + FOS 7%.	[2]
N. gonorrhoeae	2015, The Netherlands	Wind	Spectinomycin	4	-	-	-	in vitro (ET)	-	-	-	-	-	-	Only 1/4 isolate showed a FIC < 1. If the FICI was < 1.0 in at least 3 of the 4 tested isolates for a specific combination, that combination was re-tested with microdilution method. The combination FOS + SCM none are promising candidates for gonorrhoea dual therapy.	[3]
A. baumannii	2011, USA	Santimaleeworagun	Sulbactam	8	OXA-23 (100%)	100%	75%	in vitro (CB, TK)	75%	0%	25%	0%	-	-	-	[4]
S. aureus	1978, Spain	Olay	Lincomycin	37	-	-	-	in vitro (CB)	81%	19%	0%	0%	-	-	-	[5]
P. aeruginosa (n=1) and other (not specified)	1977, Spain	Daza	Nitroxoline	8	-	100%	-	in vitro (CB in agar)	12%	0%	88%	0%	-	-	-	[6]
S. aureus	2008, France	Duez	Dalfopristin-Quinupristin	6	MRSA (50%)	0%	67%	in vitro (CB)	100%	0%	0%	0%	-	-	-	[7]
S. aureus, S. epidermi	2001, Austria	Grif	Dalfopristin-Quinupristin	6	MRSA (17%), GISA (17%)	-	17%	in vitro (CB, TK)	100%	0%	0%	0%	-	-	-	[8]

dis																
S. aureus	2012, Taiwan	Tang	Fusidic acid	8	MRSA (100%)	6%	12%	in vitro (TK)	100%	0%	0%	0%	-	-	-	[9]
S. aureus	2010, China	Yu	Fusidic acid	196	MRSA (100%)	100 %	100 %	in vitro (CB)	88%	12%	0%	0%	100%	100%	-	[10]
S. aureus	1994, France	Drugeon	Fusidic acid	35	MRSA (67%)	0%	100 %	in vitro (double diluition method)	0%	0%	100%	0%	-	-	Synergism: decrease of at least 16% after 6 h in the AUC of survivors and at least a 100-fold increase in killing after 24 h. Additive/indifferent: reduced by less than 10% after 6 h in the AUC of survivors and increase in killing was less than ten-fold after 24 h. Antagonism: increase of more than 16% in the number of survivors after 6 h and a 100-fold increase in viable bacteria after 24 h.	[11]
Enterobacterales	1977, Spain	Daza	Chloramphenicol	100	-	100 (100 %)	-	in vitro (CB)	0%	-	100%	0%	-	-	Authors considered synergistic effect when MIC was at least fourfold lower over initial MIC.	[6]
E. coli	1978, Spain	Olay	Chloramphenicol	90	-	-	-	in vitro (CB)	37,8%	Partial Synergy: 47,8%	14,4%	0%	-	-	Authors considered **Synergistic effect** when MIC of both antimicrobials was at least fourfold lower over initial MIC; **partial synergy** when MIC of one antimicrobials was at least fourfold lower and MIC of the other one 2 times lower over initial MIC; **Indifferent effect** when MIC of both antimicrobials was 2 times lower; **antagonism** when MIC of both increased 4 times over initial MIC.	[5]
Klebsiella spp.	1978, Spain	Olay	Chloramphenicol	56	-	-	-	in vitro (CB)	35,7%	Partial Si ynergy: 41,1%	23,2%	0%	-	-	Authors considered **Synergistic effect** when MIC of both antimicrobials was at least fourfold lower over initial MIC; **partial synergy** when MIC of one antimicrobials was at least fourfold lower and MIC of the other one 2 times lower over initial MIC; **Indifferent effect** when MIC of both antimicrobials was 2 times lower; **antagonism** when MIC of both increased 4 times over initial MIC.	[5]
Proteus spp. *(Indole +)*	1978, Spain	Olay	Chloramphenicol	34	-	-	-	in vitro (CB)	55,9%	Partial Synergy: 20,6%	23,5%	0%	-	-	Authors considered **Synergistic effect** when MIC of both antimicrobials was at least fourfold lower over initial MIC; **partial synergy** when MIC of one antimicrobials was at least fourfold lower and MIC of the other one 2 times lower over initial MIC; **Indifferent effect** when MIC of both antimicrobials was 2 times lower; **antagonism** when MIC of both increased 4 times over initial MIC.	[5]
P. aerugino	1978, Spain	Olay	Chloramphenicol	19	-	-	-	in vitro (CB)	52,6%	Partial Synergy:	10,5%	0%	-	-	Authors considered **Synergistic effect** when MIC of both antimicrobials was at least fourfold lower over initial MIC; **partial synergy** when MIC of one	[5]

sa										36,9%					antimicrobials was at least fourfold lower and MIC of the other one 2 times lower over initial MIC; **Indifferent effect** when MIC of both antimicrobials was 2 times lower; **antagonism** when MIC of both increased 4 times over initial MIC.	
Salmonella spp.	1978, Spain	Olay	Chloramphenicol	32	-	-	-	in vitro (CB)	28,1%	Partial Synergy: 62,5%	9,4%	0%	-	-	Authors considered **Synergistic effect** when MIC of both antimicrobials was at least fourfold lower over initial MIC; **partial synergy** when MIC of one antimicrobials was at least fourfold lower and MIC of the other one 2 times lower over initial MIC; **Indifferent effect** when MIC of both antimicrobials was 2 times lower; **antagonism** when MIC of both increased 4 times over initial MIC.	[5]
	1978, Spain	Perea	Chloramphenicol	90 (2 TK)	-	10%	27%	in vitro (CB, TK)	CB: 17 (18,9%) / TK: 1 (50%)	CB: 48 (53,3%) / TK: 1 (50%)	CB: 13 (14,4%) / TK: 0%	0%	-	-	CK: 12 strains reported as "Not Evaluable"; Only 2 strains evaluated with TK.	[12]
S. typhi	1977, Spain	Figueroa	Chloramphenicol	16	-	0%	-	in vitro (CB)	14 (87,5%)	2 (12,5%)	0%	0%	-	-	The authors considered synergistic the effect for FICI up to < 1 and additive the effect for FICI = 1. They also evaluated different antibiotic combinations in clinical experience on patients with typhoid fever: FOS + AMP in higher rates of cure then FOS + CAF combination.	[13]
Shighella spp.	1978, Spain	Perea	Chloramphenicol	50	-	54%	56%	in vitro (CB, TK)	CB: 11 (22%) / TK: 1 (50%)	CB: 28 (56%) / TK: 0%	CB: 9 (18%) / TK: 1 (50%)	0%	-	-	CK: 2 strains reported as "Not Evaluable"; Only 2 strains evaluated with TK.	[12]
S. aureus	1978, Spain	Olay	Chloramphenicol	48	-	-	-	in vitro (CB)	21 (43,8%)	Partial Synergy: 18 (37,5%)	9 (18,7%)	0%	-	-	Authors considered **Synergistic effect** when MIC of both antimicrobials was at least fourfold lower over initial MIC; **partial synergy** when MIC of one antimicrobials was at least fourfold lower and MIC of the other one 2 times lower over initial MIC; **Indifferent effect** when MIC of both antimicrobials was 2 times lower; **antagonism** when MIC of both increased 4 times over initial MIC.	[5]
Enterobacterales	1977, Spain	Daza	Nitrofurantoin	100	-	100 (100%)	-	in vitro (CB)	0%	-	100%	0%	-	-	Authors considered synergistic effect when MIC was at least fourfold lower over initial MIC.	[6]
E. faecium	2012, USA	Descouzouez	Nitrofurantoin	32	VRE (100%)	9%	100%	in vitro (TK)	0%	0%	100%	0%	-	-	The authors considered MIC ≤ 64 mg/L as FOS breakpoint.	[14]
Enterobacterale	2019, USA	Flamm	TMP/SMX	20	7/30 MDR strains (*A. baumannii*, Enterobacterales and	-	-	in vitro (CB)	2 (10%)	6 (30%)	7 (35%)	0%	-	-	Authors considered Partial Sinergy when FICI was between 0.5–1 and Additive effect for FICI = 1. Results for	[15]

s					*P. aeruginosa*) included 2 ESBL and 2 KPC Enterobacterales										5/20 strains (25%) were indeterminate.	
	1977, Spain	Daza	TMP/SMX	100	-	100 (100 %)	-	in vitro (CB)	0%	-	100%	0%	-	-	Authors considered synergistic effect when MIC was at least fourfold lower over initial MIC.	[6]
S. aureus	1985, USA	Alvarez	TMP/SMX	148	MRSA (100%)	-	-	in vitro (Microtiter technique 1:1 ratio)	4 (2,7%)	-	140 (94,6%)	4 (2,7%)	-	-	Authors considered Synergistic effect when MIC of both antimicrobials was 1/4 MIC lower, antagonism when MIC of at least 1 antimicrobial was four times higher and Indifferent all other cases.	[16]

Table S2. Studies on combination between fosfomycin and molecules other than antibiotics. CB: checkerboard assay; TK: time-kill assay.

Strain	Year and Country	Author	Molecule	Number of Isolates	Known Resistance Mechanisms or Determinants (%)	FOS-resistant (%)	In Vitro (Methods)/in Vivo (Animal and Site of Infection)	Synergistic Effect (%)	Additive Effect (%)	Indifferent Effect (%)	Antagonistic Effect (%)	FOS Susceptibility Restoration (%)	Comments	Reference
L. monocytogenes	2020, USA	Zhang	Chlorogenic acid, Caffeic acid	NS		100%	in vitro (bacterial cell growth study)	100%	0%	0%	0%	Chlorogenic acid: 100% (if chlorogenic acid was 3 mg/L)	Reduction in the cell growth equal to 98% with chlorogenic acid and 85,2% with caffeic acid.	[17]
S. aureus, S. enterica subsp. Bukuru, E. coli	2020, Egypt	Abo-Shama	Silver (AgNPs) and zinc oxide (ZnONPs) nanoparticles	3	-	-	in vitro (disk diffusion)	AgNPs: 100% ZnONPs: 100%	0%	0%	0%	-	-	[18]
S. aureus, S. epidermidis	2019, China	She	Auranofin	4	MRSA (75%)	0%	in vitro (CB); in vivo (mouse, cutaneous infection model)	50%	25%	25%	0%	-	AF combined with FOS synergistically inhibited abscess and inflammation formation and reduced the bacterial load for both MSSA and MRSA strains.	[19]
P. aeruginosa	2019, Canada	Domalaon	Dilipid ultrashort cationic lipopeptides	1	-	-	in vitro (CB)	100%	0%	0%	0%	-	-	[20]
P. aeruginosa	2018, Canada	Yang	Tobramycin-efflux pump inhibitor conjugates	1	-	0%	in vitro (CB)	100%	0%	0%	0%	100%	-	[21]
P. aeruginosa	2017, Canada	Lyu	Amphiphilic lysine-tobramycin conjugates	1	-	0%	in vitro (CB)	100%	0%	0%	0%	100%	-	[22]
S. aureus	2017, Iran	Akbari-Ayezloy	β-chloro-L-alanine	11	MRSA (100%)	9%	in vitro (tube diluition method)	100%	0%	0%	0%	-	The authors used the ANOVA analysis to evaluate the effectiveness of the combination. The combination reduces the formation of biofilm.	[23]
E. faecalis	2015, France	Breidenstein	Plectasin NZ2114	1	VanA (100%)	-	in vitro (disk diffusion, CB, TK)	0%	0%	100%	0%	-	The authors stated there was not synergistic effect, without specifying if the effect was additive, indifferent or antagonistic.	[24]
Enterococcus spp., S. aureus	2008, Japan	Sakagami	2 Quinolone derivatives: A and B	14	MRSA (64%) VRE (36%)	-	in vitro (doubling dilution method)	A: 100% B: 64% (MRSA)	A: 0% B: 36% (VRE)	A: 0% B: 0%	A: 0% B: 0%	-	Compound A: 8-[3-[(ethylamino) methyl] -1-pyrrodinyl] -7-fluoro-9,1-[(Nmethylimino) methano] -5-oxo-5H-thiazolo [3, 2-a] quinolone-4-carboxylic acid; Compound B: 7-fluoro-8-morpholino-9, 1-[(N-methylimino)methano] -5-oxo -5H- thiazolo [3, 2-a]	[25]

													quinolone-4-carboxylic acid.	
E. coli	2003, Italy	Marchese	N-acetylcysteine	4	-	-	in vitro (formation or disruption of biofilms)	-	-	-	-	-	FOS plus N-acetylcysteine reduced biofilm; the most effective combination was that obtained using FOS at 2000 mg/l and NAC at 2 mg/ml.	[26]
S. aureus	1998, Japan	Sakagami	Sophoraflavanone G	6	MRSA (100%)	-	in vitro (doubling dilution method)	100%	0%	0%	0%	-	-	[27]
P. vulgaris, S. gallinarum	1988, USA	Dulaney	Arenaemycin	2	-	0%	in vitro (doubling dilution method)	100%	0%	0%	0%	-	-	[28]

References

1. Blacky, A.; Makristathis, A.; Apfalter, P.; Willinger, B.; Rotter, M.L.; Hirschl, A.M. In vitro activity of fosfomycin alone and in combination with amoxicillin, clarithromycin and metronidazole against Helicobacter pylori compared with combined clarithromycin and metronidazole. *Eur. J. Clin. Microbiol. Infect. Dis.* **2005**, *24*, 276–279, doi:10.1007/s10096-005-1307-9.
2. Nord, C.; Lahnborg G Efficacy of Metronidazole and Fosfomycin Alone and in Combination in the Treatment of Experimentally Induced Intra-Abdominal Infections. *Scand J Gastroenterol Suppl.* **1984**, *90*, 15–19, doi:10.1086/527442.
3. Wind, C.M.; De Vries, H.J.C.; Van Dam, A.P. Determination of in vitro synergy for dual antimicrobial therapy against resistant Neisseria gonorrhoeae using Etest and agar dilution. *Int. J. Antimicrob. Agents* **2015**, *45*, 305–308, doi:10.1016/j.ijantimicag.2014.10.020.
4. Santimaleeworagun, W.; Wongpoowarak, P.; Chayakul, P.; Pattharachayakul, S.; Tansakul, P.; Garey, K.W. In vitro activity of colistin or sulbactam in combination with fosfomycin or imipenem against clinical isolates of carbapenem-resistant acinetobacter baumannii producing OXA-23 carbapenemases. *Southeast Asian, J. Trop. Med. Public Health* **2011**, *42*, 890–900.
5. Olay, T.; Rodriguez, A.; Oliver, L.E.; Vicente, M.V.; Quecedo, M.C.R. Interaction of fosfomycin with other antimicrobial agents: In vitro and in vivo studies. *J. Antimicrob. Chemother.* **1978**, *4*, 569–576, doi:10.1093/JAC.
6. Daza, R.; Moreno Lopez, M.; Damaso, D. Interactions of fosfomycin with other antibiotics. *Chemotherapy* **1977**, *23*, 86–92, doi:10.1159/000222031.
7. Duez, J.M.; Adochitei, A.; Péchinot, A.; Siebor, E.; Sixt, N.; Neuwirth, C. In vitro combinations of five intravenous antibiotics with Dalfopristin-Quinupristin against staphylococcus aureus in a 3-dimensional model. *J. Chemother.* **2008**, *20*, 684–689, doi:10.1179/joc.2008.20.6.684.
8. Grif, K.; Dierich, M.; Pfaller, K.; Miglioli, P.; Allerberger, F. In vitro activity of fosfomycin in combination with various antistaphylococcal substances. *J. Antimicrob. Chemother.* **2001**, *48*, 209–217, doi:10.1093/JAC.
9. Tang, H.-J.; Chen, C.-C.; Cheng, K.-C.; Toh, H.-S.; Su, B.-A.; Chiang, S.-R.; Ko, W.-C.; Chuang, Y.-C. In vitro efficacy of fosfomycin-containing regimens against methicillin-resistant Staphylococcus aureus in biofilms. *J. Antimicrob. Chemother.* **2012**, *67*, 944–950, doi:10.1093/jac/dkr535.
10. Yu, X.H.; Song, X.J.; Cai, Y.; Liang, B.B.; Lin, D.F.; Wang, R. In vitro activity of two old antibiotics against clinical isolates of methicillin-resistant Staphylococcus aureus. *J. Antibiot. (Tokyo).* **2010**, *63*, 657–659, doi:10.1038/ja.2010.105.
11. Drugeon, H.; Caillon, J.; Juvin, M. In-vitro Antibacterial Activity of Fusidic Acid Alone and in Combination With Other Antibiotics Against Methicillin-Sensitive and -Resistant Staphylococcus Aureus. *J. Antimicrob. Chemother.* **1994**, *34*, 899–907, doi:10.1016/S0924-8579(98)00072-7.
12. Perea, E.J.; Torres, M.A.; Borobio, M.V. Synergism of fosfomycin-ampicillin and fosfomycin-chloramphenicol against Salmonella and Shigella. *Antimicrob. Agents Chemother.* **1978**, *13*, 705–709, doi:10.1128/AAC.13.5.705.

13. Figueroa, J.; Baquero, G.; Otal, C.; Rodríguez, A. Treatment of typhoid fever with fosfomycin alone and associated to chloramphenicol or ampicillin. *Chemotherapy* **1977**, *23*, 365–377, doi:10.1159/000222077.
14. Descourouez, J.L.; Jorgenson, M.R.; Wergin, J.E.; Rose, W.E. Fosfomycin synergy in vitro with amoxicillin, daptomycin, and linezolid against vancomycin-resistant enterococcus faecium from renal transplant patients with infected urinary stents. *Antimicrob. Agents Chemother.* **2013**, *57*, 1518–1520, doi:10.1128/AAC.02099-12.
15. Flamm, R.K.; Rhomberg, P.R.; Lindley, J.M.; Sweeney, K.; Ellis-Grosse, E.J.; Shortridge, D. Evaluation of the Bactericidal Activity of Fosfomycin in Combination with Selected Antimicrobial Comparison Agents Tested against Gram-Negative Bacterial Strains by Using Time–kill Curves. *Antimicrob. Agents Chemother.* **2019**, *63*, e02549-18, doi:10.1128/AAC.02549-18.
16. Alvarez, S.; Jones, M.; Berk, S.L. In vitro activity of fosfomycin, alone and in combination, against methicillin-resistant Staphylococcus aureus. *Antimicrob. Agents Chemother.* **1985**, *28*, 689–690, doi:10.1128/AAC.28.5.689.
17. Zhang, F.; Zhai, T.; Haider, S.; Liu, Y.; Huang, Z.J. Synergistic Effect of Chlorogenic Acid and Caffeic Acid with Fosfomycin on Growth Inhibition of a Resistant Listeria monocytogenes Strain. *ACS Omega* **2020**, *5*, 7537–7544 doi:10.1021/acsomega.0c00352.
18. Abo-Shama, U.H.; El-Gendy, H.; Mousa, W.S.; Hamouda, R.A.; Yousuf, W.E.; Hetta, H.F.; Abdeen, E.E. Synergistic and antagonistic effects of metal nanoparticles in combination with antibiotics against some reference strains of pathogenic microorganisms. *Infect. Drug Resist.* **2020**, *13*, 351–362, doi:10.2147/IDR.S234425.
19. She, P.; Zhou, L.; Li, S.; Liu, Y.; Xu, L.; Chen, L.; Luo, Z.; Wu, Y. Synergistic Microbicidal Effect of Auranofin and Antibiotics Against Planktonic and Biofilm-Encased S. aureus and E. faecalis. *Front. Microbiol.* **2019**, *10*, 2453, doi:10.3389/fmicb.2019.02453.
20. Domalaon, R.; Brizuela, M.; Eisner, B.; Findlay, B.; Zhanel, G.G.; Schweizer, F. Dilipid ultrashort cationic lipopeptides as adjuvants for chloramphenicol and other conventional antibiotics against Gram-negative bacteria. *Amino Acids* **2019**, *51*, 383–393, doi:10.1007/s00726-018-2673-9.
21. Yang, X.; Domalaon, R.; Lyu, Y.; Zhanel, G.; Schweizer, F. Tobramycin-Linked Efflux Pump Inhibitor Conjugates Synergize Fluoroquinolones, Rifampicin and Fosfomycin against Multidrug-Resistant Pseudomonas aeruginosa. *J. Clin. Med.* **2018**, *7*, 158, doi:10.3390/jcm7070158.
22. Lyu, Y.; Domalaon, R.; Yang, X.; Schweizer, F. Amphiphilic lysine conjugated to tobramycin synergizes legacy antibiotics against wild-type and multidrug-resistant pseudomonas aeruginosa. *Pept. Sci.* **2019**, *111*, e23091, doi:10.1002/bip.23091.
23. Akbari-Ayezloy, E.; Hosseini-Jazani, N.; Yousefi, S.; Habibi, N. Eradication of methicillin resistant S. Aureus biofilm by the combined use of fosfomycin and β-chloro-L-alanine. *Iran. J. Microbiol.* **2017**, *9*, 1–10.
24. Breidenstein, E.B.M.; Courvalin, P.; Meziane-Cherif, D. Antimicrobial activity of plectasin NZ2114 in combination with cell wall targeting antibiotics against VanA-type enterococcus faecalis. *Microb. Drug Resist.* **2015**, *21*, 373–379, doi:10.1089/mdr.2014.0221.
25. Sakagami, Y.; Komemushi, S.; Tsukamoto, G.; Kondo, H.; Yoshikawa, A.; Muraoka, O. Anti-vRE and anti-MRSA activities of new quinolones and their synergism with commercial antibiotics. Part 2. *Biocontrol Sci.* **2008**, *13*, 103–109.
26. Marchese, A.; Bozzolasco, M.; Gualco, L.; Debbia, E.A.; Schito, G.C.; Schito, A.M. Effect of fosfomycin alone and in combination with N-acetylcysteine on E. coli biofilms. *Int. J. Antimicrob. Agents* **2003**, *22*, 95–100, doi:10.1016/S0924-8579(03)00232-2.
27. Sakagami, Y.; Mimura, M.; Kajimura, K.; Yokoyama, H.; Iinuma, M.; Tanaka, T.; Ohyama, M. Anti-MRSA activity of sophoraflavanone G and synergism with other antibacterial agents. *Lett. Appl. Microbiol.* **1998**, *27*, 98–100, doi:10.1046/j.1472-765X.1998.00386.x.
28. Dulaney, E.L.; Jacobsen, C.A. Synergy between fosfomycin and arenaemycin. *J. Antibiot. (Tokyo).* **1988**, *41*, 982–983, doi:10.7164/antibiotics.41.982.
